# Supplementary material for: Systematic review and integrated data analysis reveal diverse pangolin-associated microbes with infection potential
Source: Nat Commun. 2023 Oct 25;14:6786. doi: 10.1038/s41467-023-42592-w (PMC10600157; doi:10.1038/s41467-023-42592-w)
Supplement: Supplementary file 2 — Supplementary Information [file 41467_2023_42592_MOESM2_ESM.pdf]

## Supplementary Information

Supplementary Text 1. Basic characteristics of data collection........................................................1
Supplementary Fig. 1. Geographic distribution of *Manis pentadactyla* and *Manis javanica.* ..........2
Supplementary Fig. 2. Geographic distribution of *Manis crassicaudata* and *Manis culionensis* .....3
Supplementary Fig. 3. Geographic distribution of *Phataginus tricuspis* ..........................................4
Supplementary Fig. 4. Geographic distribution of *Smutsia gigantea* ...............................................5
Supplementary Fig. 5. Geographic distribution of *Smutsia temminckii*............................................6
Supplementary Fig. 6. Geographic distribution of *Phataginus tetradactyla*.....................................7
Supplementary Table 1. Pangolin samples used for detecting microbes...........................................8
Supplementary Table 2. Extracting source of microbe species information ..................................13
Supplementary Table 3. Sequence information of pangolin-associated microbes ..........................17
Supplementary Fig. 7. Meta-analysis of the prevalence of each family of pangolin-associated microbes.........................................................................................................................................28
Supplementary Table 4. Positive rate of pangolin-associated microbes ........................................32
Supplementary Fig. 8. Meta-analysis of the prevalence of each species of pangolin-associated microbes.........................................................................................................................................35
Supplementary Table 5. Univariable meta-regression of each family of pangolin-associated microbes.........................................................................................................................................38
Supplementary Table 6. Univariable meta-regression of each species of pangolin-associated microbes.........................................................................................................................................40
Supplementary Fig. 9 Phylogenetic tree of *Betacoronavirus*..........................................................41
Supplementary Fig. 10. Phylogenetic tree of *Parvoviridae* ............................................................42
Supplementary Fig. 11. Phylogenetic tree of animal-associated microbes.....................................43
Supplementary Fig. 12. Phylogenetic tree of pangolin-associated microbes..................................44
Supplementary Fig. 13. Phylogenetic tree of pangolin-associated microbes..................................45
Supplementary Fig. 14. Phylogenetic tree of pangolin-associated microbes..................................46
Supplementary Table 7. Check list of Latin and common names of pangolins ..............................47
Supplementary Fig. 15. PRISMA flow diagram of study selection process..................................48
Supplementary Text 2. References for eight species of pangolins..................................................49
Supplementary Table 8. The gene or protein used to construct the phylogenetic tree of viruses....64

## Supplementary Text 1. Basic characteristics of data collection

For literature review, we identified 8,637 potentially relevant studies through database searching, of which 634 were from PubMed (in English), 4,385 from China National Knowledge Infrastructure (CNKI), 3,160 from WanFang database (in Chinese) and 458 from IUCN Red List of Threatened Species. After duplicates removal, 5,835 definitive records containing information on pangolin collections in English or Chinese were retrieved for eligibility assessment. Through intensive reading of the full text, 398 records were left for further full-text quality assessment according to the inclusion and exclusion criteria. Data on insufficiently detailed records (116 studies) of pangolin collection sites were further removed. Finally, 282 studies meeting our inclusion and exclusion criteria were recruited for further analysis. In this study, the literature we collected covered 60 countries around the world, including 8 species, with a total of 2,351 pangolin records (2337 records with location, 14 records from pangolins that did not provide a specific geographical origin of pangolins). In the website of Global Biodiversity Information Facility (GBIF), 759 georeferenced records were associated with 8 species of pangolins.

**a**

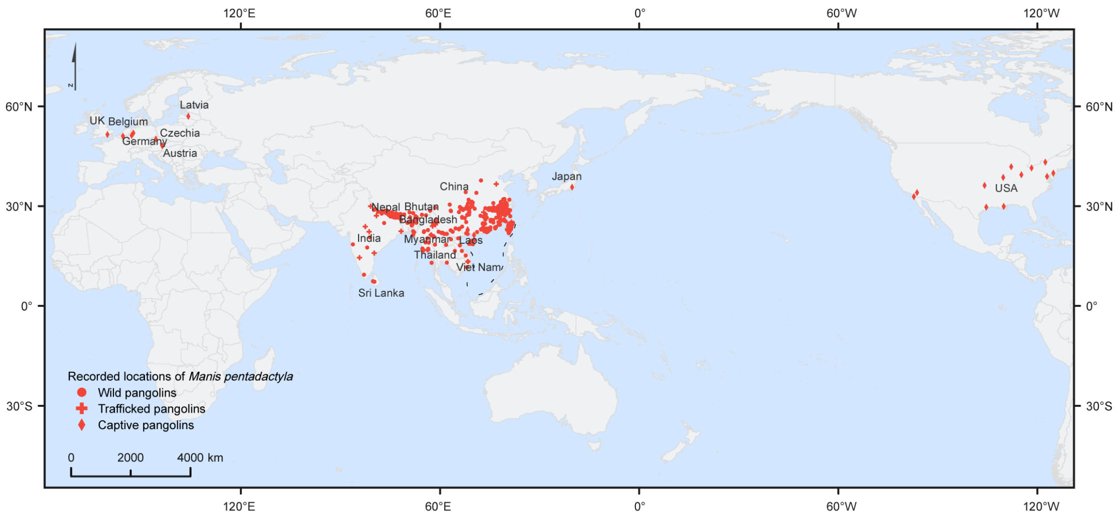


**b**

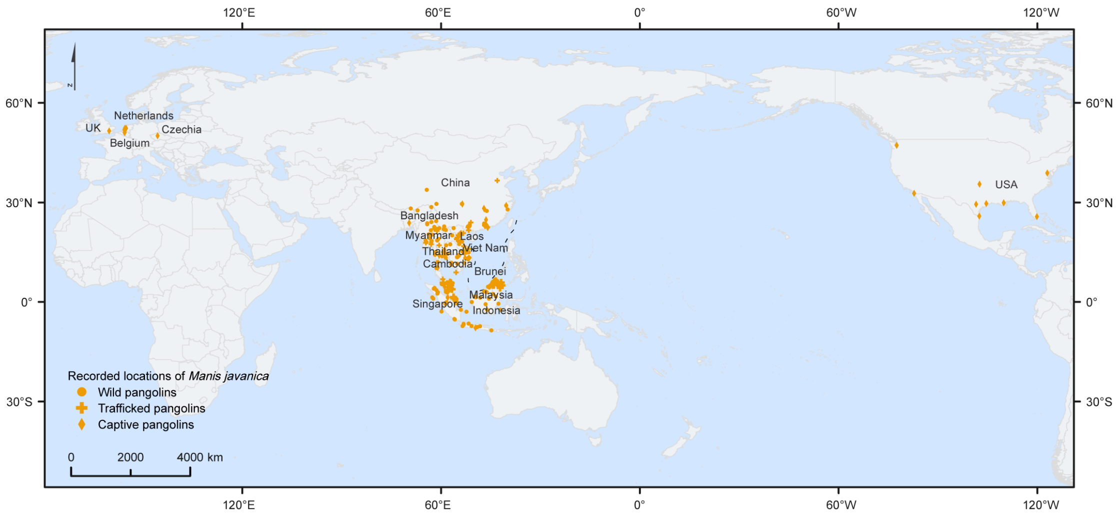


**Supplementary Fig. 1. Geographic distribution of *Manis pentadactyla* and *Manis javanica*.** **a**, Recorded locations of wild, trafficked and captive *Manis pentadactyla*. **b**, Recorded locations of wild, trafficked and captive *Manis javanica*.

**a**

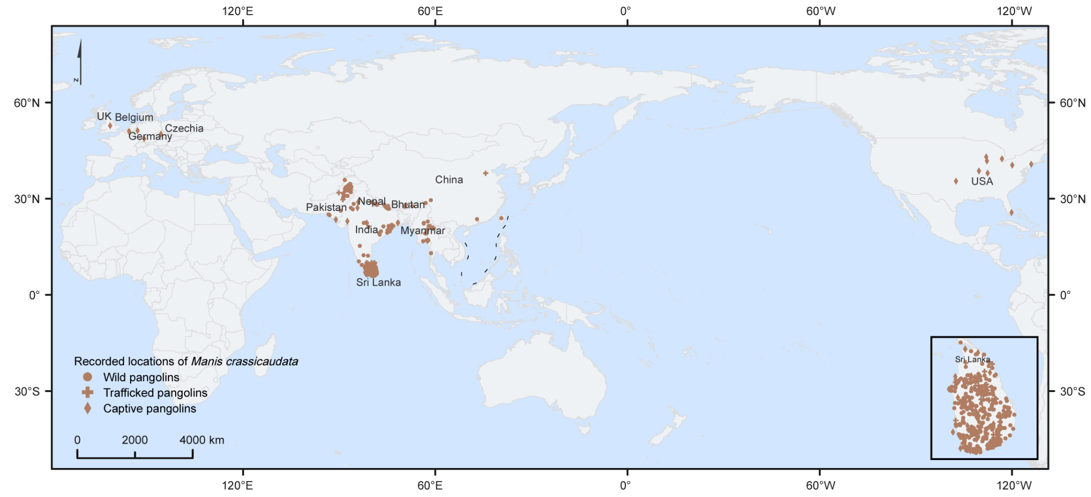


**b**

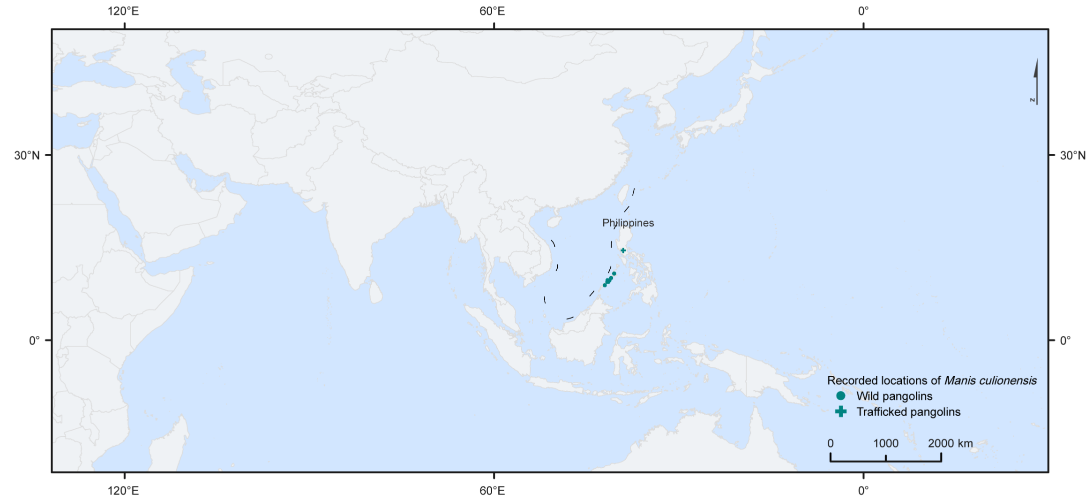


**Supplementary Fig. 2. Geographic distribution of *Manis crassicaudata* and *Manis culionensis*. a**, Recorded locations of wild, trafficked and captive *Manis crassicaudata.* **b**, Recorded locations of wild, trafficked and captive *Manis culionensis.*

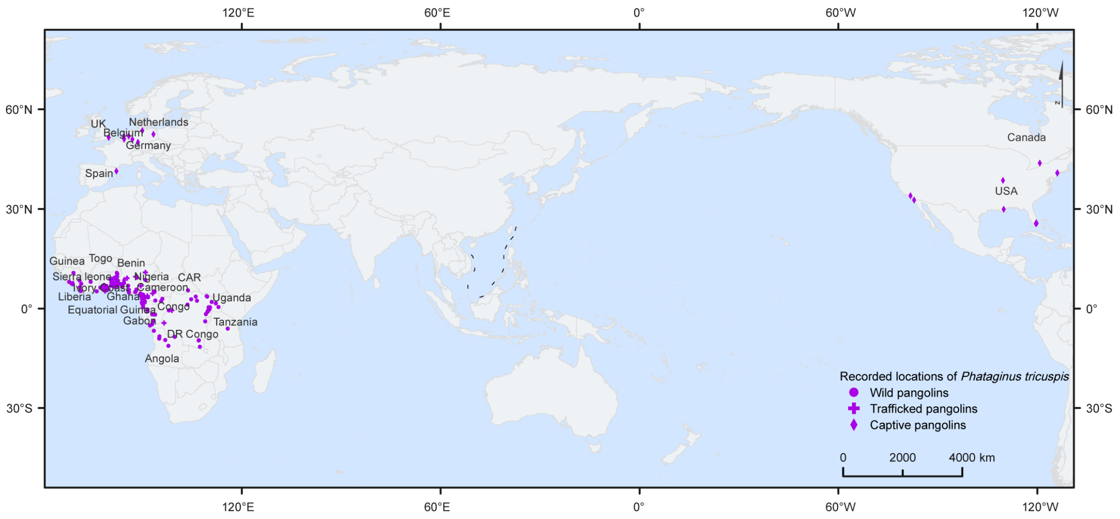


**Supplementary Fig. 3. Geographic distribution of *Phataginus tricuspis*.**

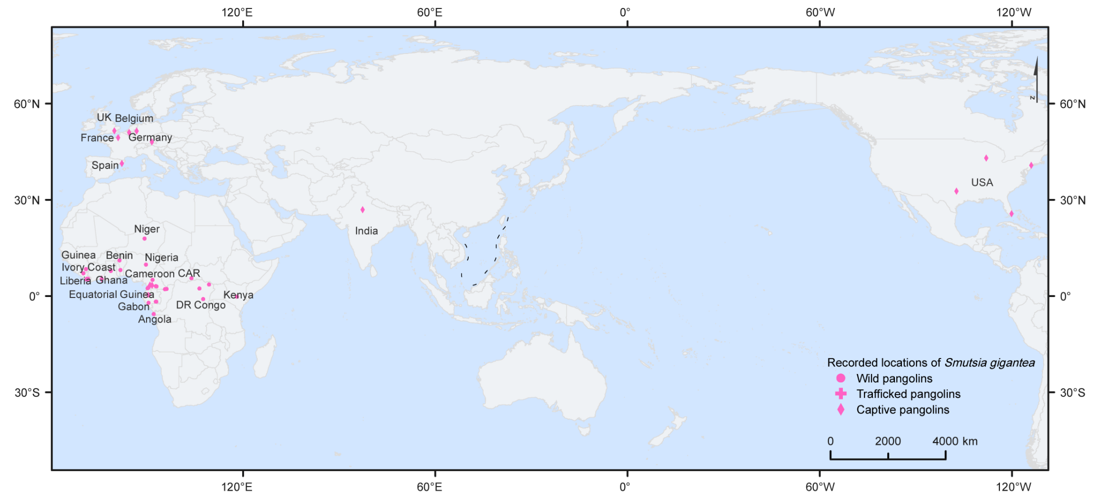


**Supplementary Fig. 4. Geographic distribution of *Smutsia gigantea*.**

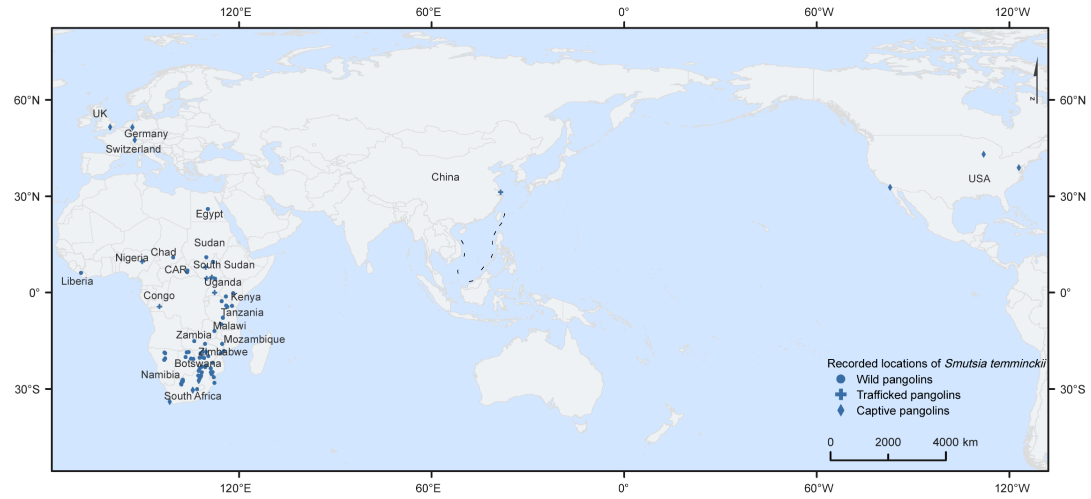


**Supplementary Fig. 5. Geographic distribution of *Smutsia temminckii*.**

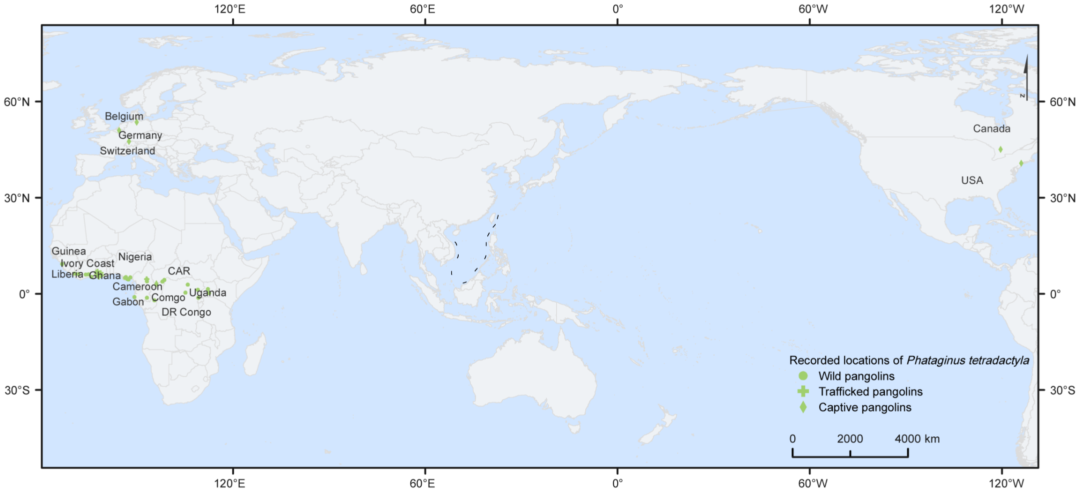


**Supplementary Fig. 6. Geographic distribution of *Phataginus tetradactyla*.**

**Supplementary Table 1. Pangolin samples used for detecting microbes**

| Microbe | Sample type |
|---|---|
| *Hukuchivirus IN93* | lung |
| *Catovirus CTV1* | lung |
| *Hokovirus HKV1* | lung |
| *Alphapapillomavirus 7* | lung |
| *Manis javanica papillomavirus 1* | muscle |
| *Manis pentadactyla papillomavirus 1* | muscle |
| *Pangolin circovirus* | sera |
| | anal swabs |
| | throat swabs |
| *Cyclovirus* sp. | lung |
| *Gemykibivirus* sp. | lung |
| *Pangolin chaphamaparvovirus BIME1* | mixed tissues (lung, heart, liver, spleen, kidney, intestine, muscle) |
| *Pangolin Copiparvovirus* | sera |
| *Pangolin copiparvovirus BIME1* | mixed tissues (lung, heart, liver, spleen, kidney, intestine, muscle) |
| *Pangolin copiparvovirus BIME2* | mixed tissues (lung, heart, liver, spleen, kidney, intestine, muscle) |
| *Carnivore protoparvovirus 1* | lung |
| | oesophagus |
| | trachea |
| | tongue |
| | heart |
| | mesenteric |
| | lymph node |
| | spleen |
| | liver |
| | adrenal glands |
| | brain |
| | spinal cord |
| | duodenum |
| | jejunum |
| | ileum |
| | intestine |
| | skeletal muscle |
| | mixed tissues (heart, liver, spleen, lung, kidney, intestine, stomach, muscle, brain) |
| *Pangolin densovirus* | anal swabs |
| | throat swabs |
| *Parus major densovirus* | lung |
| *Etatorquevirus* sp. | lung |
| *Tettorquevirus* sp. | lung |
| *Retroviridae* sp. | lung |

| | |
|---|---|
| *Pangolin picobirnavirus* | anal swabs |
| *Rotavirus A* | mixed tissues (lung, intestine, liver, spleen, kidney, muscle, heart) |
| *Mammalian orthoreovirus* | mixed tissues (intestine, liver, spleen, kidney, muscle, heart) |
| *Phocid orthoreovirus 1* | lung |
| *Lishui pangolin virus* | mixed tissues (blood, liver, spleen, lung, kidney, fecal) |
| | mixed tissues (lung, heart, liver, spleen, kidney, intestine, muscle) |
| *Pangolin Reoviridae* sp. | sera |
| *Japanese encephalitis virus* | mixed tissues (anal and throat swabs) |
| *Pangolin pestivirus* | lung |
| | kidney |
| | liver |
| | spleen |
| | anus |
| | rectum |
| | duodenum |
| | throat swabs |
| | anal swabs |
| *Pangolin pestivirus 1* | mixed tissues (heart, liver, spleen, lung, kidney, intestine, stomach, brain) |
| *Pangolin pestivirus 2* | mixed tissues (heart, liver, spleen, lung, kidney, intestine, stomach, brain) |
| *Pangolin pestivirus 3* | mixed tissues (heart, liver, spleen, lung, kidney, intestine, stomach, brain) |
| *Pangolin pestivirus 4* | mixed tissues (heart, liver, spleen, lung, kidney, intestine, stomach, brain) |
| *Pangolin pestivirus BIME1* | mixed tissues (lung, heart, liver, spleen, kidney, intestine, muscle) |
| *Pangolin pestivirus BIME2* | mixed tissues (lung, heart, liver, spleen, kidney, intestine, muscle) |
| *Pangolin pestivirus BIME3* | mixed tissues (lung, heart, liver, spleen, kidney, intestine, muscle) |
| *Pangolin pestivirus BIME4* | mixed tissues (lung, heart, liver, spleen, kidney, intestine) |
| *Pangolin pestivirus BIME5* | mixed tissues (lung, heart, liver, spleen, kidney, intestine, muscle) |
| *Pangolin pestivirus BIME6* | mixed tissues (lung, heart, liver, spleen, kidney, intestine, muscle) |
| *Pangolin pestivirus BIME7* | mixed tissues (lung, intestine, liver, spleen, kidney, muscle, heart) |
| *Pangolin pestivirus BIME8* | mixed tissues (lung, intestine, liver, spleen, kidney, muscle, heart) |
| *Pangolin pestivirus BIME9* | mixed tissues (lung, intestine, liver, spleen, kidney, muscle, heart) |
| *Pestivirus A* | lung |
| *Pestivirus B* | lung |
| *Pestivirus C* | lung |
| *Pestivirus D* | lung |
| *Pestivirus H* | lung |
| *Pestivirus K* | lung |
| *Pestivirus* sp. | lung |
| | intestine |
| | heart |
| | spleen |
| | muscle |

| | |
|---|---|
| | mixed tissues (throat swabs, anal swabs) |
| *Phocoena pestivirus* | lung |
| *Tunisian sheep virus* | lung |
| *Dongyang pangolin virus* | lung |
| | mixed tissues (blood, liver, spleen, lung, kidney, fecal) |
| *Guangxi tick virus* | sequence only |
| *Cucumber mosaic virus* | lung |
| *Chikungunya virus* | mixed tissues (throat swabs, anal swabs) |
| *Getah virus* | mixed tissues (throat swabs, anal swabs) |
| *Hedgehog coronavirus 1* | lung |
| *Hypsugo bat coronavirus HKU25* | lung |
| *Manis javanica HKU4-related coronavirus* | anal swabs |
| *Middle East respiratory syndrome-related coronavirus* | lung |
| *Pangolin coronavirus* | blood |
| | lung |
| | stomach |
| | lymph node |
| | scale sample |
| | heart |
| | spleen |
| | skin |
| | cerebellum |
| | muscles |
| | faeces |
| | intestinal content |
| | throat swabs |
| | anal swabs |
| | rectal swabs |
| | oral swabs |
| | mixed tissues (lung, lymph, spleen) |
| *Pangolin coronavirus HKU4* | mixed tissues (lung, heart, liver, spleen, kidney, intestine, muscle) |
| *Pipistrellus bat coronavirus HKU5* | lung |
| SARS-CoV-2-related coronavirus | lung |
| | rectal swab |
| *Tylonycteris bat coronavirus HKU4* | lung |
| *Tylonycteris pachypus bat coronavirus HKU4-related* | lung |
| *Bat coronavirus* | lung |
| *Tylonycteris robustula coronavirus 162275* | lung |
| *Sapovirus* sp. | mixed tissues (throat swabs, anal swabs, lung, heart, spleen, muscle, intestine) |

| | |
|---|---|
| *Pangolin hunnivirus* | mixed tissues (heart, liver, spleen, lung, kidney, intestine, stomach, brain) |
| *Pangolin hunnivirus BIME1* | mixed tissues (lung, heart, liver, spleen, kidney, intestine, muscle) |
| *Pangolin hunnivirus BIME2* | mixed tissues (lung, intestine, liver, spleen, kidney, muscle, heart) |
| *Pangolin hunnivirus BIME3* | sequence only |
| *Pangolin hunnivirus BIME4* | mixed tissues (lung, heart, liver, spleen, kidney, intestine, muscle) |
| *Pangolin hunnivirus BIME5* | mixed tissues (lung, heart, liver, spleen, kidney, intestine, muscle) |
| *Senecavirus* sp. | lung |
| *Pangolin shanbavirus BIME1* | mixed tissues (lung, heart, liver, spleen, kidney, intestine, muscle) |
| *Pangolin astrovirus* | anal swabs |
| *Pangolin Orthomyxoviridae* sp. | sera |
| *Pangolin orthonairovirus BIME1* | mixed tissues (lung, heart, liver, spleen, kidney, intestine, muscle) |
| *Pangolin Phasmaviridae* sp. | sera |
| *Pangolin phlebovirus BIME1* | mixed tissues (lung, intestine, liver, spleen, kidney, muscle, heart) |
| *Pangolin Phenuiviridae* sp. | sera |
| *Human orthorubulavirus 2* | mixed tissues (heart, liver, spleen, lung, kidney, intestine, stomach, brain) |
| *Mammalian orthorubulavirus 5* | lung |
| *Murine respirovirus* | lung |
| Pangolin parainfluenza 3 virus | lung |
| *Pangolin respirovirus* | lung |
| | mixed tissues (lung, intestine, liver, spleen, kidney, muscle, heart) |
| *Bovine orthopneumovirus* | lung |
| *Human orthopneumovirus* | mixed tissues (lungs, intestine, liver, kidney, spleen, muscle) |
| | mixed tissues (lung, heart, liver, spleen, kidney, intestine, muscle) |
| | lung |
| *Murine orthopneumovirus* | mixed tissues (heart, liver, spleen, lung, kidney, intestine, stomach, brain) |
| | lung |
| *Pangolin orthopneumovirus BIME1* | mixed tissues (lung, heart, liver, spleen, kidney, intestine, muscle) |
| *Canine pneumovirus* | lung |
| *Pangolin Rhabdoviridae* sp. | sera |
| *Aeromonas dhakensis* | muscle |
| *Bacillus* sp. | sequence only |
| *Lysinibacillus* sp. | sequence only |
| *Jeotgalicoccus* sp. | sequence only |
| *Staphylococcus* sp. | sequence only |
| *Paraburkholderia fungorum* | mixed tissues (cerebrum, cerebellum and fetus) |
| *Citrobacter cronae* | sequence only |
| *Escherichia coli* | faeces |
| *Klebsiella pneumoniae* | sequence only |
| *Klebsiella quasipneumoniae* | sequence only |
| *Morganella morganii* | lung |
| *Proteus mirabilis* | sequence only |

| | |
|---|---|
| *Proteus penneri* | sequence only |
| *Proteus* sp. | sequence only |
| *Serratia marcescens* | sequence only |
| *Clostridium botulinum* | faeces |
| *Myroides odoratimimus* | sequence only |
| *Enterococcus* sp. | sequence only |
| *Streptococcus dysgalactiae* | sequence only |
| *Brachybacterium* sp. | sequence only |
| *Micrococcus* sp. | sequence only |
| *Acinetobacter baumannii* | sequence only |
| *Uncharacterized Mycoplasma* | blood |
| *Candidatus* Anaplasma pangolinii | blood |
| *Ehrlichia ruminantium* | blood |
| | lung |
| | pancreas |
| | liver |
| | heart |
| | spleen |
| | intestine |
| Uncharacterized *Ehrlichia* | blood |
| | lung |
| | spleen |
| uncultured *Ehrlichia* sp. | sequence only |
| *Giardia intestinalis* | sequence only |
| *Eimeria* cf. *tenggilingi* L12_Ros | faeces |
| *Eimeria nkaka* | intestine |
| | faeces |
| *Haemosporida* sp. B LB-2015 | blood |
| | liver |
| | spleen |
| *Babesia* sp. | sequence only |
| Uncharacterized *Babesia* | blood |
| *Trypanosoma brucei* | blood |
| *Trypanosoma vivax* | blood |
| *Enterocytozoon bieneusi* | faeces |

**Supplementary Table 2. Extracting source of microbe species information**

| Species | Publication | Sequence |
|---|---|---|
| *Hukuchivirus IN93* | Yes | |
| *Catovirus CTV1* | Yes | |
| *Hokovirus HKV1* | Yes | |
| *Alphapapillomavirus 7* | Yes | |
| *Manis javanica papillomavirus 1* | Yes | Yes |
| *Manis pentadactyla papillomavirus 1* | Yes | Yes |
| *Pangolin circovirus* | Yes | Yes |
| *Cyclovirus* sp. | Yes | Yes |
| *Gemykibivirus* sp. | Yes | Yes |
| *Pangolin chaphamaparvovirus BIME1* | Yes | Yes |
| *Pangolin Copiparvovirus* | Yes | Yes |
| *Pangolin copiparvovirus BIME1* | Yes | Yes |
| *Pangolin copiparvovirus BIME2* | Yes | Yes |
| *Carnivore protoparvovirus 1* | Yes | Yes |
| *Pangolin densovirus* | Yes | Yes |
| *Parus major densovirus* | Yes | |
| *Etatorquevirus* sp. | Yes | Yes |
| *Tettorquevirus* sp. | Yes | Yes |
| *Retroviridae* sp. | Yes | Yes |
| *Pangolin picobirnavirus* | Yes | Yes |
| *Rotavirus A* | Yes | Yes |
| *Mammalian orthoreovirus* | Yes | Yes |
| *Phocid orthoreovirus 1* | Yes | |
| *Lishui pangolin virus* | Yes | Yes |
| *Pangolin Reoviridae* sp. | Yes | Yes |
| *Japanese encephalitis virus* | Yes | Yes |
| *Pangolin pestivirus* | Yes | Yes |
| *Pangolin pestivirus 1* | Yes | Yes |
| *Pangolin pestivirus 2* | Yes | Yes |
| *Pangolin pestivirus 3* | Yes | Yes |
| *Pangolin pestivirus 4* | Yes | Yes |
| *Pangolin pestivirus BIME1* | Yes | Yes |
| *Pangolin pestivirus BIME2* | Yes | Yes |
| *Pangolin pestivirus BIME3* | Yes | Yes |
| *Pangolin pestivirus BIME4* | Yes | Yes |
| *Pangolin pestivirus BIME5* | Yes | Yes |
| *Pangolin pestivirus BIME6* | Yes | Yes |
| *Pangolin pestivirus BIME7* | Yes | Yes |
| *Pangolin pestivirus BIME8* | Yes | Yes |

| | | |
|---|---|---|
| *Pangolin pestivirus BIME9* | Yes | Yes |
| *Pestivirus A* | Yes | |
| *Pestivirus B* | Yes | |
| *Pestivirus C* | Yes | |
| *Pestivirus D* | Yes | |
| *Pestivirus H* | Yes | |
| *Pestivirus K* | Yes | |
| *Pestivirus* sp. | Yes | |
| *Phocoena pestivirus* | Yes | |
| *Tunisian sheep virus* | Yes | |
| *Dongyang pangolin virus* | Yes | Yes |
| *Guangxi tick virus* | | Yes |
| *Cucumber mosaic virus* | Yes | |
| *Chikungunya virus* | Yes | Yes |
| *Getah virus* | Yes | Yes |
| *Hedgehog coronavirus 1* | Yes | |
| *Hypsugo bat coronavirus HKU25* | Yes | |
| *Manis javanica HKU4-related coronavirus* | Yes | Yes |
| *Middle East respiratory syndrome-related coronavirus* | Yes | |
| *Pangolin coronavirus* | Yes | Yes |
| *Pangolin coronavirus HKU4* | Yes | Yes |
| *Pipistrellus bat coronavirus HKU5* | Yes | |
| SARS-CoV-2-related coronavirus | Yes | |
| *Tylonycteris bat coronavirus HKU4* | Yes | |
| *Tylonycteris pachypus bat coronavirus HKU4-related* | Yes | |
| *Bat coronavirus* | Yes | |
| *Tylonycteris robustula coronavirus 162275* | Yes | |
| *Sapovirus* sp. | Yes | Yes |
| *Pangolin hunnivirus* | Yes | Yes |
| *Pangolin hunnivirus BIME1* | Yes | Yes |
| *Pangolin hunnivirus BIME2* | Yes | Yes |
| *Pangolin hunnivirus BIME3* | Yes | Yes |
| *Pangolin hunnivirus BIME4* | Yes | Yes |
| *Pangolin hunnivirus BIME5* | Yes | Yes |
| *Senecavirus* sp. | Yes | Yes |
| *Pangolin shanbavirus BIME1* | Yes | Yes |
| *Pangolin astrovirus* | Yes | Yes |
| *Pangolin Orthomyxoviridae* sp. | Yes | Yes |
| *Pangolin orthonairovirus BIME1* | Yes | Yes |
| *Pangolin Phasmaviridae* sp. | Yes | Yes |
| *Pangolin phlebovirus BIME1* | Yes | Yes |

| | | |
|---|---|---|
| *Pangolin Phenuiviridae* sp. | Yes | Yes |
| *Human orthorubulavirus 2* | Yes | Yes |
| *Mammalian orthorubulavirus 5* | Yes | Yes |
| *Murine respirovirus* | Yes | |
| Pangolin parainfluenza 3 virus | Yes | Yes |
| *Pangolin respirovirus* | Yes | Yes |
| *Bovine orthopneumovirus* | Yes | |
| *Human orthopneumovirus* | Yes | Yes |
| *Murine orthopneumovirus* | Yes | Yes |
| *Pangolin orthopneumovirus BIME1* | Yes | Yes |
| *Canine pneumovirus* | Yes | |
| *Pangolin Rhabdoviridae* sp. | Yes | Yes |
| *Aeromonas dhakensis* | Yes | |
| *Bacillus* sp. | | Yes |
| *Lysinibacillus* sp. | | Yes |
| *Jeotgalicoccus* sp. | | Yes |
| *Staphylococcus* sp. | | Yes |
| *Paraburkholderia fungorum* | Yes | Yes |
| *Citrobacter cronae* | | Yes |
| *Escherichia coli* | Yes | Yes |
| *Klebsiella pneumoniae* | | Yes |
| *Klebsiella quasipneumoniae* | | Yes |
| *Morganella morganii* | Yes | Yes |
| *Proteus mirabilis* | | Yes |
| *Proteus penneri* | | Yes |
| *Proteus* sp. | | Yes |
| *Serratia marcescens* | | Yes |
| *Clostridium botulinum* | Yes | |
| *Myroides odoratimimus* | | Yes |
| *Enterococcus* sp. | | Yes |
| *Streptococcus dysgalactiae* | | Yes |
| *Brachybacterium* sp. | | Yes |
| *Micrococcus* sp. | | Yes |
| *Acinetobacter baumannii* | | Yes |
| Uncharacterized *Mycoplasma* | Yes | |
| *Candidatus* Anaplasma pangolinii | Yes | |
| *Ehrlichia ruminantium* | Yes | |
| Uncharacterized *Ehrlichia* | Yes | |
| Uncultured *Ehrlichia* sp. | | Yes |
| *Giardia intestinalis* | | Yes |
| *Eimeria* cf. *tenggilingi* L12_Ros | Yes | Yes |

| | | |
|---|---|---|
| *Eimeria nkaka* | Yes | Yes |
| *Haemosporida* sp. B LB-2015 | Yes | Yes |
| *Babesia* sp. | | Yes |
| Uncharacterized *Babesia* | Yes | |
| *Trypanosoma brucei* | Yes | |
| *Trypanosoma vivax* | Yes | |
| *Enterocytozoon bieneusi* | Yes | |

**Supplementary Table 3. Sequence information of pangolin-associated microbes**

| Accession | Species of pangolin | Species |
|---|---|---|
| BK062771 | *Manis javanica* | *Manis javanica papillomavirus 1* |
| BK062772 | *Manis javanica* | *Manis javanica papillomavirus 1* |
| BK062773 | *Manis javanica* | *Manis javanica papillomavirus 1* |
| BK062774 | *Manis javanica* | *Manis javanica papillomavirus 1* |
| BK062775 | *Manis javanica* | *Manis javanica papillomavirus 1* |
| BK062776 | *Manis pentadactyla* | *Manis javanica papillomavirus 1* |
| BK062777 | *Manis pentadactyla* | *Manis javanica papillomavirus 1* |
| BK062778 | *Manis pentadactyla* | *Manis pentadactyla papillomavirus 1* |
| CP028829 | *Manis javanica* | *Paraburkholderia fungorum* |
| CP028830 | *Manis javanica* | *Paraburkholderia fungorum* |
| CP028831 | *Manis javanica* | *Paraburkholderia fungorum* |
| CP028832 | *Manis javanica* | *Paraburkholderia fungorum* |
| CP053615 | *Manis javanica* | *Proteus mirabilis* |
| CP053616 | *Manis javanica* | *Proteus mirabilis* |
| CP053719 | *Manis javanica* | *Proteus mirabilis* |
| CP058544 | *Manis javanica* | *Klebsiella pneumoniae* |
| CP058545 | *Manis javanica* | *Klebsiella pneumoniae* |
| CP058546 | *Manis javanica* | *Klebsiella pneumoniae* |
| CP058547 | *Manis javanica* | *Klebsiella pneumoniae* |
| CP058548 | *Manis javanica* | *Klebsiella pneumoniae* |
| CP058549 | *Manis javanica* | *Klebsiella pneumoniae* |
| CP058550 | *Manis javanica* | *Klebsiella pneumoniae* |
| CP058551 | *Manis javanica* | *Klebsiella pneumoniae* |
| CP058709 | *Manis javanica* | *Escherichia coli* |
| CP058710 | *Manis javanica* | *Escherichia coli* |
| CP058711 | *Manis javanica* | *Escherichia coli* |
| CP058712 | *Manis javanica* | *Escherichia coli* |
| CP058713 | *Manis javanica* | *Escherichia coli* |
| CP058714 | *Manis javanica* | *Escherichia coli* |
| CP058715 | *Manis javanica* | *Escherichia coli* |
| CP058716 | *Manis javanica* | *Escherichia coli* |
| CP058717 | *Manis javanica* | *Escherichia coli* |
| CP058718 | *Manis javanica* | *Escherichia coli* |
| CP058719 | *Manis javanica* | *Escherichia coli* |
| CP058720 | *Manis javanica* | *Escherichia coli* |
| CP058721 | *Manis javanica* | *Escherichia coli* |
| CP058722 | *Manis javanica* | *Escherichia coli* |
| CP058723 | *Manis javanica* | *Escherichia coli* |
| CP058724 | *Manis javanica* | *Escherichia coli* |
| CP058725 | *Manis javanica* | *Escherichia coli* |
| CP058726 | *Manis javanica* | *Escherichia coli* |
| CP058727 | *Manis javanica* | *Escherichia coli* |

| | | |
|---|---|---|
| CP058728 | *Manis javanica* | *Escherichia coli* |
| CP058729 | *Manis javanica* | *Acinetobacter baumannii* |
| CP058730 | *Manis javanica* | *Acinetobacter baumannii* |
| CP058731 | *Manis javanica* | *Acinetobacter baumannii* |
| CP059474 | *Manis javanica* | *Acinetobacter baumannii* |
| CP059475 | *Manis javanica* | *Acinetobacter baumannii* |
| CP059476 | *Manis javanica* | *Acinetobacter baumannii* |
| CP059477 | *Manis javanica* | *Morganella morganii* |
| CP059690 | *Manis javanica* | *Proteus penneri* |
| CP059691 | *Manis javanica* | *Proteus penneri* |
| CP060440 | *Manis javanica* | *Serratia marcescens* |
| CP060441 | *Manis javanica* | *Citrobacter cronae* |
| CP060442 | *Manis javanica* | *Citrobacter cronae* |
| CP060443 | *Manis javanica* | *Citrobacter cronae* |
| CP060444 | *Manis javanica* | *Citrobacter cronae* |
| CP060445 | *Manis javanica* | *Citrobacter cronae* |
| CP063874 | *Manis javanica* | *Klebsiella quasipneumoniae* |
| CP063875 | *Manis javanica* | *Klebsiella quasipneumoniae* |
| CP063876 | *Manis javanica* | *Klebsiella quasipneumoniae* |
| CP063877 | *Manis javanica* | *Klebsiella quasipneumoniae* |
| CP063878 | *Manis javanica* | *Klebsiella pneumoniae* |
| CP063879 | *Manis javanica* | *Klebsiella pneumoniae* |
| CP063880 | *Manis javanica* | *Klebsiella pneumoniae* |
| CP063945 | *Manis javanica* | *Klebsiella pneumoniae* |
| CP063946 | *Manis javanica* | *Klebsiella pneumoniae* |
| CP063947 | *Manis javanica* | *Klebsiella pneumoniae* |
| CP063948 | *Manis javanica* | *Klebsiella pneumoniae* |
| CP063949 | *Manis javanica* | *Klebsiella pneumoniae* |
| CP063992 | *Manis javanica* | *Klebsiella pneumoniae* |
| CP101666 | *Manis javanica* | *Escherichia coli* |
| CP101667 | *Manis javanica* | *Escherichia coli* |
| CP101668 | *Manis javanica* | *Escherichia coli* |
| EPI_ISL_410721 | *Manis javanica* | *Pangolin coronavirus* |
| GWHBHAL01000000 | *Manis javanica* | *Manis javanica HKU4-related coronavirus* |
| GWHBIUK01000000 | *Manis javanica* | *Manis javanica HKU4-related coronavirus* |
| GWHBIUL01000000 | *Manis javanica* | *Manis javanica HKU4-related coronavirus* |
| GWHBIUM01000000 | *Manis javanica* | *Manis javanica HKU4-related coronavirus* |
| JQ993651 | *Phataginus tricuspis* | *Eimeria nkaka* |
| JQ993677 | *Phataginus tricuspis* | *Eimeria nkaka* |
| JQ993697 | *Phataginus tricuspis* | *Eimeria nkaka* |
| JX464222 | *Manis javanica* | *Eimeria cf. tenggilingi* L12_Ros |
| JX464223 | *Manis javanica* | *Eimeria cf. tenggilingi* L12_Ros |
| KC847109 | Pangolin | *Myroides odoratimimus* |
| KC847110 | Pangolin | *Streptococcus dysgalactiae* |

| | | |
|---|---|---|
| KM672663 | *Manis crassicaudata* | *Proteus* sp. |
| KM672664 | *Manis crassicaudata* | *Proteus* sp. |
| KT367818 | *Phataginus tricuspis* | *Haemosporida* sp. B LB-2015 |
| KX168695 | *Manis javanica* | *Babesia* sp. |
| KX168696 | *Manis javanica* | *Babesia* sp. |
| MF136789 | *Manis crassicaudata* | *Bacillus* sp. |
| MF148852 | *Manis crassicaudata* | *Bacillus* sp. |
| MF148854 | *Manis crassicaudata* | *Enterococcus* sp. |
| MF149109 | *Manis crassicaudata* | *Lysinibacillus* sp. |
| MF157700 | *Manis crassicaudata* | *Staphylococcus* sp. |
| MF164038 | *Manis crassicaudata* | *Jeotgalicoccus* sp. |
| MF164151 | *Manis crassicaudata* | *Proteus* sp. |
| MF164152 | *Manis crassicaudata* | *Proteus* sp. |
| MF179522 | *Manis crassicaudata* | *Enterococcus* sp. |
| MF179523 | *Manis crassicaudata* | *Bacillus* sp. |
| MF179524 | *Manis crassicaudata* | *Brachybacterium* sp. |
| MF179525 | *Manis crassicaudata* | *Bacillus* sp. |
| MF179528 | *Manis crassicaudata* | *Micrococcus* sp. |
| MF185140 | *Manis crassicaudata* | *Proteus* sp. |
| MF185141 | *Manis crassicaudata* | *Bacillus* sp. |
| MF185142 | *Manis crassicaudata* | *Proteus* sp. |
| MF185144 | *Manis crassicaudata* | *Staphylococcus* sp. |
| MF185145 | *Manis crassicaudata* | *Bacillus* sp. |
| MG921602 | *Manis javanica* | *Mammalian orthorubulavirus 5* |
| MH362816 | Pangolin | *Mammalian orthorubulavirus 5* |
| MH457192 | *Manis javanica* | *Guangxi tick virus* |
| MH457193 | *Manis javanica* | *Guangxi tick virus* |
| MH457194 | *Manis javanica* | *Guangxi tick virus* |
| MH457195 | *Manis javanica* | *Guangxi tick virus* |
| MK636875 | *Manis javanica* | *Dongyang pangolin virus* |
| MK636876 | *Manis javanica* | *Lishui pangolin virus* |
| MK636877 | *Manis javanica* | *Lishui pangolin virus* |
| MK636878 | *Manis javanica* | *Lishui pangolin virus* |
| MK636879 | *Manis javanica* | *Lishui pangolin virus* |
| MK636880 | *Manis javanica* | *Lishui pangolin virus* |
| MK636881 | *Manis javanica* | *Lishui pangolin virus* |
| MK636882 | *Manis javanica* | *Lishui pangolin virus* |
| MK636883 | *Manis javanica* | *Lishui pangolin virus* |
| MK636884 | *Manis javanica* | *Lishui pangolin virus* |
| MK928409 | *Manis javanica* | uncultured *Ehrlichia* sp. |
| MK982529 | Pangolin | *Giardia intestinalis* |
| MK982534 | Pangolin | *Giardia intestinalis* |
| MN832850 | *Manis pentadactyla* | *Carnivore protoparvovirus 1* |
| MN900866 | *Manis pentadactyla* | *Carnivore protoparvovirus 1* |

| | | |
|---|---|---|
| MN900867 | *Manis pentadactyla* | *Carnivore protoparvovirus 1* |
| MN900868 | *Manis pentadactyla* | *Carnivore protoparvovirus 1* |
| MN900869 | *Manis pentadactyla* | *Carnivore protoparvovirus 1* |
| MT040333 | *Manis javanica* | *Pangolin coronavirus* |
| MT040334 | *Manis javanica* | *Pangolin coronavirus* |
| MT040335 | *Manis javanica* | *Pangolin coronavirus* |
| MT040336 | *Manis javanica* | *Pangolin coronavirus* |
| MT072864 | *Manis javanica* | *Pangolin coronavirus* |
| MT072865 | *Manis javanica* | *Pangolin coronavirus* |
| MT084071 | *Manis javanica* | *Pangolin coronavirus* |
| MT121216 | *Manis javanica* | *Pangolin coronavirus* |
| MT672412 | *Manis javanica* | *Pangolin coronavirus* |
| MT672413 | *Manis javanica* | *Pangolin coronavirus* |
| MT672414 | *Manis javanica* | *Pangolin coronavirus* |
| MT672415 | *Manis javanica* | *Pangolin coronavirus* |
| MT672416 | *Manis javanica* | *Pangolin coronavirus* |
| MT799521 | *Manis javanica* | *Pangolin coronavirus* |
| MT799522 | *Manis javanica* | *Pangolin coronavirus* |
| MT799523 | *Manis javanica* | *Pangolin coronavirus* |
| MT799524 | *Manis javanica* | *Pangolin coronavirus* |
| MT799525 | *Manis javanica* | *Pangolin coronavirus* |
| MT799526 | *Manis javanica* | *Pangolin coronavirus* |
| MW173323 | *Manis pentadactyla* | *Pangolin coronavirus* |
| MW173324 | *Manis javanica* | *Pangolin coronavirus* |
| MW450824 | *Manis javanica* | *Sapovirus* sp. |
| MW450825 | *Manis javanica* | *Pestivirus* sp. |
| MW505906 | *Manis javanica* | *Pangolin respirovirus* |
| MW532698 | Pangolin | *Pangolin coronavirus* |
| MZ173490 | *Manis javanica* | *Babesia* sp. |
| MZ173491 | *Manis javanica* | *Babesia* sp. |
| MZ173492 | *Manis javanica* | *Babesia* sp. |
| MZ173493 | *Manis javanica* | *Babesia* sp. |
| MZ173494 | *Manis javanica* | *Babesia* sp. |
| MZ173495 | *Manis javanica* | *Babesia* sp. |
| MZ173496 | *Manis javanica* | *Babesia* sp. |
| MZ173497 | *Manis javanica* | *Babesia* sp. |
| OK510873 | *Manis javanica* | *Pangolin coronavirus* |
| OK510874 | *Manis javanica* | *Pangolin coronavirus* |
| OK510875 | *Manis javanica* | *Pangolin coronavirus* |
| OK510876 | *Manis javanica* | *Pangolin coronavirus* |
| OK510877 | *Manis javanica* | *Pangolin coronavirus* |
| OK510878 | *Manis javanica* | *Pangolin coronavirus* |
| OK510879 | *Manis javanica* | *Pangolin coronavirus* |
| OK510880 | *Manis javanica* | *Pangolin coronavirus* |

| | | |
|---|---|---|
| OK510881 | *Manis javanica* | *Pangolin coronavirus* |
| OK510882 | *Manis javanica* | *Pangolin coronavirus* |
| OK510883 | *Manis javanica* | *Pangolin coronavirus* |
| OK510884 | *Manis javanica* | *Pangolin coronavirus* |
| OK510885 | *Manis javanica* | *Pangolin coronavirus* |
| OK510886 | *Manis javanica* | *Pangolin coronavirus* |
| OK510887 | *Manis javanica* | *Pangolin coronavirus* |
| OK510888 | *Manis javanica* | *Pangolin coronavirus* |
| OK510889 | *Manis javanica* | *Pangolin coronavirus* |
| OK510890 | *Manis javanica* | *Pangolin coronavirus* |
| OK510891 | *Manis javanica* | *Pangolin coronavirus* |
| OK510892 | *Manis javanica* | *Pangolin coronavirus* |
| OL519618 | *Manis pentadactyla* | *Gemykibivirus* sp. |
| OL519619 | *Manis pentadactyla* | *Retroviridae* sp. |
| OL519620 | *Manis pentadactyla* | *Senecavirus* sp. |
| OL519621 | *Manis javanica* | *Tettorquevirus* sp. |
| OL519622 | *Manis javanica* | *Etatorquevirus* sp. |
| OL519623 | *Manis pentadactyla* | *Cyclovirus* sp. |
| OM009282 | *Manis javanica* | *Pangolin coronavirus HKU4* |
| OM009283 | *Manis javanica* | *Human orthopneumovirus* |
| OM009284 | *Manis javanica* | *Human orthopneumovirus* |
| OM037454 | *Manis javanica* | *Pangolin orthopneumovirus BIME1* |
| OM141135 | *Manis javanica* | Pangolin parainfluenza 3 virus |
| OM141136 | *Manis javanica* | *Human orthopneumovirus* |
| OM141137 | *Manis javanica* | *Human orthopneumovirus* |
| OM141138 | *Manis javanica* | *Human orthopneumovirus* |
| OM141139 | *Manis javanica* | *Human orthopneumovirus* |
| OM141140 | *Manis javanica* | *Human orthopneumovirus* |
| OM141141 | *Manis javanica* | *Human orthopneumovirus* |
| OM141142 | *Manis javanica* | *Human orthopneumovirus* |
| OM141143 | *Manis javanica* | *Human orthopneumovirus* |
| OM256488 | *Manis javanica* | *Human orthopneumovirus* |
| OM256489 | *Manis javanica* | *Human orthopneumovirus* |
| OM256490 | *Manis javanica* | *Human orthopneumovirus* |
| OM256491 | *Manis javanica* | *Human orthopneumovirus* |
| OM256492 | *Manis javanica* | *Human orthopneumovirus* |
| OM256493 | *Manis javanica* | *Human orthopneumovirus* |
| OM256494 | *Manis javanica* | *Human orthopneumovirus* |
| OM256495 | *Manis javanica* | *Human orthopneumovirus* |
| OM256496 | *Manis javanica* | *Human orthopneumovirus* |
| OM256497 | *Manis javanica* | *Human orthopneumovirus* |
| OM256498 | *Manis javanica* | *Human orthopneumovirus* |
| OM256499 | *Manis javanica* | *Human orthopneumovirus* |
| OM416147 | Pangolin | *Japanese encephalitis virus* |

| | | |
|---|---|---|
| OM416148 | Pangolin | *Japanese encephalitis virus* |
| OM416149 | Pangolin | *Japanese encephalitis virus* |
| OM416150 | Pangolin | *Japanese encephalitis virus* |
| OM416151 | Pangolin | *Japanese encephalitis virus* |
| OM416152 | Pangolin | *Japanese encephalitis virus* |
| OM416153 | Pangolin | *Japanese encephalitis virus* |
| OM416154 | Pangolin | *Getah virus* |
| OM416155 | Pangolin | *Getah virus* |
| OM416156 | Pangolin | *Getah virus* |
| OM416157 | Pangolin | *Chikungunya virus* |
| OM416158 | Pangolin | *Chikungunya virus* |
| OM416159 | Pangolin | *Chikungunya virus* |
| OM416160 | Pangolin | *Chikungunya virus* |
| OM416161 | Pangolin | *Chikungunya virus* |
| OM416162 | Pangolin | *Chikungunya virus* |
| OM416163 | Pangolin | *Chikungunya virus* |
| OM451127 | *Manis pentadactyla* | *Pangolin pestivirus 1* |
| OM451128 | *Manis javanica* | *Pangolin pestivirus 2* |
| OM451129 | *Manis javanica* | *Pangolin pestivirus 2* |
| OM451130 | *Manis javanica* | *Pangolin pestivirus 3* |
| OM451131 | *Manis javanica* | *Pangolin pestivirus 3* |
| OM451132 | *Manis javanica* | *Pangolin pestivirus 4* |
| OM451133 | *Manis javanica* | *Pangolin pestivirus 4* |
| OM451136 | *Manis javanica* | *Human orthorubulavirus 2* |
| OM451137 | *Manis javanica* | *Human orthorubulavirus 2* |
| OM451179 | *Manis javanica* | *Pangolin hunnivirus* |
| OM451208 | *Manis javanica* | *Murine orthopneumovirus* |
| OM451214 | *Manis pentadactyla* | *Carnivore protoparvovirus 1* |
| OM480515 | *Manis javanica* | *Pangolin hunnivirus* |
| OM480522 | *Manis javanica* | *Carnivore protoparvovirus 1* |
| OM480525 | *Manis pentadactyla* | *Pangolin pestivirus 1* |
| ON024072 | *Manis javanica* | *Pangolin hunnivirus BIME1* |
| ON024073 | *Manis javanica* | *Pangolin hunnivirus BIME1* |
| ON024074 | *Manis javanica* | *Pangolin hunnivirus BIME2* |
| ON024075 | *Manis javanica* | *Pangolin hunnivirus BIME2* |
| ON024076 | *Manis javanica* | *Pangolin hunnivirus BIME4* |
| ON024077 | *Manis javanica* | *Pangolin hunnivirus BIME5* |
| ON024078 | *Manis javanica* | *Pangolin hunnivirus BIME5* |
| ON024079 | *Manis javanica* | *Pangolin shanbavirus BIME1* |
| ON024080 | *Manis javanica* | *Pangolin shanbavirus BIME1* |
| ON024081 | *Manis javanica* | *Pangolin phlebovirus BIME1* |
| ON024082 | *Manis javanica* | *Pangolin phlebovirus BIME1* |
| ON024083 | *Manis javanica* | *Pangolin phlebovirus BIME1* |
| ON024084 | *Manis javanica* | *Pangolin phlebovirus BIME1* |

| | | |
|---|---|---|
| ON024085 | *Manis javanica* | *Pangolin phlebovirus BIME1* |
| ON024086 | *Manis javanica* | *Pangolin phlebovirus BIME1* |
| ON024087 | *Manis javanica* | *Pangolin orthonairovirus BIME1* |
| ON024088 | *Manis javanica* | *Pangolin orthonairovirus BIME1* |
| ON024089 | *Manis javanica* | *Pangolin orthonairovirus BIME1* |
| ON024090 | *Manis javanica* | *Pangolin pestivirus BIME1* |
| ON024091 | *Manis javanica* | *Pangolin pestivirus BIME1* |
| ON024092 | *Manis javanica* | *Pangolin pestivirus BIME1* |
| ON024093 | *Manis javanica* | *Pangolin pestivirus BIME1* |
| ON024094 | *Manis javanica* | *Pangolin pestivirus BIME1* |
| ON024095 | *Manis javanica* | *Pangolin pestivirus BIME1* |
| ON024096 | *Manis javanica* | *Pangolin pestivirus BIME2* |
| ON024097 | *Manis javanica* | *Pangolin pestivirus BIME3* |
| ON024098 | *Manis javanica* | *Pangolin pestivirus BIME3* |
| ON024099 | *Manis javanica* | *Pangolin pestivirus BIME4* |
| ON024100 | *Manis javanica* | *Pangolin pestivirus BIME5* |
| ON024101 | *Manis javanica* | *Pangolin pestivirus BIME5* |
| ON024102 | *Manis javanica* | *Pangolin pestivirus BIME6* |
| ON024103 | *Manis javanica* | *Pangolin pestivirus BIME6* |
| ON024104 | *Manis javanica* | *Pangolin pestivirus BIME7* |
| ON024105 | *Manis javanica* | *Pangolin pestivirus BIME7* |
| ON024106 | *Manis javanica* | *Pangolin pestivirus BIME7* |
| ON024107 | *Manis javanica* | *Pangolin pestivirus BIME9* |
| ON024108 | *Manis javanica* | *Pangolin pestivirus BIME9* |
| ON024109 | *Manis javanica* | *Pangolin pestivirus BIME9* |
| ON024110 | *Manis javanica* | *Pangolin pestivirus BIME9* |
| ON024111 | *Manis javanica* | *Pangolin pestivirus BIME9* |
| ON024112 | *Manis javanica* | *Pangolin pestivirus BIME9* |
| ON024113 | *Manis javanica* | *Pangolin pestivirus BIME9* |
| ON024114 | *Manis javanica* | *Pangolin pestivirus BIME9* |
| ON024115 | *Manis javanica* | *Pangolin pestivirus BIME9* |
| ON024116 | *Manis javanica* | *Pangolin chaphamaparvovirus BIME1* |
| ON024117 | *Manis javanica* | *Pangolin chaphamaparvovirus BIME1* |
| ON024118 | *Manis javanica* | *Carnivore protoparvovirus 1* |
| ON024119 | *Manis javanica* | *Carnivore protoparvovirus 1* |
| ON024120 | *Manis javanica* | *Carnivore protoparvovirus 1* |
| ON024121 | *Manis javanica* | *Carnivore protoparvovirus 1* |
| ON024122 | *Manis javanica* | *Carnivore protoparvovirus 1* |
| ON024123 | *Manis javanica* | *Carnivore protoparvovirus 1* |
| ON024124 | *Manis javanica* | *Carnivore protoparvovirus 1* |
| ON024125 | *Manis javanica* | *Carnivore protoparvovirus 1* |
| ON024126 | *Manis javanica* | *Carnivore protoparvovirus 1* |
| ON024127 | *Manis javanica* | *Carnivore protoparvovirus 1* |
| ON024128 | *Manis javanica* | *Carnivore protoparvovirus 1* |

| | | |
|---|---|---|
| ON024129 | *Manis javanica* | *Carnivore protoparvovirus 1* |
| ON024130 | *Manis javanica* | *Carnivore protoparvovirus 1* |
| ON024131 | *Manis javanica* | *Carnivore protoparvovirus 1* |
| ON024132 | *Manis javanica* | *Carnivore protoparvovirus 1* |
| ON024133 | *Manis javanica* | *Carnivore protoparvovirus 1* |
| ON024134 | *Manis javanica* | *Carnivore protoparvovirus 1* |
| ON024135 | *Manis javanica* | *Carnivore protoparvovirus 1* |
| ON024136 | *Manis javanica* | *Carnivore protoparvovirus 1* |
| ON024137 | *Manis javanica* | *Carnivore protoparvovirus 1* |
| ON024138 | *Manis javanica* | *Carnivore protoparvovirus 1* |
| ON024139 | *Manis javanica* | *Carnivore protoparvovirus 1* |
| ON024140 | *Manis javanica* | *Carnivore protoparvovirus 1* |
| ON059801 | *Manis javanica* | *Lishui pangolin virus* |
| ON059802 | *Manis javanica* | *Pangolin pestivirus BIME1* |
| ON059803 | *Manis javanica* | *Pangolin pestivirus BIME3* |
| ON059804 | *Manis javanica* | *Pangolin pestivirus BIME3* |
| ON059805 | *Manis javanica* | *Pangolin pestivirus BIME3* |
| ON059806 | *Manis javanica* | *Pangolin pestivirus BIME8* |
| ON059807 | *Manis javanica* | *Pangolin pestivirus BIME9* |
| ON059808 | *Manis javanica* | *Pangolin pestivirus BIME9* |
| ON059809 | *Manis javanica* | *Pangolin pestivirus BIME9* |
| ON059810 | *Manis javanica* | *Pangolin pestivirus BIME9* |
| ON059811 | *Manis javanica* | *Pangolin pestivirus BIME9* |
| ON059812 | *Manis javanica* | *Pangolin pestivirus BIME9* |
| ON059813 | *Manis javanica* | *Pangolin pestivirus BIME9* |
| ON059814 | *Manis javanica* | *Pangolin pestivirus BIME9* |
| ON059815 | *Manis javanica* | *Pangolin pestivirus BIME9* |
| ON059816 | *Manis javanica* | *Pangolin respirovirus* |
| ON059817 | *Manis javanica* | *Pangolin respirovirus* |
| ON059818 | *Manis javanica* | *Pangolin respirovirus* |
| ON059819 | *Manis javanica* | *Pangolin respirovirus* |
| ON059820 | *Manis javanica* | *Pangolin hunnivirus BIME2* |
| ON059821 | *Manis javanica* | *Pangolin hunnivirus BIME3* |
| ON059822 | *Manis javanica* | *Pangolin hunnivirus BIME4* |
| ON059823 | *Manis javanica* | *Pangolin copiparvovirus BIME1* |
| ON059824 | *Manis javanica* | *Pangolin copiparvovirus BIME1* |
| ON059825 | *Manis javanica* | *Pangolin copiparvovirus BIME2* |
| ON059826 | *Manis javanica* | *Pangolin copiparvovirus BIME2* |
| ON059827 | *Manis javanica* | *Pangolin copiparvovirus BIME2* |
| ON059828 | *Manis javanica* | *Pangolin copiparvovirus BIME2* |
| ON059829 | *Manis javanica* | *Pangolin copiparvovirus BIME1* |
| ON059830 | *Manis javanica* | *Pangolin copiparvovirus BIME1* |
| ON059831 | *Manis javanica* | *Pangolin copiparvovirus BIME1* |
| ON059832 | *Manis javanica* | *Pangolin copiparvovirus BIME1* |

| | | |
|---|---|---|
| ON059833 | *Manis javanica* | *Pangolin copiparvovirus BIME1* |
| ON059834 | *Manis javanica* | *Pangolin copiparvovirus BIME1* |
| ON059835 | *Manis javanica* | *Pangolin copiparvovirus BIME2* |
| ON059836 | *Manis javanica* | *Pangolin copiparvovirus BIME2* |
| ON059837 | *Manis javanica* | *Pangolin copiparvovirus BIME2* |
| ON059838 | *Manis javanica* | *Rotavirus A* |
| ON059839 | *Manis javanica* | *Rotavirus A* |
| ON059840 | *Manis javanica* | *Rotavirus A* |
| ON059841 | *Manis javanica* | *Rotavirus A* |
| ON059842 | *Manis javanica* | *Rotavirus A* |
| ON059843 | *Manis javanica* | *Rotavirus A* |
| ON059844 | *Manis javanica* | *Rotavirus A* |
| ON059845 | *Manis javanica* | *Rotavirus A* |
| ON059846 | *Manis javanica* | *Rotavirus A* |
| ON059847 | *Manis javanica* | *Rotavirus A* |
| ON059848 | *Manis javanica* | *Rotavirus A* |
| ON059849 | *Manis javanica* | *Rotavirus A* |
| ON059850 | *Manis javanica* | *Rotavirus A* |
| ON059851 | *Manis javanica* | *Rotavirus A* |
| ON059852 | *Manis javanica* | *Rotavirus A* |
| ON059853 | *Manis javanica* | *Rotavirus A* |
| ON059854 | *Manis javanica* | *Rotavirus A* |
| ON059855 | *Manis javanica* | *Rotavirus A* |
| ON059856 | *Manis javanica* | *Rotavirus A* |
| ON059857 | *Manis javanica* | *Rotavirus A* |
| ON059858 | *Manis javanica* | *Rotavirus A* |
| ON059859 | *Manis javanica* | *Rotavirus A* |
| ON059860 | *Manis javanica* | *Rotavirus A* |
| ON059861 | *Manis javanica* | *Rotavirus A* |
| ON059862 | *Manis javanica* | *Rotavirus A* |
| ON059863 | *Manis javanica* | *Rotavirus A* |
| ON059864 | *Manis javanica* | *Rotavirus A* |
| ON059865 | *Manis javanica* | *Rotavirus A* |
| ON059866 | *Manis javanica* | *Rotavirus A* |
| ON059867 | *Manis javanica* | *Rotavirus A* |
| ON059868 | *Manis javanica* | *Rotavirus A* |
| ON059869 | *Manis javanica* | *Rotavirus A* |
| ON059870 | *Manis javanica* | *Rotavirus A* |
| ON059871 | *Manis javanica* | *Rotavirus A* |
| ON059872 | *Manis javanica* | *Rotavirus A* |
| ON059873 | *Manis javanica* | *Rotavirus A* |
| ON059874 | *Manis javanica* | *Rotavirus A* |
| ON059875 | *Manis javanica* | *Rotavirus A* |
| ON059876 | *Manis javanica* | *Rotavirus A* |

| | | |
|---|---|---|
| ON059877 | *Manis javanica* | *Rotavirus A* |
| ON059878 | *Manis javanica* | *Rotavirus A* |
| ON059879 | *Manis javanica* | *Rotavirus A* |
| ON059880 | *Manis javanica* | *Rotavirus A* |
| ON059881 | *Manis javanica* | *Rotavirus A* |
| ON059882 | *Manis javanica* | *Rotavirus A* |
| ON059883 | *Manis javanica* | *Rotavirus A* |
| ON059884 | *Manis javanica* | *Rotavirus A* |
| ON059885 | *Manis javanica* | *Rotavirus A* |
| ON059886 | *Manis javanica* | *Rotavirus A* |
| ON059887 | *Manis javanica* | *Rotavirus A* |
| ON059888 | *Manis javanica* | *Rotavirus A* |
| ON059889 | *Manis javanica* | *Rotavirus A* |
| ON059890 | *Manis javanica* | *Rotavirus A* |
| ON059891 | *Manis javanica* | *Rotavirus A* |
| ON059892 | *Manis javanica* | *Rotavirus A* |
| ON059893 | *Manis javanica* | *Rotavirus A* |
| ON059894 | *Manis javanica* | *Rotavirus A* |
| ON059895 | *Manis javanica* | *Rotavirus A* |
| ON059896 | *Manis javanica* | *Rotavirus A* |
| ON059897 | *Manis javanica* | *Rotavirus A* |
| ON059898 | *Manis javanica* | *Rotavirus A* |
| ON059899 | *Manis javanica* | *Rotavirus A* |
| ON059900 | *Manis javanica* | *Rotavirus A* |
| ON059901 | *Manis javanica* | *Rotavirus A* |
| ON059902 | *Manis javanica* | *Rotavirus A* |
| ON059903 | *Manis javanica* | *Rotavirus A* |
| ON059904 | *Manis javanica* | *Rotavirus A* |
| ON059905 | *Manis javanica* | *Rotavirus A* |
| ON059906 | *Manis javanica* | *Rotavirus A* |
| ON059907 | *Manis javanica* | *Rotavirus A* |
| ON059908 | *Manis javanica* | *Mammalian orthoreovirus* |
| ON059909 | *Manis javanica* | *Mammalian orthoreovirus* |
| ON166559 | *Manis javanica* | *Mammalian orthoreovirus* |
| ON166560 | *Manis javanica* | *Mammalian orthoreovirus* |
| ON166561 | *Manis javanica* | *Mammalian orthoreovirus* |
| ON166562 | *Manis javanica* | *Mammalian orthoreovirus* |
| ON166563 | *Manis javanica* | *Mammalian orthoreovirus* |
| ON843279 | *Manis javanica* | *Pangolin pestivirus* |
| OP208805 | *Manis pentadactyla* | *Carnivore protoparvovirus 1* |
| OP208806 | *Manis pentadactyla* | *Carnivore protoparvovirus 1* |
| OP474153 | *Manis javanica* | *Pangolin densovirus* |
| OP474154 | *Manis javanica* | *Pangolin Copiparvovirus* |
| OP474155 | *Manis javanica* | *Pangolin circovirus* |

| | | |
|---|---|---|
| OP474156 | *Manis javanica* | *Pangolin densovirus* |
| OP474157 | *Manis javanica* | *Pangolin circovirus* |
| OP474158 | *Manis javanica* | *Pangolin Phasmaviridae* sp. |
| OP474159 | *Manis javanica* | *Pangolin Rhabdoviridae* sp. |
| OP474160 | *Manis javanica* | *Pangolin Orthomyxoviridae* sp. |
| OP474161 | *Manis javanica* | *Pangolin pestivirus* |
| OP474162 | *Manis javanica* | *Pangolin circovirus* |
| OP474163 | *Manis javanica* | *Pangolin circovirus* |
| OP474164 | *Manis javanica* | *Pangolin circovirus* |
| OP474165 | *Manis javanica* | *Pangolin circovirus* |
| OP474166 | *Manis javanica* | *Pangolin circovirus* |
| OP474167 | *Manis javanica* | *Pangolin picobirnavirus* |
| OP474168 | *Manis javanica* | *Pangolin astrovirus* |
| OP474169 | *Manis javanica* | *Pangolin Phenuiviridae* sp. |
| OP474170 | *Manis javanica* | *Pangolin Copiparvovirus* |
| OP474171 | *Manis javanica* | *Pangolin circovirus* |
| OP474172 | *Manis javanica* | *Pangolin circovirus* |
| OP474173 | *Manis javanica* | *Pangolin Reoviridae* sp. |
| OP474174 | *Manis javanica* | *Pangolin pestivirus* |
| OP474175 | *Manis javanica* | *Pangolin pestivirus* |
| OP474176 | *Manis javanica* | *Pangolin densovirus* |
| OP474177 | *Manis javanica* | *Pangolin densovirus* |
| OP474178 | *Manis javanica* | *Pangolin Reoviridae* sp. |

**Supplementary Fig. 7. Meta-analysis of the prevalence of each family of pangolin-associated microbes.**

The heterogeneity of combined studies was quantified by $I^2$ statistic. The fixed effect model would be applied if $I^2 > 50\%$; Otherwise, the random effect model would be applied. Two-sided *P* values were provided in the plots.

### *Papillomaviridae*

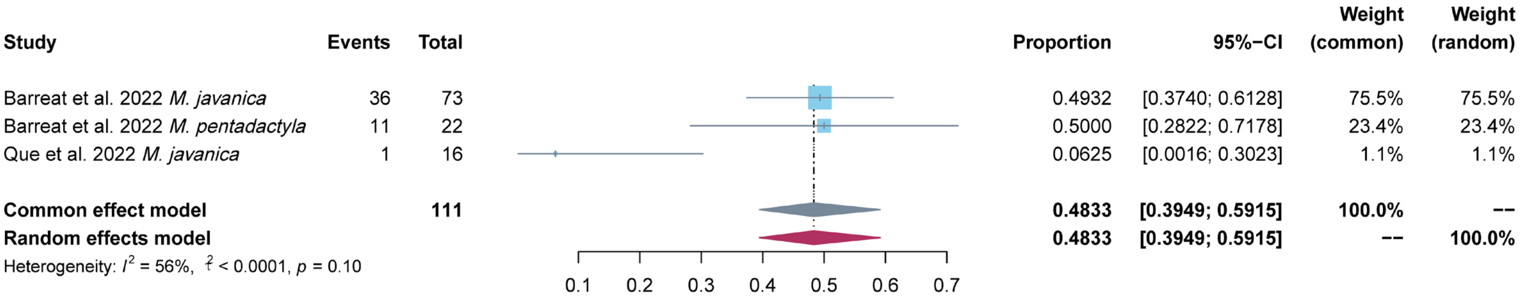


### *Parvoviridae*

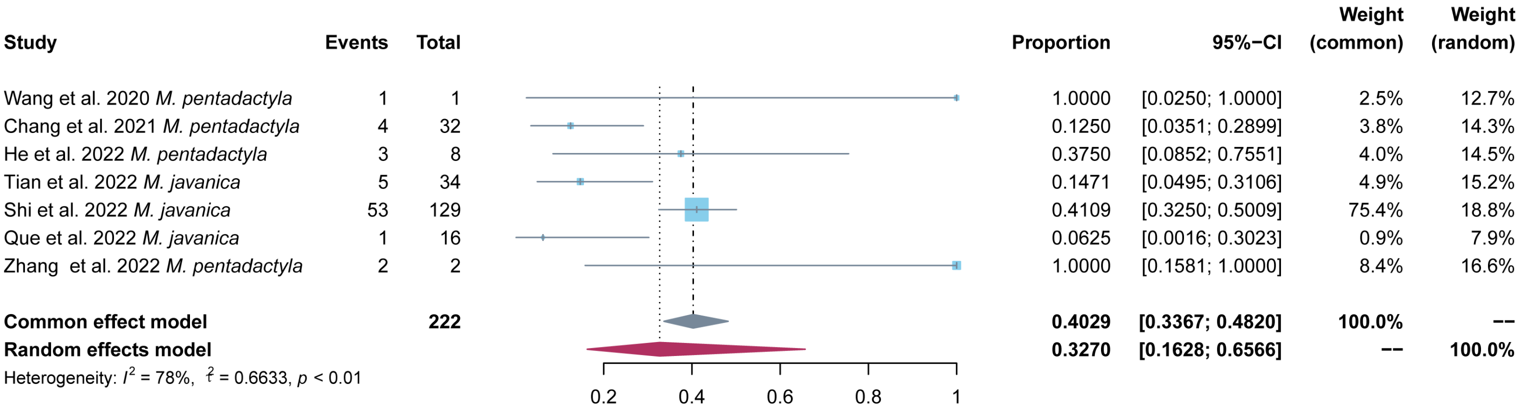


### *Spinareoviridae*

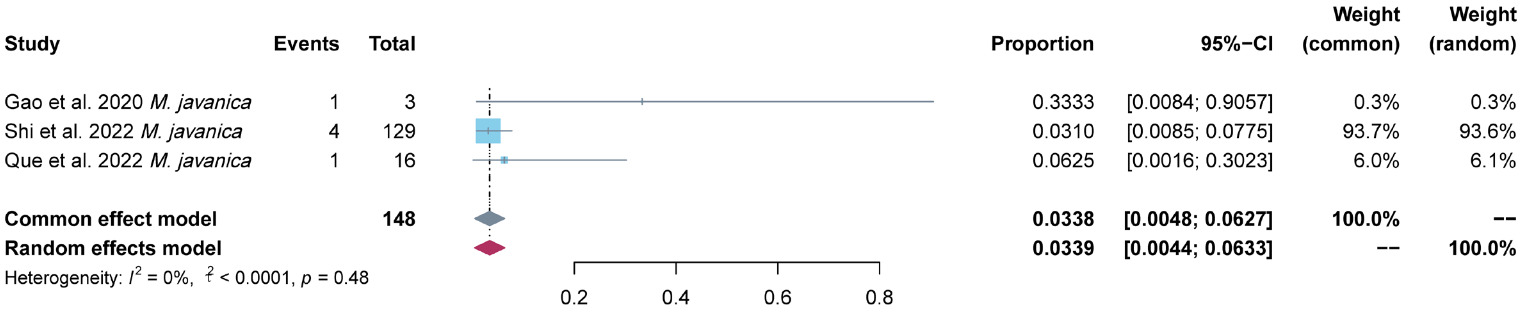


### *Picornaviridae*

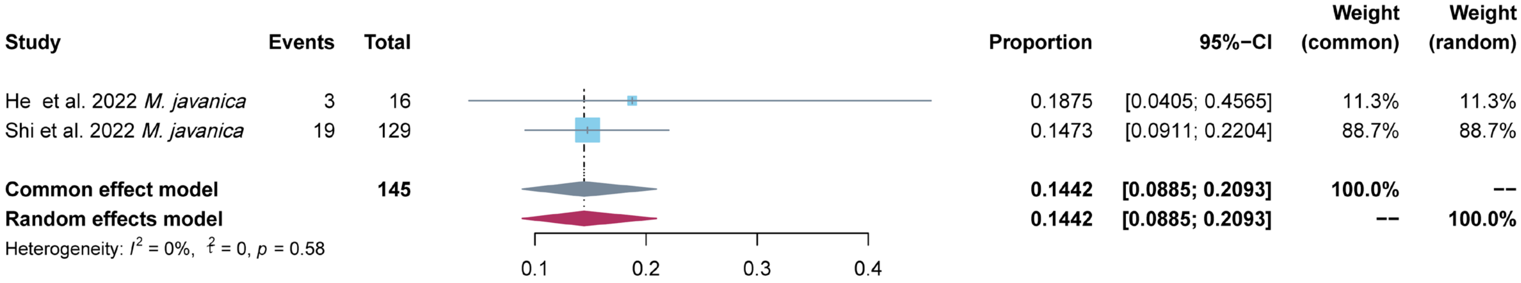

*Flaviviridae*

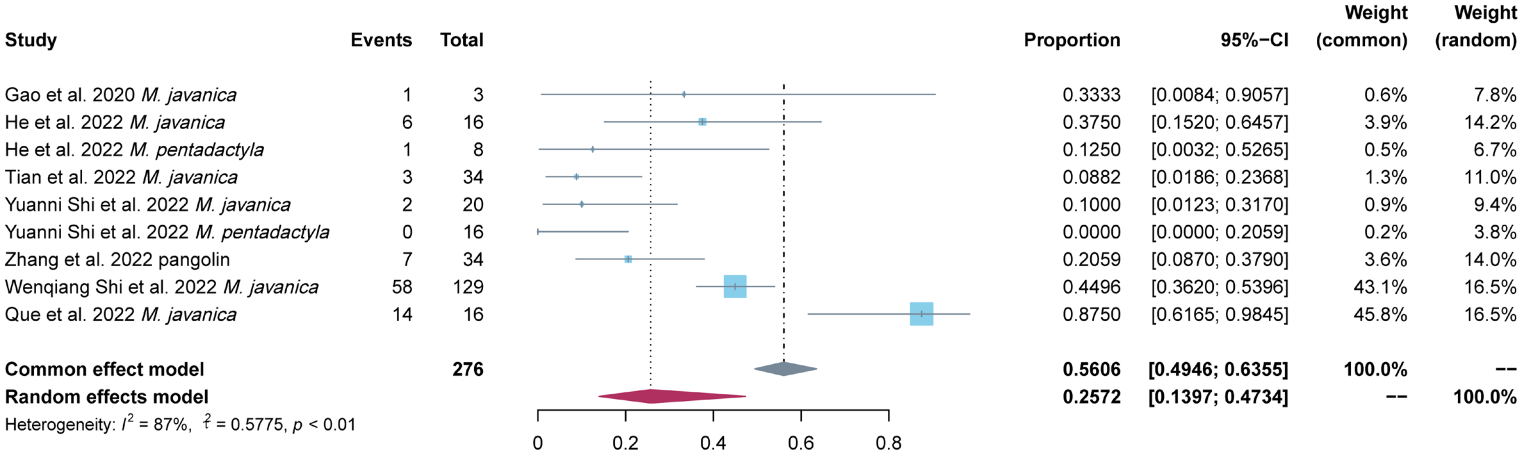


*Coronaviridae*

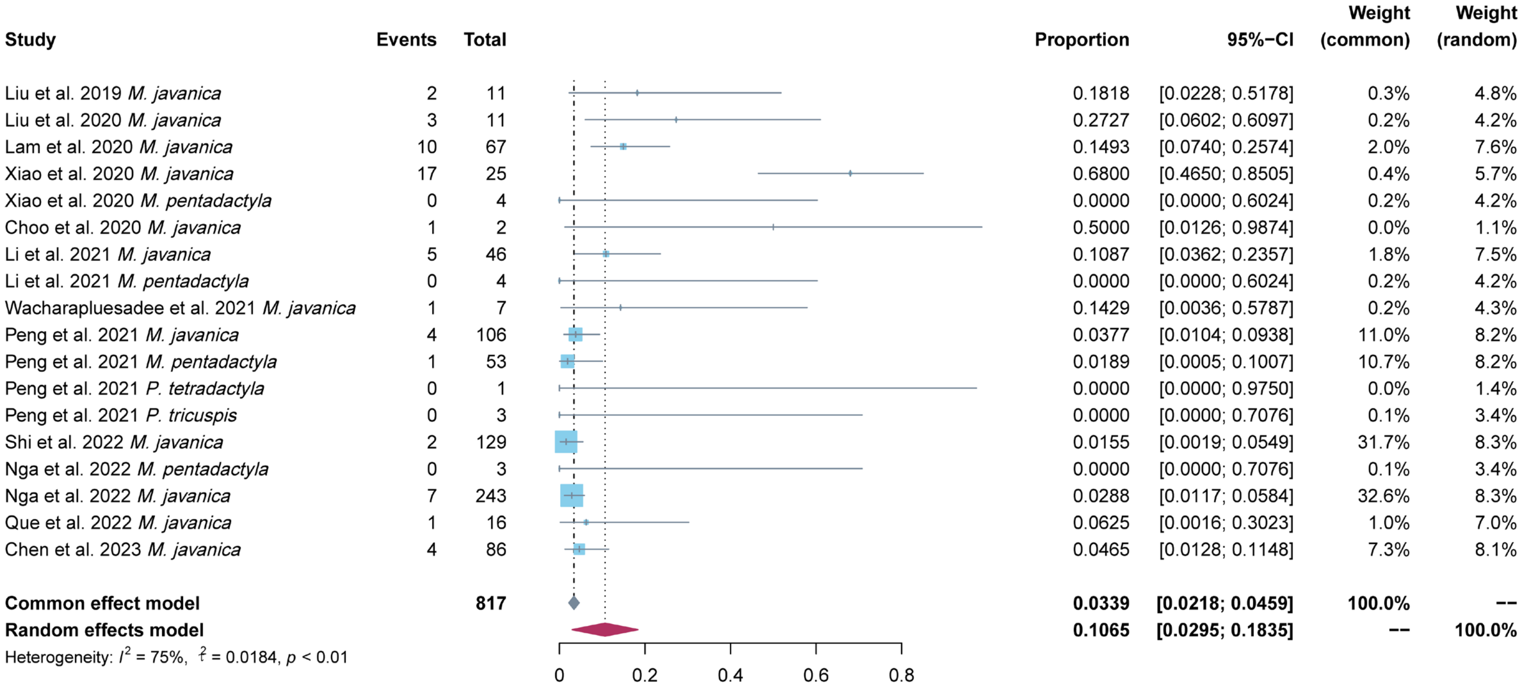


*Paramyxoviridae*

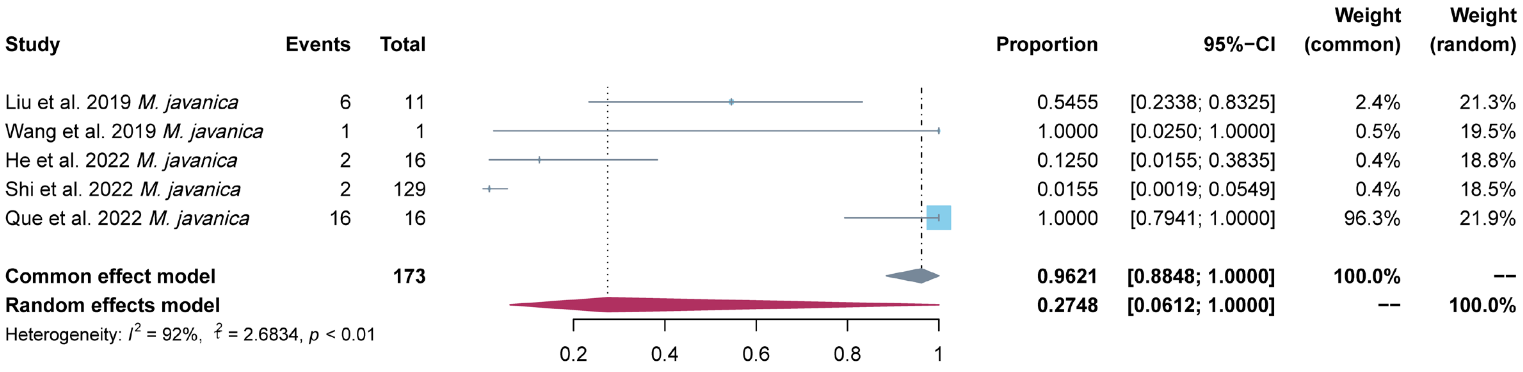

### *Pneumoviridae*

| Study | Events | Total | Proportion | 95%−CI | Weight (common) | Weight (random) |
|---|---|---|---|---|---|---|
| He et al. 2022 *M. javanica* | 1 | 16 | 0.0625 | [0.0016; 0.3023] | 8.5% | 24.6% |
| Ye et al. 2022 *M. javanica* | 12 | 30 | 0.4000 | [0.2266; 0.5940] | 15.8% | 25.1% |
| Shi et al. 2022 *M. javanica* | 7 | 129 | 0.0543 | [0.0221; 0.1086] | 67.1% | 25.7% |
| Que et al. 2022 *M. javanica* | 16 | 16 | 1.0000 | [0.7941; 1.0000] | 8.5% | 24.6% |
| **Common effect model** | | **191** | **0.1534** | **[0.1025; 0.2113]** | **100.0%** | **--** |
| **Random effects model** | | | **0.3754** | **[0.0019; 0.8869]** | **--** | **100.0%** |

Heterogeneity: $I^2 = 97\%$, $\tau^2 = 0.2943$, $p < 0.01$

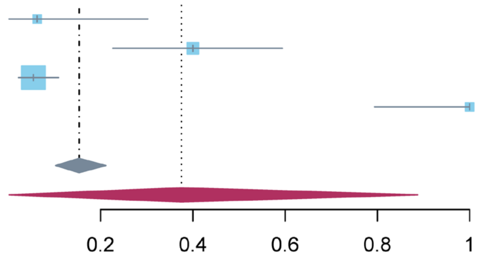


### *Phenuiviridae*

| Study | Events | Total | Proportion | 95%−CI | Weight (common) | Weight (random) |
|---|---|---|---|---|---|---|
| Tian et al. 2022 *M. javanica* | 1 | 34 | 0.0294 | [0.0007; 0.1533] | 21.0% | 21.0% |
| Shi et al. 2022 *M. javanica* | 4 | 129 | 0.0310 | [0.0085; 0.0775] | 79.0% | 79.0% |
| **Common effect model** | | **163** | **0.0279** | **[0.0056; 0.0621]** | **100.0%** | **--** |
| **Random effects model** | | | **0.0279** | **[0.0056; 0.0621]** | **--** | **100.0%** |

Heterogeneity: $I^2 = 0\%$, $\tau^2 = 0$, $p = 0.84$

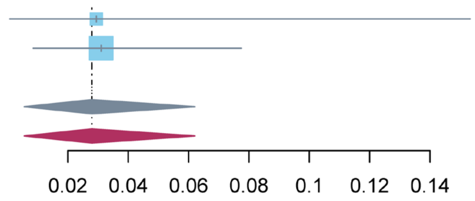


### *Morganellaceae*

| Study | Events | Total | Proportion | 95%−CI | Weight (common) | Weight (random) |
|---|---|---|---|---|---|---|
| Tao et al. 2016 *M. javanica* | 1 | 1 | 1.0000 | [0.0250; 1.0000] | 50.0% | 50.0% |
| Tao et al. 2016 *M. pentadactyla* | 1 | 1 | 1.0000 | [0.0250; 1.0000] | 50.0% | 50.0% |
| **Common effect model** | | **2** | **1.0000** | **[0.2126; 1.0000]** | **100.0%** | **--** |
| **Random effects model** | | | **1.0000** | **[0.2126; 1.0000]** | **--** | **100.0%** |

Heterogeneity: $I^2 = 0\%$, $\tau^2 = 0$, $p = 1.00$

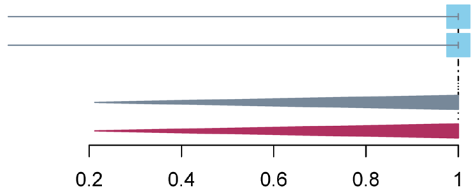


### *Anaplasmataceae*

| Study | Events | Total | Proportion | 95%−CI | Weight (common) | Weight (random) |
|---|---|---|---|---|---|---|
| Zhai et al. 2021 *M. javanica* | 10 | 21 | 0.4762 | [0.2571; 0.7022] | 54.4% | 54.4% |
| Koh et al. 2016 *M. javanica* | 8 | 15 | 0.5333 | [0.2659; 0.7873] | 39.2% | 39.2% |
| Choo et al. 2020 *M. javanica* | 1 | 2 | 0.5000 | [0.0126; 0.9874] | 6.3% | 6.3% |
| **Common effect model** | | **38** | **0.4999** | **[0.3180; 0.6817]** | **100.0%** | **--** |
| **Random effects model** | | | **0.4999** | **[0.3180; 0.6817]** | **--** | **100.0%** |

Heterogeneity: $I^2 = 0\%$, $\tau^2 = 0$, $p = 0.95$

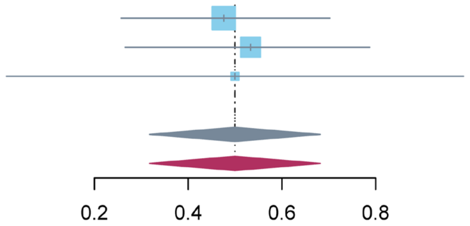

## *Eimeriidae*

| Study | Events | Total | Proportion | 95%−CI | Weight (common) | Weight (random) |
|---|---|---|---|---|---|---|
| James et al. 1976 *M. javanica* | 1 | 1 | 1.0000 | [0.0250; 1.0000] | 17.6% | 25.5% |
| Miloslav et al. 2013 *M. javanica* | 1 | 5 | 0.2000 | [0.0051; 0.7164] | 64.7% | 48.9% |
| Miloslav et al. 2013 *P. tricuspis* | 1 | 1 | 1.0000 | [0.0250; 1.0000] | 17.6% | 25.5% |
| **Common effect model** | | **7** | **0.4460** | **[0.0207; 0.9219]** | **100.0%** | **--** |
| **Random effects model** | | | **0.6090** | **[0.0000; 1.0000]** | **--** | **100.0%** |

Heterogeneity: $I^2 = 41\%$, $\tau^2 = 0.0869$, $p = 0.18$

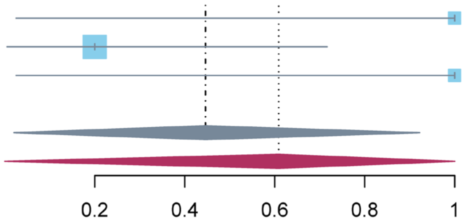


## *Trypanosomatidae*

| Study | Events | Total | Proportion | 95%−CI | Weight (common) | Weight (random) |
|---|---|---|---|---|---|---|
| Njiokou F et al. 2004 *P. tricuspis* | 5 | 20 | 0.2500 | [0.0866; 0.4910] | 14.8% | 39.4% |
| Njiokou F et al. 2004 *P. tetradactyla* | 2 | 34 | 0.0588 | [0.0072; 0.1968] | 85.2% | 60.6% |
| **Common effect model** | | **54** | **0.0871** | **[0.0141; 0.1601]** | **100.0%** | **--** |
| **Random effects model** | | | **0.1342** | **[0.0000; 0.3172]** | **--** | **100.0%** |

Heterogeneity: $I^2 = 70\%$, $\tau^2 = 0.0128$, $p = 0.07$

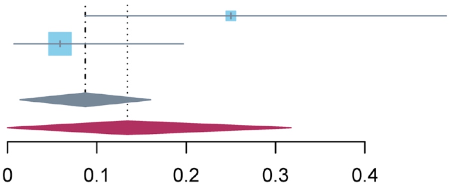

**Supplementary Table 4. Positive rate of pangolin-associated microbes**

| | Positive rate | 95% Confidence interval | Number of studies combined |
|---|---|---|---|
| **Virus** | | | |
| *Hukuchivirus IN93* | 0.1250 | NA | 1 |
| *Catovirus CTV1* | 0.0625 | NA | 1 |
| *Hokovirus HKV1* | 0.1250 | NA | 1 |
| *Alphapapillomavirus 7* | 0.0625 | NA | 1 |
| *Manis javanica papillomavirus 1* | 0.4947 | 0.3925～0.5970 | 2 |
| *Manis pentadactyla papillomavirus 1* | NA | NA | 1 |
| *Pangolin circovirus* | 0.2941 | NA | 1 |
| *Cyclovirus* sp. | NA | NA | 1 |
| *Gemykibivirus* sp. | NA | NA | 1 |
| *Pangolin chaphamaparvovirus BIME1* | 0.0233 | NA | 1 |
| *Pangolin copiparvovirus* | 0.0588 | NA | 1 |
| *Pangolin copiparvovirus BIME1* | 0.1705 | NA | 1 |
| *Pangolin copiparvovirus BIME2* | 0.1395 | NA | 1 |
| *Carnivore protoparvovirus 1* | 0.3370 | 0.0456～0.6969 | 5 |
| *Pangolin densovirus* | 0.0882 | NA | 1 |
| *Parus major densovirus* | 0.0625 | NA | 1 |
| *Etatorquevirus* sp. | 0.0400 | NA | 1 |
| *Tettorquevirus* sp. | 0.0400 | NA | 1 |
| *Retroviridae* sp. | NA | NA | 1 |
| *Pangolin picobirnavirus* | 0.0294 | NA | 1 |
| *Rotavirus A* | 0.0620 | NA | 1 |
| *Mammalian orthoreovirus* | 0.0078 | NA | 1 |
| *Phocid orthoreovirus 1* | 0.0625 | NA | 1 |
| *Lishui pangolin virus* | 0.0240 | 0.0000～0.0500 | 2 |
| *Pangolin Reoviridae* sp. | 0.0294 | NA | 1 |
| *Japanese encephalitis virus* | 0.2059 | NA | 1 |
| *Pangolin pestivirus* | 0.0843 | 0.0380~0.1874 | 3 |
| *Pangolin pestivirus 1* | 0.1250 | NA | 1 |
| *Pangolin pestivirus 2* | 0.1250 | NA | 1 |
| *Pangolin pestivirus 3* | 0.1875 | NA | 1 |
| *Pangolin pestivirus 4* | 0.1875 | NA | 1 |
| *Pangolin pestivirus BIME1* | 0.1163 | NA | 1 |
| *Pangolin pestivirus BIME2* | 0.0233 | NA | 1 |
| *Pangolin pestivirus BIME3* | 0.0620 | NA | 1 |
| *Pangolin pestivirus BIME4* | 0.0078 | NA | 1 |
| *Pangolin pestivirus BIME5* | 0.0310 | NA | 1 |
| *Pangolin pestivirus BIME6* | 0.0310 | NA | 1 |
| *Pangolin pestivirus BIME7* | 0.0155 | NA | 1 |
| *Pangolin pestivirus BIME8* | 0.0155 | NA | 1 |
| *Pangolin pestivirus BIME9* | 0.2403 | NA | 1 |

| | | | |
|---|---|---|---|
| *Pestivirus A* | 0.8125 | NA | 1 |
| *Pestivirus B* | 0.5000 | NA | 1 |
| *Pestivirus C* | 0.4375 | NA | 1 |
| *Pestivirus D* | 0.2500 | NA | 1 |
| *Pestivirus H* | 0.1250 | NA | 1 |
| *Pestivirus K* | 0.0625 | NA | 1 |
| *Pestivirus* sp. | NA | NA | 1 |
| *Phocoena pestivirus* | 0.2500 | NA | 1 |
| *Tunisian sheep virus* | 0.0625 | NA | 1 |
| *Dongyang pangolin virus* | 0.7073 | 0.4535～0.9165 | 2 |
| *Cucumber mosaic virus* | 0.0625 | NA | 1 |
| *Chikungunya virus* | 0.2059 | NA | 1 |
| *Getah virus* | 0.0882 | NA | 1 |
| *Hedgehog coronavirus 1* | 0.0625 | NA | 1 |
| *Hypsugo bat coronavirus HKU25* | 0.0625 | NA | 1 |
| *Manis javanica HKU4-related coronavirus* | 0.0465 | NA | 1 |
| *Middle East respiratory syndrome-related coronavirus* | 0.0625 | NA | 1 |
| *Pangolin coronavirus* | 0.1249 | 0.0108～0.2390 | 13 |
| *Pangolin coronavirus HKU4* | 0.1550 | NA | 1 |
| *Pipistrellus bat coronavirus HKU5* | 0.0625 | NA | 1 |
| *SARS-CoV-2-related coronavirus* | 0.1650 | 0.0158～0.3895 | 2 |
| *Tylonycteris bat coronavirus HKU4* | 0.0625 | NA | 1 |
| *Tylonycteris pachypus bat coronavirus HKU4-related* | 0.0625 | NA | 1 |
| *Bat coronavirus* | 0.0625 | NA | 1 |
| *Tylonycteris robustula coronavirus 162275* | 0.0625 | NA | 1 |
| *Sapovirus* sp. | NA | NA | 1 |
| *Pangolin hunnivirus* | 0.1875 | NA | 1 |
| *Pangolin hunnivirus BIME1* | 0.0388 | NA | 1 |
| *Pangolin hunnivirus BIME2* | 0.0155 | NA | 1 |
| *Pangolin hunnivirus BIME3* | NA | NA | 1 |
| *Pangolin hunnivirus BIME4* | 0.0155 | NA | 1 |
| *Pangolin hunnivirus BIME5* | 0.0310 | NA | 1 |
| *Senecavirus* sp. | NA | NA | 1 |
| *Pangolin shanbavirus BIME1* | 0.0620 | NA | 1 |
| *Pangolin astrovirus* | 0.0294 | NA | 1 |
| *Pangolin Orthomyxoviridae* sp. | 0.0294 | NA | 1 |
| *Pangolin orthonairovirus BIME1* | 0.0233 | NA | 1 |
| *Pangolin Phasmaviridae* sp. | 0.0294 | NA | 1 |
| *Pangolin phlebovirus BIME1* | 0.0310 | NA | 1 |
| *Pangolin Phenuiviridae* sp. | 0.0294 | NA | 1 |
| *Human orthorubulavirus 2* | 0.1250 | NA | 1 |
| *Mammalian orthorubulavirus 5* | 1.0000 | NA | 1 |
| *Murine respirovirus* | 0.5455 | NA | 1 |
| Pangolin parainfluenza 3 virus | 1.0000 | NA | 1 |

| | | | |
|---|---|---|---|
| *Pangolin respirovirus* | 0.0155 | NA | 1 |
| *Bovine orthopneumovirus* | 0.0625 | NA | 1 |
| *Human orthopneumovirus* | 0.5003 | 0.0001～0.9999 | 3 |
| *Murine orthopneumovirus* | 0.1978 | 0.0000～0.5615 | 2 |
| *Pangolin orthopneumovirus BIME1* | 0.0078 | NA | 1 |
| *Canine pneumovirus* | 0.3125 | NA | 1 |
| *Pangolin Rhabdoviridae sp.* | 0.0294 | NA | 1 |
| **Bacterium** | | | |
| *Aeromonas dhakensis* | 1.0000 | NA | 1 |
| *Paraburkholderia fungorum* | NA | NA | 1 |
| *Escherichia coli* | 1.0000 | NA | 1 |
| *Morganella morganii* | 1.0000 | 0.2126～1.0000 | 2 |
| *Clostridium botulinum* | NA | NA | 1 |
| Uncharacterized *Mycoplasma* | 0.3750 | NA | 1 |
| *Candidatus* Anaplasma pangolinii | 0.5333 | NA | 1 |
| *Ehrlichia ruminantium* | 0.5000 | NA | 1 |
| Uncharacterized *Ehrlichia* | 0.4762 | NA | 1 |
| **Protist** | | | |
| *Eimeria cf. Tenggilingi* L12_Ros | 0.4568 | 0.0000～1.0000 | 2 |
| *Eimeria nkaka* | 1.0000 | NA | 1 |
| *Haemosporida* sp. B LB-2015 | 0.0263 | NA | 1 |
| Uncharacterized *Babesia* | 0.4141 | NA | 1 |
| *Trypanosoma brucei* | 0.0545 | 0.0044～0.1387 | 2 |
| *Trypanosoma vivax* | 0.1338 | 0.0036～0.3665 | 2 |
| *Enterocytozoon bieneusi* | 0.5000 | NA | 1 |

**Supplementary Fig. 8. Meta-analysis of the prevalence of each species of pangolin-associated microbes.**

The heterogeneity of combined studies was quantified by $I^2$ statistic. The fixed effect model would be applied if $I^2 > 50\%$; Otherwise, the random effect model would be applied. Two-sided *P* values were provided in the plots.

***Manis javanica papillomavirus 1***

| Study | Events | Total | Proportion | 95%−CI | Weight (common) | Weight (random) |
|---|---|---|---|---|---|---|
| Barreat et al. 2022 *M. javanica* | 36 | 73 | 0.4932 | [0.3740; 0.6128] | 76.6% | 76.6% |
| Barreat et al. 2022 *M. pentadactyla* | 11 | 22 | 0.5000 | [0.2822; 0.7178] | 23.4% | 23.4% |
| **Common effect model** | | **95** | **0.4947** | **[0.3925; 0.5970]** | **100.0%** | **--** |
| **Random effects model** | | | **0.4947** | **[0.3925; 0.5970]** | **--** | **100.0%** |

Heterogeneity: $I^2 = 0\%$, $\tau^2 = 0$, $p = 0.96$

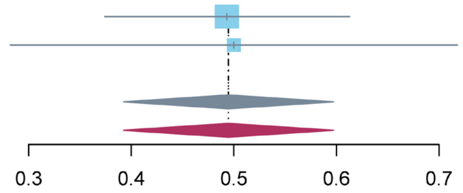


***Carnivore protoparvovirus 1***

| Study | Events | Total | Proportion | 95%−CI | Weight (common) | Weight (random) |
|---|---|---|---|---|---|---|
| Wang et al. 2020 *M. pentadactyla* | 1 | 1 | 1.0000 | [0.0250; 1.0000] | 0.9% | 8.9% |
| He et al. 2022 *M. pentadactyla* | 3 | 8 | 0.3750 | [0.0852; 0.7551] | 4.9% | 21.4% |
| Chang et al. 2021 *M. pentadactyla* | 4 | 32 | 0.1250 | [0.0351; 0.2899] | 18.6% | 27.5% |
| Shi et al. 2022 *M. javanica* | 29 | 129 | 0.2248 | [0.1560; 0.3066] | 74.2% | 29.8% |
| Zhang et al. 2022 *M. pentadactyla* | 2 | 2 | 1.0000 | [0.1581; 1.0000] | 1.4% | 12.4% |
| **Common effect model** | | **172** | **0.1560** | **[0.0857; 0.2370]** | **100.0%** | **--** |
| **Random effects model** | | | **0.3370** | **[0.0456; 0.6969]** | **--** | **100.0%** |

Heterogeneity: $I^2 = 65\%$, $\tau^2 = 0.0684$, $p = 0.02$

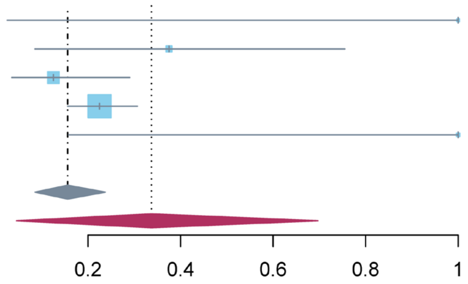


***Lishui pangolin virus***

| Study | Events | Total | Proportion | 95%−CI | Weight (common) | Weight (random) |
|---|---|---|---|---|---|---|
| Gao et al. 2020 *M. javanica* | 1 | 3 | 0.3333 | [0.0084; 0.9057] | 0.2% | 11.6% |
| Shi et al. 2022 *M. javanica* | 3 | 129 | 0.0233 | [0.0048; 0.0665] | 99.8% | 88.4% |
| **Common effect model** | | **132** | **0.0240** | **[0.0000; 0.0500]** | **100.0%** | **--** |
| **Random effects model** | | | **0.0591** | **[0.0000; 0.2535]** | **--** | **100.0%** |

Heterogeneity: $I^2 = 23\%$, $\tau^2 = 0.0109$, $p = 0.26$

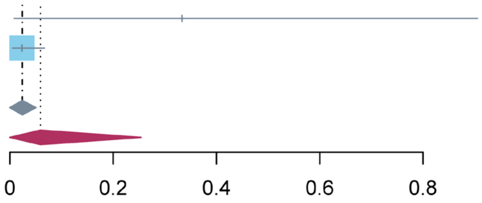


***Pangolin pestvirus***

| Study | Events | Total | Proportion | 95%−CI | Weight (common) | Weight (random) |
|---|---|---|---|---|---|---|
| Tian et al. 2022 *M. javanica* | 3 | 34 | 0.0882 | [0.0186; 0.2368] | 54.6% | 54.6% |
| Shi et al. 2022 *M. javanica* | 2 | 20 | 0.1000 | [0.0123; 0.3170] | 36.9% | 36.9% |
| Shi et al. 2022 *M. pentadactyla* | 0 | 16 | 0.0000 | [0.0000; 0.2059] | 8.6% | 8.6% |
| **Common effect model** | | **70** | **0.0843** | **[0.0380; 0.1874]** | **100.0%** | **--** |
| **Random effects model** | | | **0.0843** | **[0.0380; 0.1874]** | **--** | **100.0%** |

Heterogeneity: $I^2 = 0\%$, $\tau^2 = 0$, $p = 0.74$

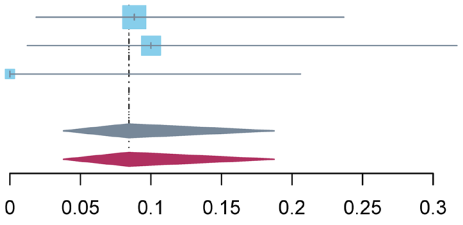

### *Dongyang pangolin virus*

| Study | Events | Total | Proportion | 95%−CI | Weight (common) | Weight (random) |
|---|---|---|---|---|---|---|
| Gao et al. 2020 *M. javanica* | 1 | 3 | 0.3333 | [0.0084; 0.9057] | 17.5% | 30.1% |
| Que et al. 2022 *M. javanica* | 12 | 16 | 0.7500 | [0.4762; 0.9273] | 82.5% | 69.9% |
| **Common effect model** | | **19** | **0.7073** | **[0.4535; 0.9165]** | **100.0%** | **--** |
| **Random effects model** | | | **0.6541** | **[0.2635; 0.9647]** | **--** | **100.0%** |

Heterogeneity: $I^2$ = 39%, $\tau^2$ = 0.0276, $p$ = 0.20

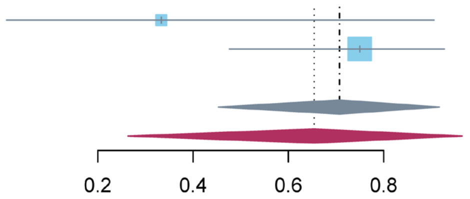


### *Pangolin coronavirus*

| Study | Events | Total | Proportion | 95%−CI | Weight (common) | Weight (random) |
|---|---|---|---|---|---|---|
| Liu et al. 2020 *M. javanica* | 3 | 11 | 0.2727 | [0.0602; 0.6097] | 0.4% | 7.0% |
| Lam et al. 2020 *M. javanica* | 10 | 67 | 0.1493 | [0.0740; 0.2574] | 3.3% | 10.4% |
| Xiao et al. 2020 *M. javanica* | 17 | 25 | 0.6800 | [0.4650; 0.8505] | 0.7% | 8.6% |
| Xiao et al. 2020 *M. pentadactyla* | 0 | 4 | 0.0000 | [0.0000; 0.6024] | 0.4% | 7.0% |
| Choo et al. 2020 *M. javanica* | 1 | 2 | 0.5000 | [0.0126; 0.9874] | 0.1% | 2.2% |
| Li et al. 2021 *M. javanica* | 5 | 46 | 0.1087 | [0.0362; 0.2357] | 3.0% | 10.4% |
| Li et al. 2021 *M. pentadactyla* | 0 | 4 | 0.0000 | [0.0000; 0.6024] | 0.4% | 7.0% |
| Peng et al. 2021 *M. javanica* | 4 | 106 | 0.0377 | [0.0104; 0.0938] | 18.4% | 11.0% |
| Peng et al. 2021 *M. pentadactyla* | 1 | 53 | 0.0189 | [0.0005; 0.1007] | 18.1% | 11.0% |
| Peng et al. 2021 *P. tetradactyla* | 0 | 1 | 0.0000 | [0.0000; 0.9750] | 0.1% | 2.7% |
| Peng et al. 2021 *P. tricuspis* | 0 | 3 | 0.0000 | [0.0000; 0.7076] | 0.2% | 5.9% |
| Nga et al. 2022 *M. pentadactyla* | 0 | 3 | 0.0000 | [0.0000; 0.7076] | 0.2% | 5.9% |
| Nga et al. 2022 *M. javanica* | 7 | 243 | 0.0288 | [0.0117; 0.0584] | 54.8% | 11.1% |
| **Common effect model** | | **568** | **0.0405** | **[0.0249; 0.0561]** | **100.0%** | **--** |
| **Random effects model** | | | **0.1249** | **[0.0108; 0.2390]** | **--** | **100.0%** |

Heterogeneity: $I^2$ = 81%, $\tau^2$ = 0.0305, $p$ < 0.01

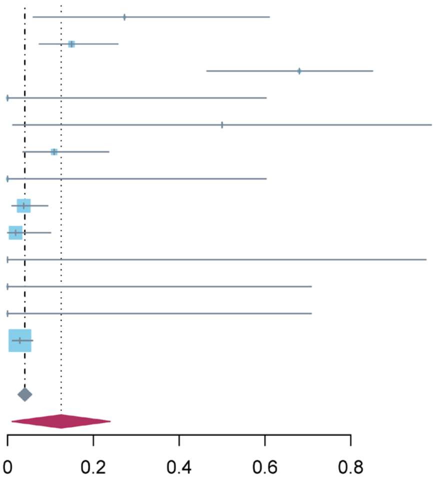


### SARS-CoV-2-related coronavirus

| Study | Events | Total | Proportion | 95%−CI | Weight (common) | Weight (random) |
|---|---|---|---|---|---|---|
| Liu et al. 2019 *M. javanica* | 2 | 11 | 0.1818 | [0.0228; 0.5178] | 60.5% | 60.5% |
| Wacharapluesadee et al. 2021 *M. javanica* | 1 | 7 | 0.1429 | [0.0036; 0.5787] | 39.5% | 39.5% |
| **Common effect model** | | **18** | **0.1650** | **[0.0158; 0.3895]** | **100.0%** | **--** |
| **Random effects model** | | | **0.1650** | **[0.0158; 0.3895]** | **--** | **100.0%** |

Heterogeneity: $I^2$ = 0%, $\tau^2$ = 0, $p$ = 0.90

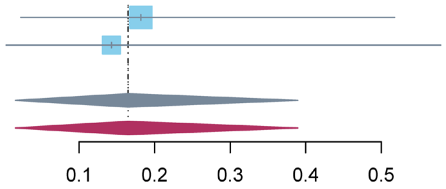


### *Human orthopneumovirus*

| Study | Events | Total | Proportion | 95%−CI | Weight (common) | Weight (random) |
|---|---|---|---|---|---|---|
| Ye et al. 2022 *M. javanica* | 12 | 30 | 0.4000 | [0.2266; 0.5940] | 17.3% | 33.3% |
| Shi et al. 2022 *M. javanica* | 7 | 129 | 0.0543 | [0.0221; 0.1086] | 73.4% | 33.9% |
| Que et al. 2022 *M. javanica* | 16 | 16 | 1.0000 | [0.7941; 1.0000] | 9.3% | 32.7% |
| **Common effect model** | | **175** | **0.1645** | **[0.1105; 0.2259]** | **100.0%** | **--** |
| **Random effects model** | | | **0.5003** | **[0.0001; 0.9999]** | **--** | **100.0%** |

Heterogeneity: $I^2$ = 98%, $\tau^2$ = 0.3610, $p$ < 0.01

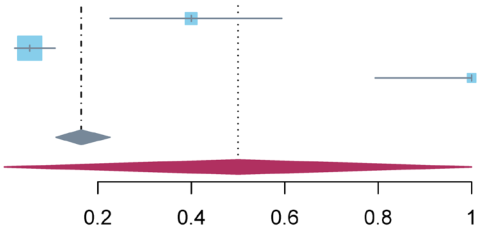

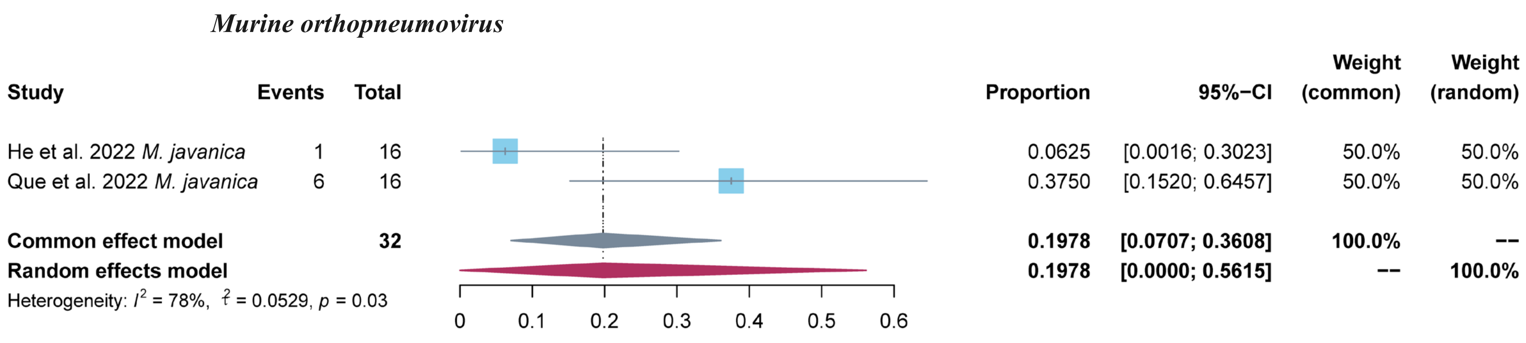
Murine orthopneumovirus
Study Events Total Proportion 95%−CI Weight (common) Weight (random)
He et al. 2022 M. javanica 1 16 0.0625 [0.0016; 0.3023] 50.0% 50.0%
Que et al. 2022 M. javanica 6 16 0.3750 [0.1520; 0.6457] 50.0% 50.0%
Common effect model 32 0.1978 [0.0707; 0.3608] 100.0% --
Random effects model 0.1978 [0.0000; 0.5615] -- 100.0%
Heterogeneity: $I^2$ = 78%, $\tau^2$ = 0.0529, p = 0.03
0 0.1 0.2 0.3 0.4 0.5 0.6

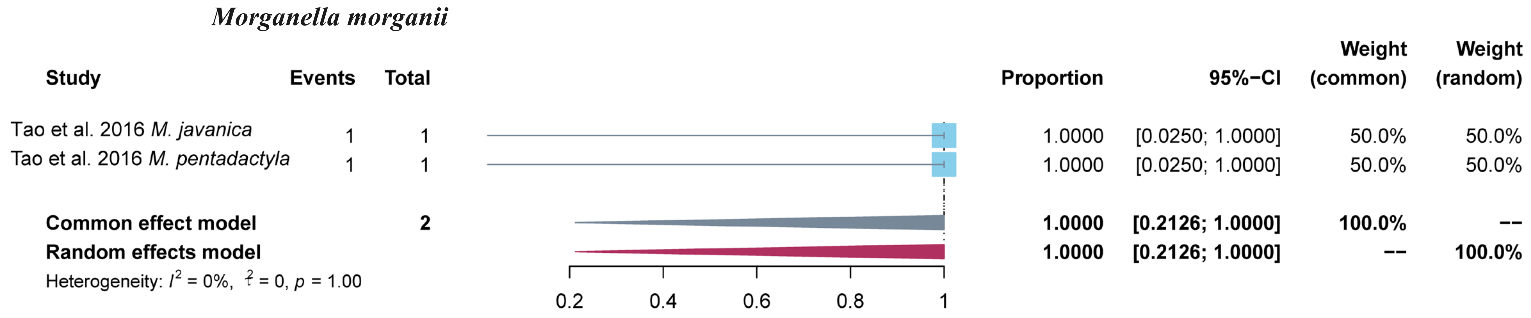
Morganella morganii
Study Events Total Proportion 95%−CI Weight (common) Weight (random)
Tao et al. 2016 M. javanica 1 1 1.0000 [0.0250; 1.0000] 50.0% 50.0%
Tao et al. 2016 M. pentadactyla 1 1 1.0000 [0.0250; 1.0000] 50.0% 50.0%
Common effect model 2 1.0000 [0.2126; 1.0000] 100.0% --
Random effects model 1.0000 [0.2126; 1.0000] -- 100.0%
Heterogeneity: $I^2$ = 0%, $\tau^2$ = 0, p = 1.00
0.2 0.4 0.6 0.8 1

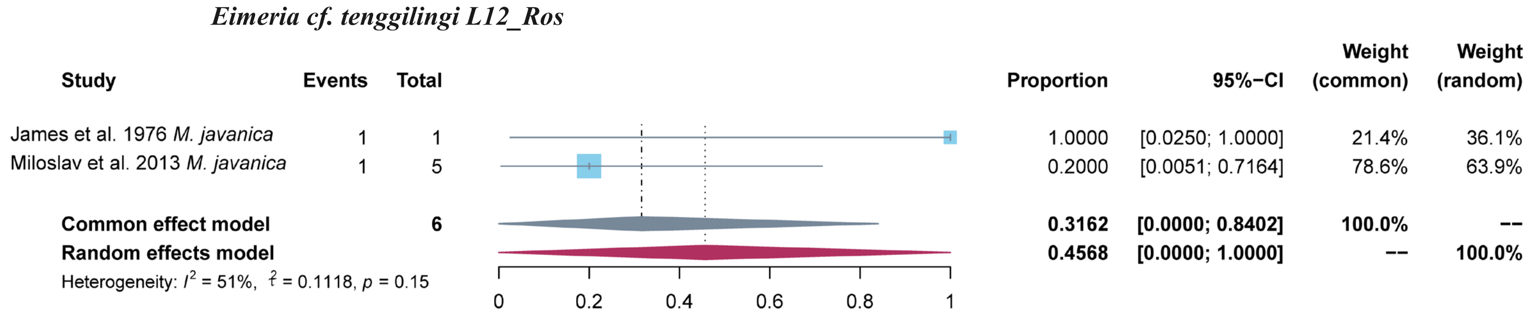
Eimeria cf. tenggilingi L12_Ros
Study Events Total Proportion 95%−CI Weight (common) Weight (random)
James et al. 1976 M. javanica 1 1 1.0000 [0.0250; 1.0000] 21.4% 36.1%
Miloslav et al. 2013 M. javanica 1 5 0.2000 [0.0051; 0.7164] 78.6% 63.9%
Common effect model 6 0.3162 [0.0000; 0.8402] 100.0% --
Random effects model 0.4568 [0.0000; 1.0000] -- 100.0%
Heterogeneity: $I^2$ = 51%, $\tau^2$ = 0.1118, p = 0.15
0 0.2 0.4 0.6 0.8 1

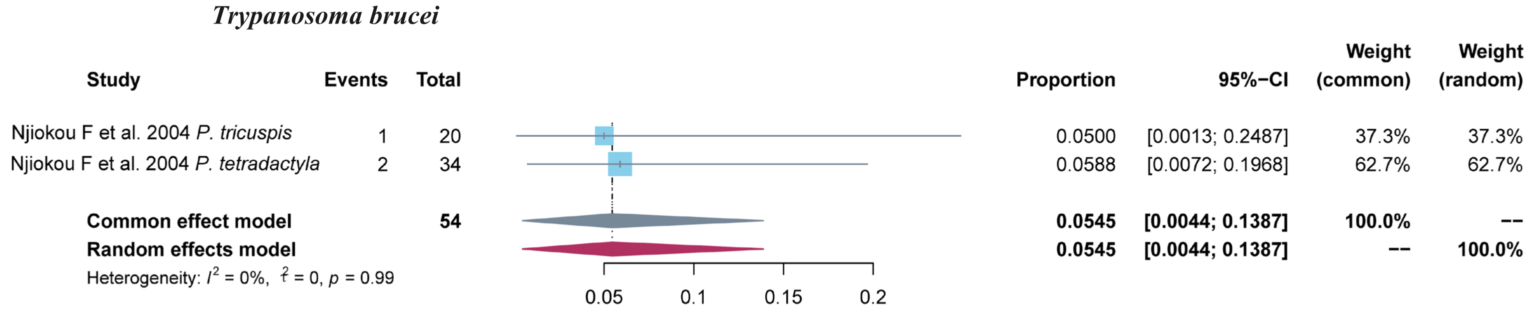
Trypanosoma brucei
Study Events Total Proportion 95%−CI Weight (common) Weight (random)
Njiokou F et al. 2004 P. tricuspis 1 20 0.0500 [0.0013; 0.2487] 37.3% 37.3%
Njiokou F et al. 2004 P. tetradactyla 2 34 0.0588 [0.0072; 0.1968] 62.7% 62.7%
Common effect model 54 0.0545 [0.0044; 0.1387] 100.0% --
Random effects model 0.0545 [0.0044; 0.1387] -- 100.0%
Heterogeneity: $I^2$ = 0%, $\tau^2$ = 0, p = 0.99
0.05 0.1 0.15 0.2

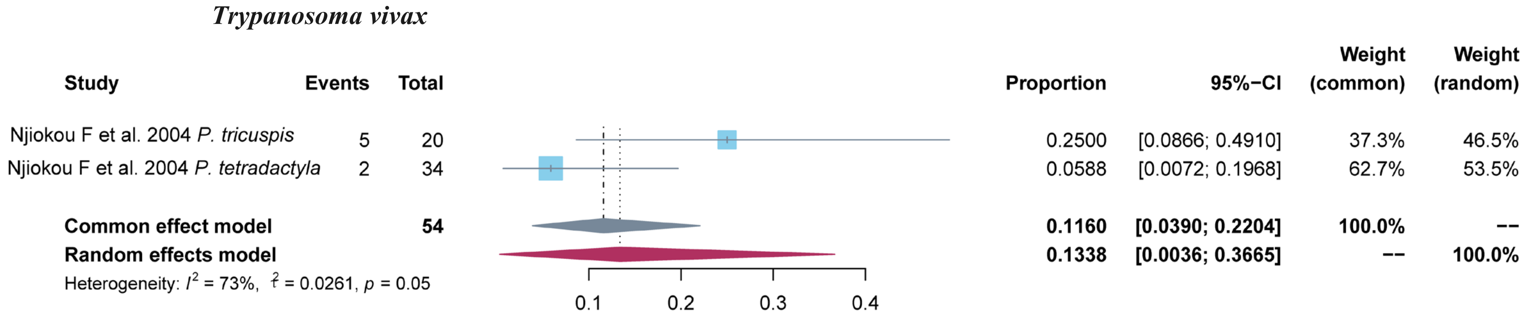
Trypanosoma vivax
Study Events Total Proportion 95%−CI Weight (common) Weight (random)
Njiokou F et al. 2004 P. tricuspis 5 20 0.2500 [0.0866; 0.4910] 37.3% 46.5%
Njiokou F et al. 2004 P. tetradactyla 2 34 0.0588 [0.0072; 0.1968] 62.7% 53.5%
Common effect model 54 0.1160 [0.0390; 0.2204] 100.0% --
Random effects model 0.1338 [0.0036; 0.3665] -- 100.0%
Heterogeneity: $I^2$ = 73%, $\tau^2$ = 0.0261, p = 0.05
0.1 0.2 0.3 0.4

**Supplementary Table 5. Univariable meta-regression of each family of pangolin-associated microbes***

| | **Coefficient** | ***P*-value**† |
|---|---|---|
| ***Papillomaviridae*** | | |
| Country | NA | NA |
| Species of pangolin | | |
| *Manis javanica* | Reference | |
| *Manis pentadactyla* | 0.8231 (-2.4093~4.0555) | 0.6177 |
| Sample category | NA | NA |
| Detection method | NA | NA |
| ***Parvoviridae*** | | |
| Country | NA | NA |
| Species of pangolin | | |
| *Manis javanica* | Reference | |
| *Manis pentadactyla* | 0.8649 (-0.5701~2.3000) | 0.2375 |
| Sample category | | |
| Trafficked | Reference | |
| Wild | 0.8649 (-0.5701~2.3000) | 0.2375 |
| Detection method | | |
| PCR | Reference | |
| Next generation sequencing | -0.7747 (-2.2108~0.6615) | 0.2904 |
| ***Flaviviridae*** | | |
| Country | NA | NA |
| Species of pangolin | | |
| *Manis javanica* | Reference | |
| *Manis pentadactyla* | -1.4529 (-3.4697~0.5639) | 0.1580 |
| Pangolin | -0.4341 (-2.2250~1.3568) | 0.6347 |
| Sample category | NA | NA |
| Captive | Reference | |
| Wild | 0.2858 (-0.2556~2.6272) | 0.8109 |
| Trafficked | 0.1377 (-2.0259~2.3014) | 0.7485 |
| Detection method | | |
| PCR | Reference | |
| Next generation sequencing | 1.4951 (-0.1938~3.1839) | 0.0827 |
| ***Coronaviridae*** | | |
| Country | | |
| China | Reference | |
| Vietnam | -0.0981 (-0.3534~0.1572) | 0.4514 |
| Thailand | 0.0248 (-0.3717~0.0412) | 0.9025 |
| Species of pangolin | | |
| *Manis javanica* | Reference | |
| *Manis pentadactyla* | -0.1338 (-0.3334~0.0659) | 0.1891 |
| *Phataginus tetradactyla* | -0.1413 (-0.8090~0.5264) | 0.6783 |
| *Phataginus tricuspis* | -0.1413 (-0.5781~0.2955) | 0.5261 |

| | | |
|---|---|---|
| Sample category | | |
| Captive | Reference | |
| Trafficked | 0.0443 (-0.2138~0.3023) | 0.7367 |
| Unknown catogary | 0.0752 (-0.3843~0.5346) | 0.7485 |
| Detection method | | |
| PCR | Reference | |
| Next generation sequencing | -0.0061 (-0.1706~0.1584) | 0.9423 |
| ***Paramyxoviridae*** | | |
| Country | NA | NA |
| Species of pangolin | NA | NA |
| Sample category | | |
| Trafficked | Reference | |
| Wild | -0.9539 (-5.2329~3.3250) | 0.6621 |
| Detection method | | |
| PCR | Reference | |
| Next generation sequencing | -1.6174 (-5.6583~2.4236) | 0.4328 |
| ***Pneumoviridae*** | | |
| Country | NA | NA |
| Species of pangolin | NA | NA |
| Sample category | | |
| Trafficked | Reference | |
| Wild | -0.4881 (-1.8730~0.8967) | 0.4896 |
| Detection method | NA | NA |

*At least two subjects were included in the meta-regression

†Two-sided *P*-values were reported

**Supplementary Table 6. Univariable meta-regression of each species of pangolin-associated microbes***

| | Coefficient | *P*-value† |
|---|---|---|
| ***Carnivore protoparvovirus 1*** | | |
| Country | NA | NA |
| Species of pangolin | | |
| *Manis javanica* | Reference | |
| *Manis pentadactyla* | 0.2735 (-0.5398~1.0867) | 0.5099 |
| Sample category | | |
| Trafficked | Reference | |
| Wild | 0.2735 (-0.5398~1.0867) | 0.5099 |
| Detection method | | |
| PCR | Reference | |
| Next generation sequencing | -0.2371 (-0.9627~0.4885) | 0.5219 |
| ***Pangolin coronavirus*** | | |
| Country | | |
| China | Reference | |
| Vietnam | -0.1281 (-0.4403~0.1840) | 0.4210 |
| Species of pangolin | | |
| *Manis javanica* | Reference | |
| *Manis pentadactyla* | -0.1970 (-0.4542~0.0601) | 0.1331 |
| *Phataginus tetradactyla* | -0.2037 (-0.9146~0.5072) | 0.5744 |
| *Phataginus tricuspis* | -0.2037 (-0.7040~0.2966) | 0.4249 |
| Sample category | | |
| Captive | Reference | |
| Trafficked | 0.0742 (-0.2419~0.3904) | 0.6554 |
| Detection method | | |
| PCR | Reference | |
| Next generation sequencing | 0.0898 (-0.2231~0.4028) | 0.5736 |

*At least two subjects were included in the meta-regression

†Two-sided *P*-values were reported

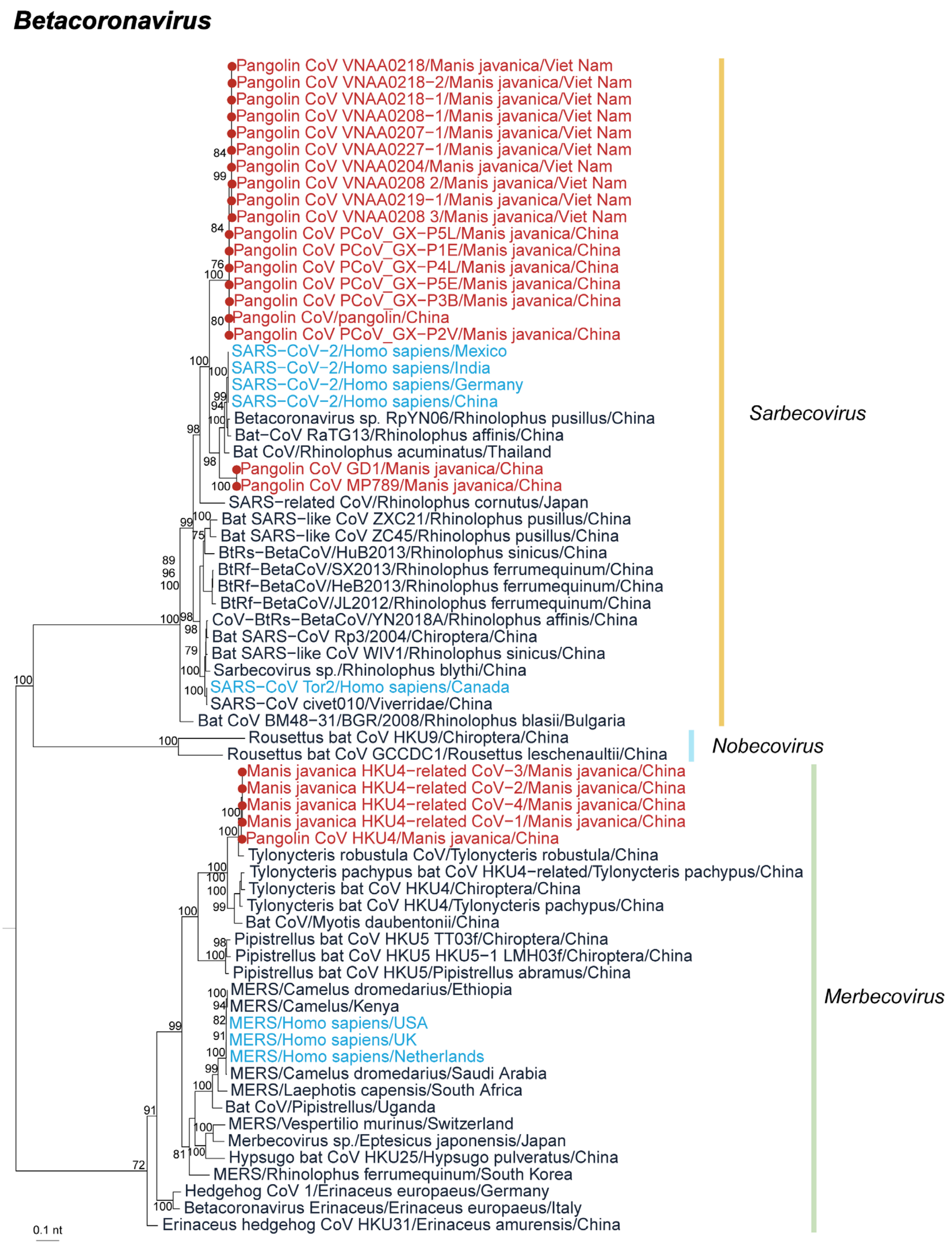


**Supplementary Fig. 9 Phylogenetic tree of *Betacoronavirus*.** Phylogeny of *Betacoronavirus* based on the RdRp domain (2790 nt).

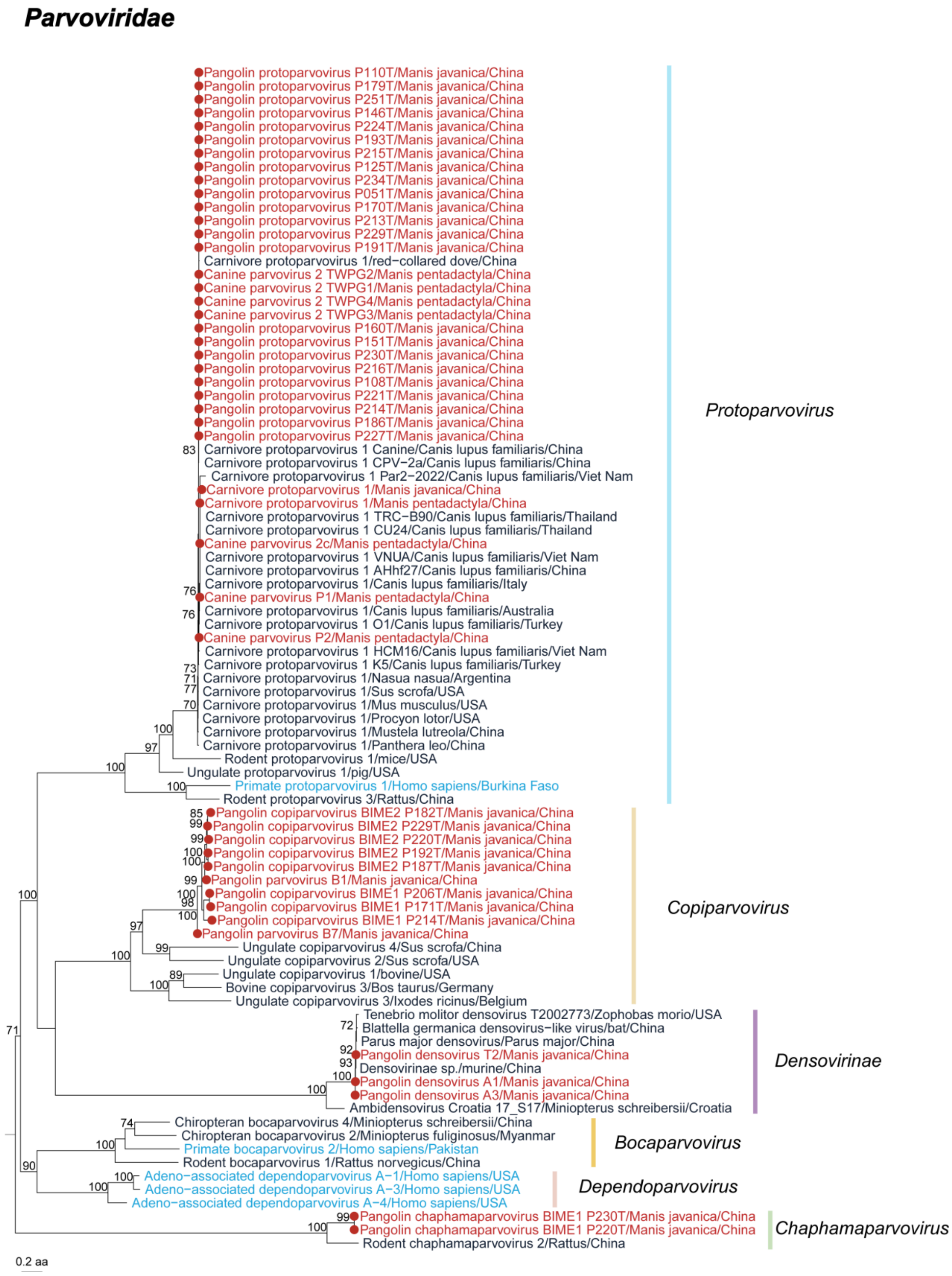


**Supplementary Fig. 10. Phylogenetic tree of *Parvoviridae***

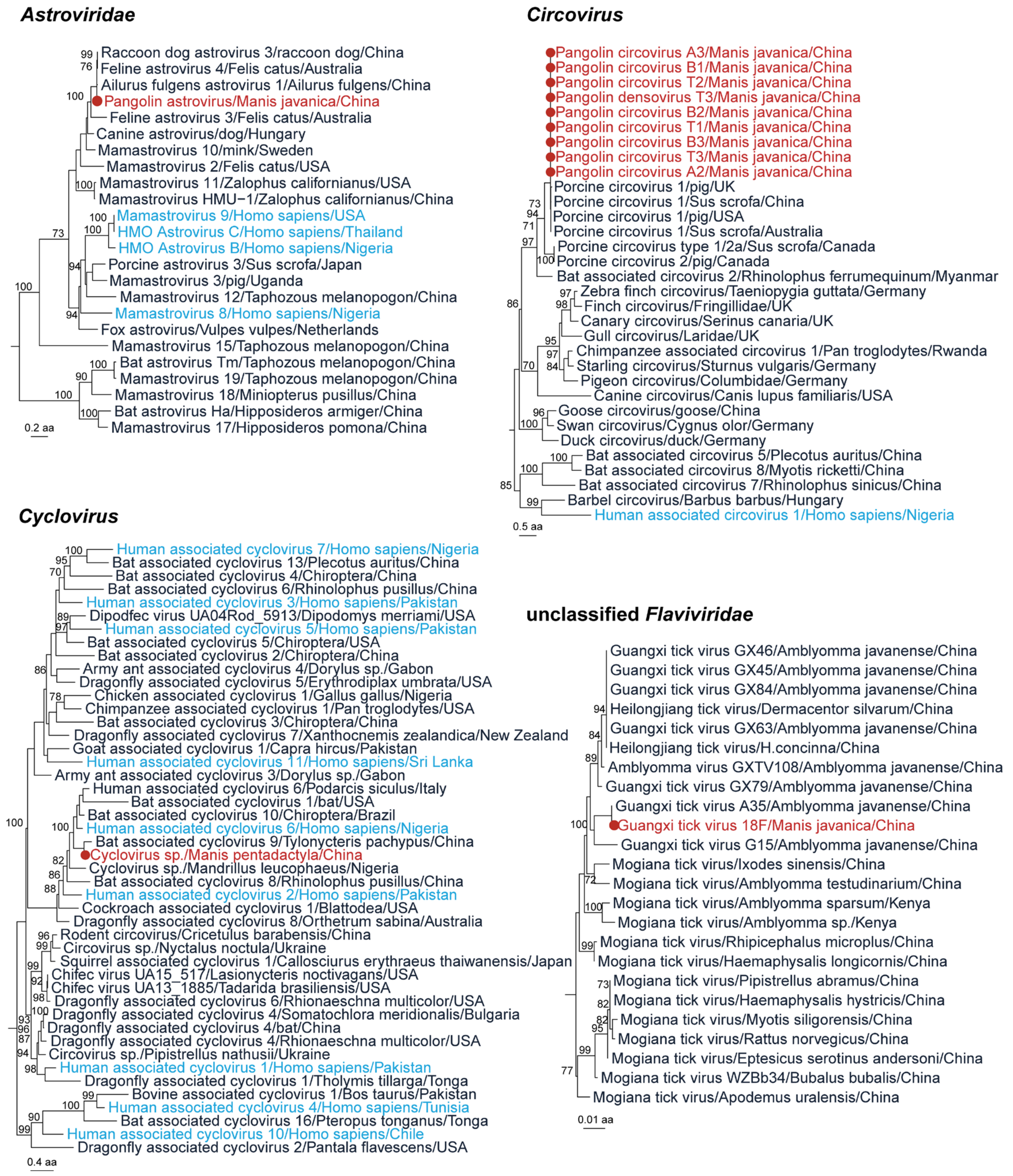


**Supplementary Fig. 11. Phylogenetic tree of animal-associated microbes**

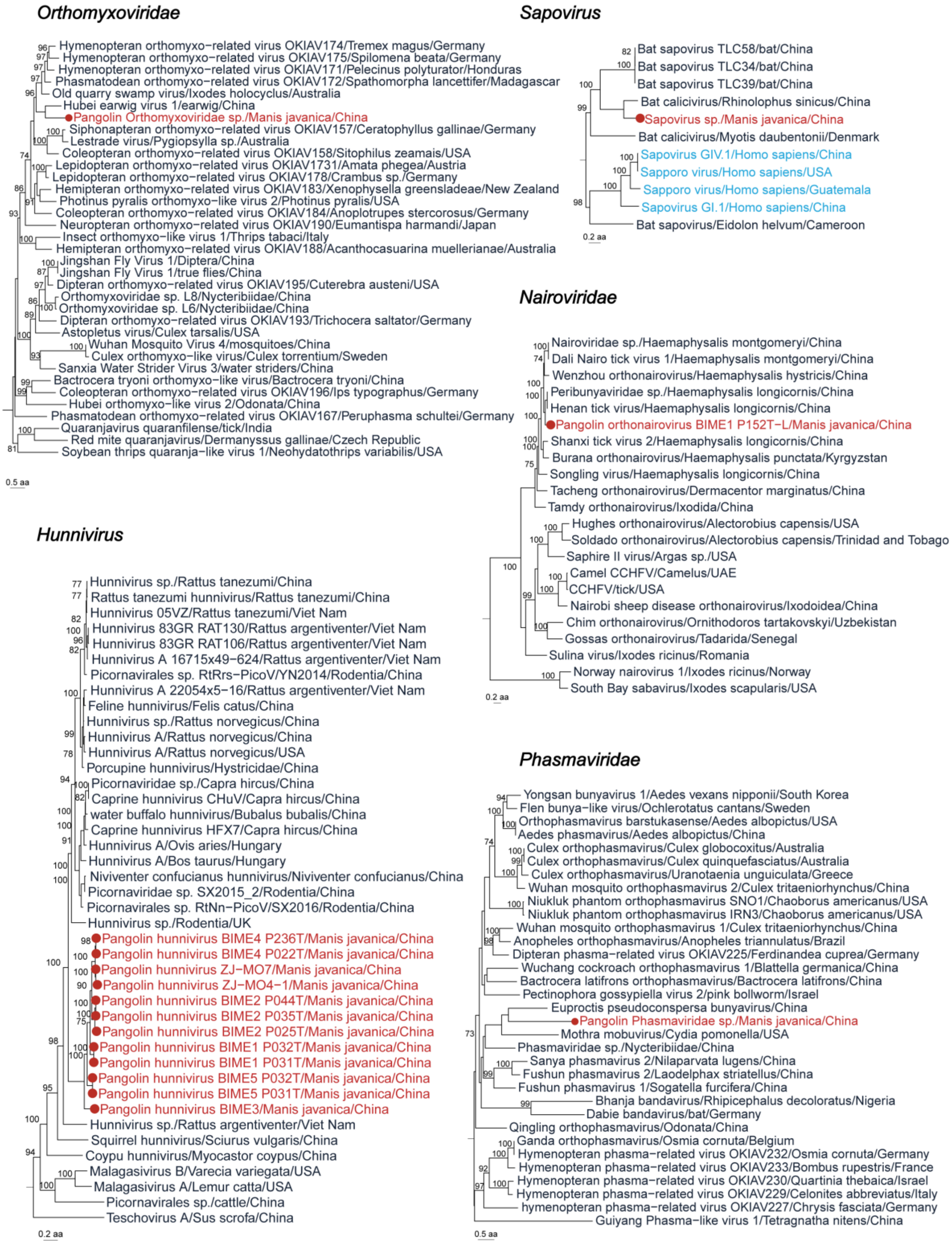


**Supplementary Fig. 12. Phylogenetic tree of pangolin-associated microbes**

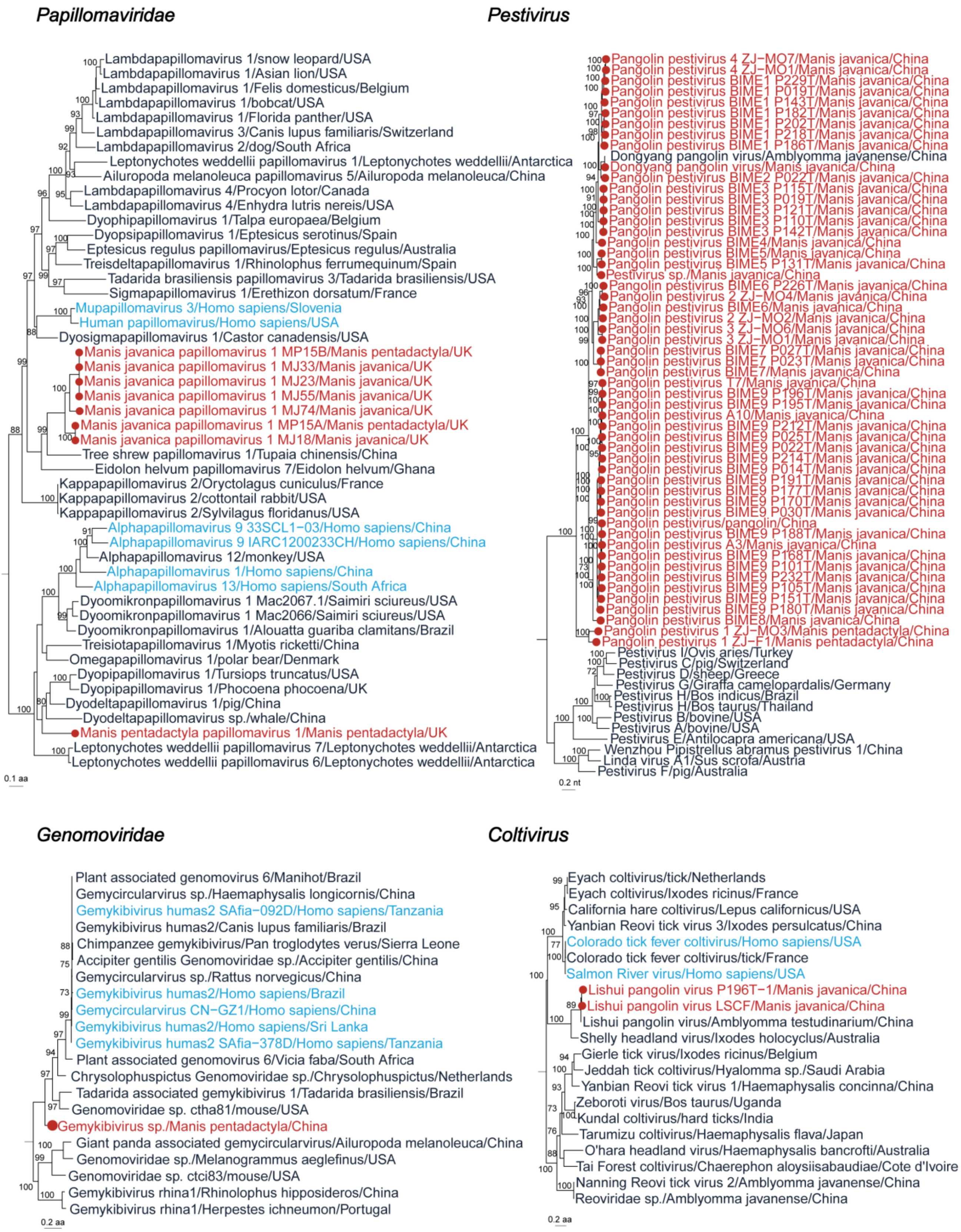


**Supplementary Fig. 13. Phylogenetic tree of pangolin-associated microbes**

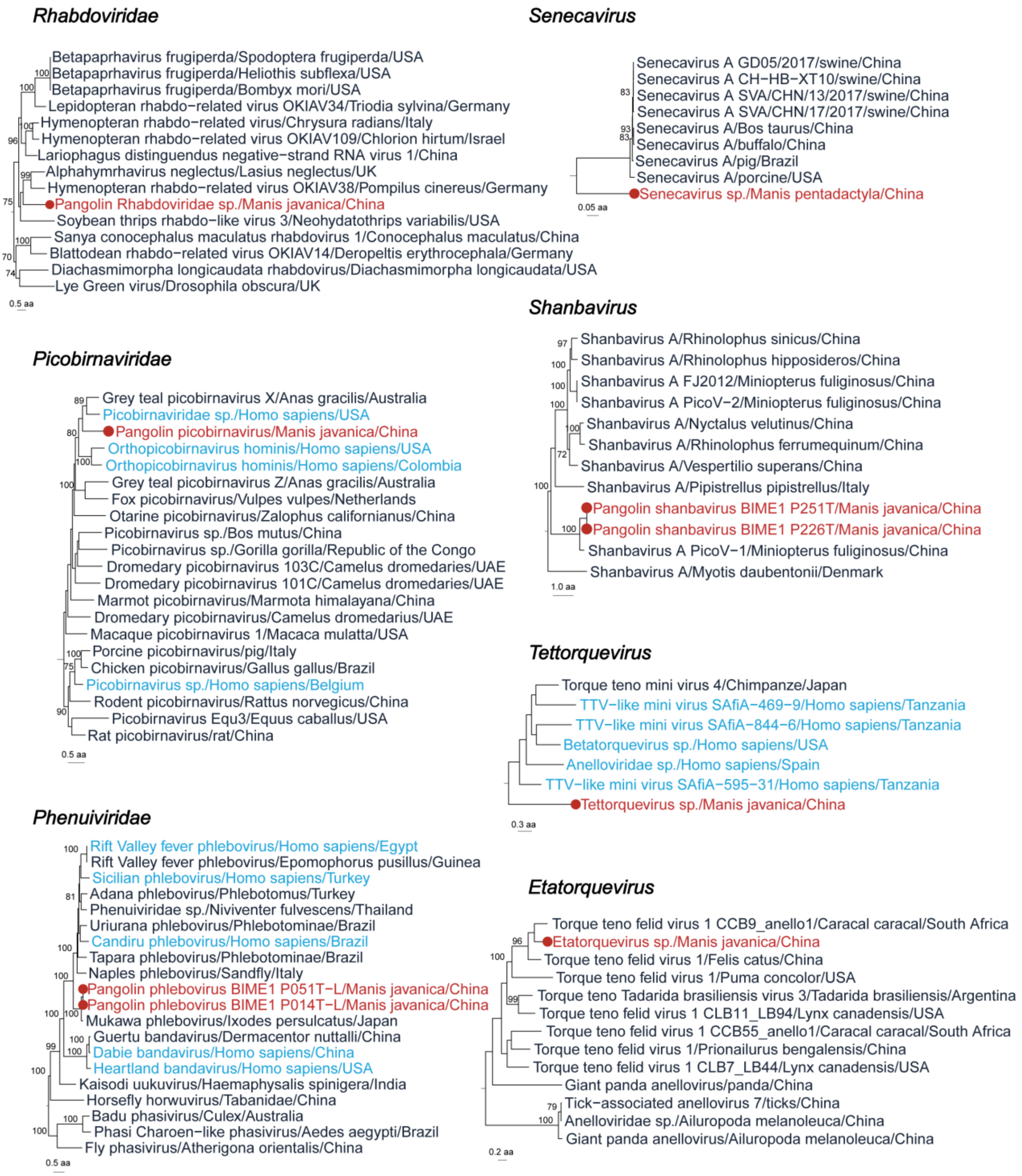


**Supplementary Fig. 14. Phylogenetic tree of pangolin-associated microbes**

**Supplementary Table 7. Check list of Latin and common names of pangolins**

| Latin name | Common name |
|---|---|
| *Manis crassicaudata* | Indian pangolin |
| *Manis culionensis* | Palawan pangolin |
| *Manis javanica* | Malayan pangolin |
| | Javan pangolin |
| | Sunda pangolin |
| *Manis pentadactyla* | Chinese pangolin |
| *Phataginus tetradactyla* | Long-tailed pangolin |
| *Phataginus tricuspis* | Tree pangolin |
| *Smutsia gigantea* | Giant pangolin |
| *Smutsia temminckii* | Ground pangolin |
| | Cape pangolin |
| | Temminck's pangolin |

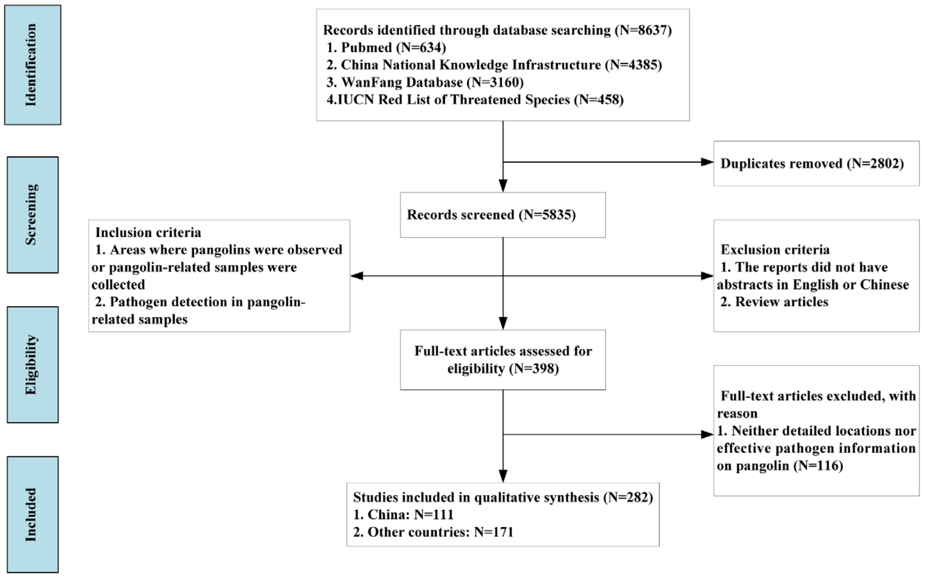


**Supplementary Fig. 15. PRISMA flow diagram of study selection process.** PRISMA flow diagram of study selection process.

## Supplementary Text 2. References for the literature review in this study

**In China (N=111):**


**1** Chen, J. et al. A bat MERS-like coronavirus circulates in pangolins and utilizes human DPP4 and host proteases for cell entry. *Cell* 186(4), 850–863 (2023).

2 Zhang, F. et al. A note on captive breeding and reproductive parameters of the Chinese pangolin, Manis pentadactyla Linnaeus, 1758. *Zookeys* (618), 129–144 (2016).

**3** Khatri-Chhetri, R. et al. A retrospective study of pathological findings in endangered formosan pangolins (*Manis pentadactyla pentadactyla*) from southeastern taiwan *Taiwan Veterinary Journal* **43**(1), 55–64 (2017).

**4** Bao, FY. et al. Air temperature changes in a burrow of Chinese pangolin, *Manis pentadactyla*, in winter. *Folia Zool.* **62**(1), 42–47 (2013).

**5** Choo, SW. et al. Are pangolins scapegoats of the COVID-19 outbreak-CoV transmission and pathology evidence? *Conservation Letters* **13**, e12754 (2020).

**6** Liu, P. et al. Are pangolins the intermediate host of the 2019 novel coronavirus (SARS-CoV-2)? *PLoS Pathog.* **17**(6), e1008421 (2020).

**7** Yu, Y., Wu, S., Wang, W., Mahmood, A., & Zhang, F. Body temperatures of *Manis pentadactyla* and *Manis javanica*. *Vet. M*ed. *Sci.* **7**(6), 2399–2403 (2021).

**8** Chang, YC. et al. Canine Parvovirus Infections in Taiwanese Pangolins (*Manis pentadactyla pentadactyla*). *Vet. Pathol.* **58**(4), 743–750 (2021).

**9** Ji, F. et al. Characteristics of the multiple replicon plasmid IncX1-X1 in multidrug-resistant Escherichia coli from Malayan pangolin (*Manis javanica*). *Integr. Zool.* **18**(2), 289–298, (2023).

**10** Li, HM. et al. Combined proteomics and transcriptomics reveal the genetic basis underlying the differentiation of skin appendages and immunity in pangolin. *Sci. Rep.* **10**(1), 14566, (2020).

**11** Liu, C. et al. Comparative study of gut microbiota from captive and confiscated-rescued wild pangolins. *J. Genet. Genomics* **48**(9), 825–835 (2021).

**12** Wang, X. et al. Complete Genome Sequence of Parainfluenza Virus 5 (PIV5) from a Sunda Pangolin (*Manis javanica*) in China. *J. Wildl. Dis.* **55**(4), 947–950 (2019).

**13** Sun, NC. et al. Complete mitochondrial genome of *Manis pentadactyla pentadactyla* (Mammalia: Pholidota), an endemic subspecies of Chinese pangolin: mitogenome characterisation and phylogenetic implications. *Biodivers Data J.* **9**, e77961 (2021).

**14** Wu, SH. et al. Cytogenetic analysis of the Formosan pangolin, *Manis pentadactyla pentadactyla* (Mammalia : Pholidota). *Zool. Stud.* **46**(4), 389–396 (2007).

**15** Shi, YN. et al. Detection of a novel Pestivirus strain in Java ticks (*Amblyomma javanense*) and the hosts Malayan pangolin (*Manis javanica*) and Chinese pangolin (*Manis pentadactyla*). *Front. Microbiol.* **13**, 988730 (2022).

**16** Sun, NC. et al. Digesta retention time and recovery rates of ants and termites in Chinese pangolins (*Manis pentadactyla*). *Zoo Biol.* **39**(3), 168–175 (2020).

**17** Nino Barreat, JG. et al. Discovery of novel papillomaviruses in the critically endangered Malayan and Chinese pangolins. *Biol. Letters* **19**(1), 20220464 (2023).

**18** Yan, D. et al. Effects of Chronic Stress on the Fecal Microbiome of Malayan Pangolins (*Manis javanica*) Rescued from the Illegal Wildlife Trade. *Curr. Microbiol.* **78**(3), 1017–1025 (2021).

**19** Li, L. et al. Epidemiological Study of Betacoronaviruses in Captive Malayan Pangolins. *Front. Microbiol.* **12**, 657439 (2021).

**20** Wang, SL. et al. Fatal canine parvovirus-2 (CPV-2) infection in a rescued free-ranging Taiwanese pangolin (*Manis pentadactyla pentadactyla*). *Transbound. Emerg. Dis.* **67**(3), 1074–1081 (2020).

**21** Lina, Z. et al. Fatal canine parvovirus type 2a and 2c infections in wild Chinese pangolins (*Manis pentadactyla*) in southern China. *Transbound. Emerg. Dis.* **69**(6), 4002–4008 (2022).

**22** Boundenga, L. et al. Haemosporidian Parasites of Antelopes and Other Vertebrates from Gabon, Central Africa. *PLoS One* **11**(2), e0148958 (2016).

**23** Chin, SC. et al. Hematologic and serum biochemical parameters of apparently healthy rescued formosan pangolins (*Manis pentadactyla pentadactyla*). *J. Zoo Wildl. Med.* **46**(1), 68–76 (2015).

**24** Han, Z. et al. Highly diverse ribonucleic acid viruses in the viromes of eukaryotic host species in Yunnan province, China. *Front. Microbiol.* **13**, 1019444 (2022).

**25** Yan, D. et al. High-quality genomes of pangolins: insights into the molecular basis of scale formation and adaption to myrmecophagous diet. *Mol. Biol. Evol.* **40**(1), msac262 (2023).

**26** Yang, L. et al. Historical data for conservation: reconstructing range changes of Chinese pangolin (Manis pentadactyla) in eastern China (1970-2016). *Proc. Biol. Sci.* **285**(1885), 20181084 (2018).

**27** Yang, CW. et al. History and dietary husbandry of pangolins in captivity. *Zoo Biol.* **26**(3), 223–230 (2007).

**28** Que, TC. et al. Human parainfluenza 3 and respiratory syncytial viruses detected in pangolins. *Emerg. Microbes Infect.***11**(1), 1657–1663 (2022).

**29** Zhai, J. et al. Identification of Amblyomma javanense and detection of tick-borne Ehrlichia spp. in confiscated Malayan Pangolins. *Int. J Parasitol-Par.***14**, 107–116 (2021).

**30** Lam, TT. et al. Identifying SARS-CoV-2-related coronaviruses in Malayan pangolins. *Nature* **583**(7815), 282–285 (2020).

**31** Lee, RH., Cheung, K., Fellowes, JR., & Guénard, B. Insights Into the Chinese Pangolin's (*Manis pentadactyla*) Diet in a Peri-Urban Habitat: A Case Study From Hong Kong. *Trop. Conserv. Sci.* **10**, (2017).

**32** Xiao, K. et al. Isolation of SARS-CoV-2-related coronavirus from Malayan pangolins. *Nature* **600**(7887), 286–289 (2020).

**33** Zhang, F. et al. Keeping and breeding the rescued Sunda pangolins (*Manis javanica*) in captivity. *Zoo Biol.* **36**(6), 387–396 (2017).

**34** Sun, NC., Pei, KJ., & Wu, LY. Long term monitoring of the reproductive behavior of wild Chinese pangolin (*Manis pentadactyla*). *Sci. Rep.* **11**(1), 18116 (2021).

**35** Arora, B., Jai-Chyi Pei, K., Feng Weng, C., & Ching-Min Sun, N. Measuring fecal metabolites of endogenous steroids using ESI-MS/MS spectra in Taiwanese pangolin, (order Pholidota, family Manidae, Genus: Manis): A non-invasive method for endangered species. *Gen. Comp. Endocr.* **299**, 113607 (2020).

**36** Duan, DY., Tang, JM., Chen, Z., Liu, GH., & Cheng, TY. Mitochondrial genome of *Amblyomma javanense*: a hard tick parasite of the endangered Malayan pangolin (*Manis javanica*). *Med. Vet. Entomol.* **34**(2), 229–235 (2020).

**37** Tuli, MD. et al. Molecular detection of a novel *Ancylostoma sp.* by whole mtDNA sequence from pangolin *Manis javanica*. *Parasite. Vector.* **15**(1), 70 (2022).

**38** Jiang, BG. et al. Molecular detection of novel borrelia species, *Candidatus* Borrelia Javanense, in *Amblyomma javanense Ticks* from pangolins. *Pathogens* **10**(6), 728 (2021).

**39** Zhang, HR. et al. Molecular tracing of confiscated pangolin scales for conservation and illegal trade monitoring in Southeast Asia. *Glob. Ecol. Conserv.* (4), 414–422 (2015).

**40** Chin, SC. et al. Monitoring the gestation period of rescued Formosan pangolin (*Manis pentadactyla pentadactyla*) with progesterone radioimmunoassay. *Zoo Biol*. **31**(4), 479–489 (2012).

**41** Sun, NC. et al. Mortality and morbidity in wild Taiwanese pangolin (*Manis pentadactyla pentadactyla*). *PLoS One* **14**(2), e0198230 (2019).

**42** Zhang, D. et al. Multiple novel mosquito-borne zoonotic viruses revealed in pangolin virome. *Front. Cell. Infect. Mi.* **12**, 874003 (2022).

**43** Ye, RZ. et al. Natural infection of pangolins with human respiratory syncytial viruses. *Curr. Biol.* **32**(7), R307–R308 (2022).

**44** Gao, WH. et al. Newly identified viral genomes in pangolins with fatal disease. *Virus Evol.* **6**(1), veaa020 (2020).

**45** Ning, S. et al. Novel putative pathogenic viruses identified in pangolins by mining metagenomic data. *J. Med. Virol.* **94**(6), 2500–2509 (2022).

**46** Sun, N.C.M., Sompud, J. & Pei, K.J.C. Nursing period, behavior development, and growth pattern of a newborn formosan pangolin (*Manis pentadactyla pentadactyla*) in the wild. *Trop. Conserv. Sci.* **11**, 1-6 (2018).

**47** Yang, R. et al. Pathogenicity and transmissibility of a novel respirovirus isolated from a Malayan pangolin. *J. Gen. Virol.* **102**(4), 001586 (2021).

**48** Khatri-Chhetri, R. et al. Reference intervals for hematology, serum biochemistry, and basic clinical findings in free-ranging Chinese Pangolin (*Manis pentadactyla*) from Taiwan. *Vet. Clin. Pathol.* **44**(3), 380–390 (2015).

**49** Zhang, F. et al. Reproductive behavior of the captive Sunda pangolin (*Manis javanica Desmarest*, 1822). *Zoo Biol*. **39**(2), 65–72 (2020).

**50** Chen, D. et al. Single cell atlas for 11 non-model mammals, reptiles and birds. *Nat. Commun.* **12**(1), 7083 (2021).

**51** Yan, D. et al. Successful captive breeding of a *Malayan pangolin* population to the third filial generation. *Commun. Biol.* **4**(1), 1212 (2021).

**52** Khatri-Chhetri, R. et al. Surveillance of ticks and associated pathogens in free-ranging Formosan pangolins (*Manis pentadactyla pentadactyla*). *Ticks Tick Borne Dis.* **7**(6), 1238–1244, (2016).

**53** Li, HF. et al. Survey of the termites (isoptera: kalotermitidae, rhinotermitidae, termitidae) in a formosan pangolin habitat. *Fla. Entomol.* **94**(3), 534–538 (2011).

**54** Sun, NCM. et al. The genetic structure and mating system of a recovered Chinese pangolin population (*Manis pentadactyla Linnaeus*, 1758) as inferred by microsatellite markers. *Glob. Ecol. Conserv.* **23**, e01195 (2020).

**55** Peng, MS. et al. The high diversity of SARS-CoV-2-related coronaviruses in pangolins alerts potential ecological risks. *Zool. Res.* **42**(6), 834–844 (2021).

**56** Wu, SB., Ma, GZ., Chen, H., & Liu NF. The status and conservation of pangolins in China. *Env. Sci.* (2011).

**57** Shi, W. et al. Trafficked Malayan pangolins contain viral pathogens of humans. *Nat. Microbiol.* **7**(8), 1259–1269 (2022).

**58** Chen, TH. et al. *Trichoskrjabinia Costata* from the pangolin, *Manis pentadactyla*. *CMJ* **60**, 81–83 (1941).

**59** Nash, HC. et al. Using local ecological knowledge to determine status and threats of the Critically Endangered Chinese pangolin (*Manis pentadactyla*) in Hainan, China. *Biol. Conserv.* **196**, 189–195 (2016).

**60** Liu, P., Chen, W., & Chen, J. P. Viral Metagenomics Revealed Sendai Virus and Coronavirus Infection of Malayan Pangolins (*Manis javanica*). *Viruses* **11**(11), 979 (2019).

**61** He, WT. et al. Virome characterization of game animals in China reveals a spectrum of emerging pathogens. *Cell* **185**(7), 1117–1129 (2022).

**62** Tian, FJ. et al. Virome in healthy pangolins reveals compatibility with multiple potentially zoonotic viruses. *Zool. Res.* **43**(6), 977–988 (2022).

**63** Yan, D. et al. Weaning period and growth patterns of captive Sunda pangolin (*Manis javanica*) cubs. *PLoS One* **17**(9), e0272020 (2022).

**64** Wang, Q. et al. Whole-genome resequencing of Chinese pangolins reveals a population structure and provides insights into their conservation. *Commun. Biol.* **5**(1), 821 (2022).

**65** Njiokou, F. et al. Wild fauna as a probable animal reservoir for *Trypanosoma brucei gambiense* in Cameroon. *Infect. Genet. Evol.* **6**(2), 147–153 (2006).

**66** 吴诗宝, 等. 穿山甲洞穴生态学初步研究. *应用生态学报* (3), 401–407 (2004).

**67** 钟艳丽, 等. 穿山甲人工饲料配方的分析对比. *广西畜牧兽医* **37**(5), 219–221 (2021).

**68** 李树荣, 等. 穿山甲体内寄生线虫的观察. *云南畜牧兽医* **2**, 21–22 (1998).

**69** 余经裕, 等. 重庆市中华穿山甲的生态地理分布及资源现状调查初报. *林业科技通讯* **06**, 41–43 (2016).

**70** 吴诗宝, 等. 大雾岭保护区穿山甲冬季生境选择. *生态学报* **6**, 1079–1086 (2003).

**71** 高陞, 等. 滇西边境地区涉案野生动物资源调查研究. *野生动物* **33**, 158–162(2012).

**72** 梁俊杰, 等. 广东恩平七星坑省级自然保护区野生鸟兽的红外相机调查. *野生动物学报* **43**(04), 1019–1026 (2022).

**73** 吴诗宝, 等. 广东省穿山甲种群数量调查与资源蕴藏量. *兽类学报* **4**, 270–276 (2002).

**74** 范宗骥, 等. 广东肇庆地区再现中华穿山甲. *野生动物学报* **40**(3), 811–813 (2019).

**75** 郭振泉. 广州哺乳类寄生线虫的研究——I.寄生在鲮鲤、果子狸和椰子猫的新种线虫. *动物学报* **1**, 60—112 (1958).

**76** 郭振泉. 广州哺乳类寄生线虫的研究——II.毛陈属(新)(*Trichochenia gen.nov.*)的三种线虫.*动物学报* **1**, 73—117 (1958).

**77** 莫锦华, 姬云瑞, 许涵, 李迪强 & 刘芳. 海南尖峰岭国家级自然保护区森林动态监测样地鸟类和兽类多样性. *生物多样性* **29**(6), 819–824 (2021)

**78** 李冬森. 海南尖峰岭林区哺乳动物空间分布格局和活动节律研究 [硕士]: 陕西理工大学; (2022).

**79** 颜文博, 等. 海南尖峰岭中华穿山甲的分布与保护现状. *生物多样性* **30**(6), 84–91 (2022).

**80** 范宗骥, 等. 基于红外相机技术对广东鼎湖山及其周边林地的鸟兽调查. *生物多样性* **28**(9), 1147–1153 (2020).

**81** 塔旗, 等. 基于最大熵生态位模型的中华穿山甲潜在适宜生境预测. *生态学报* **41**(24), 9941–9952 (2021).

**82** 冉重阳, 等. 救护前期穿山甲的日常行为表达. *林业与环境科学* **38**(5), 38–43 (2022).

**83** 郭瑞, 等. 利用红外相机技术调查浙江清凉峰国家级自然保护区的鸟兽多样性. *浙江林业科技* **42**(6), 63–72 (2022).

**84** 张玉林, 等. 利用红外相机监测中华穿山甲出入洞穴完整事件的影响因素探究. *野生动物学报* **44**(01), 31–37 (2023).

**85** 张富华, 等. 3 例人工圈养穿山甲死亡原因报告. *经济动物学报* **19**(3), 152–155 (2015).

**86** 伍智, 等. 马来穿山甲的脾脏组织结构研究. *经济动物学报* **26**(1), 29–31 (2022).

**87** 谭罗昊, 等. 马来穿山甲胃穿孔病因分析与预防性治疗. *湖北畜牧兽医* **42**(5), 19–21 (2021).
**88** 杜雪晴, 等. 马来穿山甲源嗜水气单胞菌亚种的分离鉴定及生物学特性分析. *中国动物检* **38**(4), 124–131 (2021).
**89** 傅道言 & 丁铁明. 鄱阳湖地区兽类资源调查. *动物学杂志* (02), 27–31 (1991).
**90** 徐爱春, 斯幸峰，王彦平 & 丁平. 千岛湖片段化栖息地地栖哺乳动物的红外相机监测及最小监测时长. *生物多样性* **22**(6), 764–772 (2014).
**91** 陈钧, 刘定震 & 韩品莲. 浅论台湾动物地理. *甘肃科学学报* **01**, 74–8 (1993).
**92** 王才益. 人工饲养穿山甲行为的观察. *经济动物学报* **03**, 41–44 (2000).
**93** 余经裕, 姜福林, 彭建军, 殷西林 & 马晓华. 人工饲养马来穿山甲(*Manis javanica*)全球首例繁殖报道（英文）. *Agricultural Science & Technology* **16**, 2322–2330 (2015).
**94** 庄馨, 曹世奎 & 胡观冠. 深圳大鹏半岛国家地质公园野生脊椎动物资源调查. *热带地理* **33**, 582–597 (2013).
**95** 何梅红, 等. 死亡马来穿山甲肠道中细菌的分离培养与鉴定. *广西畜牧兽医* 2021; **37:** 102–117.
**96** 李旭, 周杰珑, 郭子发, 郭爱伟 & 陈粉粉. 西双版纳地区穿山甲取食的几种蚂蚁营养成分分析. *四川动物* **29**, 620–621 (2010).
**97** 廖国宇, 彭建军, 黄飘逸, 余经裕 & 马晓华. 野生马来穿山甲被引入圈养后的适应行为分析. *重庆师范大学学报(自然科学版).* **35**, 42–47 (2018).
**98** 张富华, 邹翠云, 吴诗宝, 汪巧云 & 李韶山. 一例雌性马来穿山甲(*Manis javanica*)生殖系统大体解剖. *野生动物学报* **37**, 325–329 (2016).
**99** 张富华, 吴诗宝, 杨立, 李韶山 & 张莉. 一例圈养繁殖马来穿山甲畸形仔兽. *经济动物学报* **17**, 105–**108 (20**13).
**100** 燕洪美, 等. 一例中华穿山甲孕期激素监测和双胞胎卵型鉴定. *林业与环境科学* **38**, 18–24 (2022).
**101** 陶立, 等. 一起穿山甲疫情的病原分析. *动物医学进展* **37**, 133-136 (2016).
**102** 黄婧雪. 云南省西畴县兽类多样性的初步研究 [硕士]: 云南师范大学, (2018).
**103** 彭建军. 在人工饲养条件下同一年内连续繁殖成功马来穿山甲. *林业科技通讯* **11**, 81 (2016).
**104** 苏超. 中国穿山甲(*Manis pentadactyla*)洞穴生境选择及温度特征研究 [硕士]: 华南师范大学, (2011).
**105** 吴诗宝, 等. 中国穿山甲的食性与觅食行为初步观察. *应用与环境生物学报* **03**, 337–341 (2005).
**106** 吴诗宝. 中国穿山甲华南亚种(*Manis pentadactyla* aurita)仔兽出生记录. *青海师范大学学报 (自然科学版)* **01**, 41–43 (1998).
**107** 吴诗宝, 等. 中国兽类一新纪录——爪哇穿山甲.动物分类学报 **02**, 440–443 (2005).
**108** 李成. 中华穿山甲真实的种群状况. *森林与人类*, 124-128 (2022).
**109** 彭杰. 中华穿山甲（*Manis pentadactyla*）的生态地理分布、栖境选择及野生资源现状的研究 [硕士]: 重庆师范大学, (2020).
**110** 颉志刚，等. 中华穿山甲腹泻治疗前后粪便微生物群落组成的差异. *野生动物学报* **43**, 394–402 (2022).
**111** 陆珂静, 等. 中华穿山甲感染婿捲线虫(*Gendrespirura sp.*)的报告. *中国兽医杂志* **52**, 110–111+55 (2015)

**In other countries (N=171):**


**1** Jambari AE. et al. A camera trap assessment of terrestrial vertebrates in taman Negara Kelantan and Terengganu, Malaysia. *J Wildl. Parks* **30**, 45–57 (2015).

**2** Giman, B. et al. A camera trapping inventory for mammals in a mixed use planted forest in Sarawak. Raffles Bull. *Zool.* **55**, 209–215 (2007).

**3** Adeniyi, T. et al. A comparative study of the lateral geniculate body of rat (*Rattus norvegicus*), bat (*Eidolon helvum*) and pangolin (*Manis tricuspis*). *Glob. J. Health Sci.* **4**, 118–125 (2012).

**4** Steere, JB. A Month in Palawan. *The American Naturalist* **22**, 142–145 (1888).

**5** Mettrick DF. A New Tapeworm, *Inermicapsifer rhodesiensis sp. nov.* from a Scaly Ant-eater, *Manis temminckii*, in Southern Rhodesia. *J. Helminthol.* **33**, 273–276 (2009).

**6** Ee, CA. A note on breeding the cape pangolin *Manis temniincki* at bloemfontein zoo. *International Zoo Yearbook* **6** (2010).

**7** van Boom, KM. et al. A novel description of the Vastus lateralis morphology of the Temminck's ground pangolin (*Manis temminckii*). *Anat. Rec. (Hoboken)* **305**, 3463–3471 (2022).

**8** Pantel S. et al. A Preliminary Assessment of Sunda Pangolin Trade in Sabah. (Traffic Southeast Asia, Petaling Jaya, Malaysia Press, 2010).

**9** Timmins RJ. et al.An assessment of the conservation importance of the Huong Son (Annamite) Forest, Ha Tinh Province,Vietnam, based on the results of a field survey for large mammals and birds. *CMBC.* (1999).

**10** Shrestha S. et al. An Ecological Assessment of Critically Endangered Chinese Pangolin Manis pentadactyla (Mammalia: Pholidota: Manidae) in the Midhills Region of Nepal. *Open J. Obstet. Gynecol.* **11**, 344–356 (2021).

**11** Thapa, P. et al. An Overview of Chinese Pangolin (*Manis pentadactyla*): Its General Biology, Status, Distribution and Conservation Threats in Nepal. *Stud. Environ. Forum For. Res. Conserv.* **5** (2014).

**12** Perera P. et al. An update of distribution, habitats and conservation status of the Indian pangolin (*Manis crassicaudata*) in Sri Lanka. *Glob. Ecol. Conserv.* **21**, (2020).

**13** Difouo, GF. et al. Ant and termite prey of the giant pangolin *Smutsia gigantea* Illiger, 1815 in forest-savannah mosaics of Cameroon. *Afr. J. Ecol.* 59, 548–553 (2021).

**14** Katuwal, HB. et al. Anthropogenic impacts on the occurrence of the critically endangered Chinese pangolin (*Manis pentadactyla*) in Nepal. *J. Mammal.* **98**, 1667–1673 (2017).

**15** Acharya S. et al. Anthropogenic Threats to Survival of the Critically Endangered Chinese Pangolins (*Manis pentadactyla*) and their Habitat in Kavrepalanchowk, Nepal. *Hilaris SRL* (2018).

**16** Rayamajhi S. et al. Assessment of conservation threats and habitat management of Chinese pangolin (*Manis pentadactyla*) in Balthali VDC of Kavre Nepal. *Tribhuvan University Institute of Forestry, Pokhara* (2016).

**17** Mohapatra RK. et al. Behavioural Descriptions of Indian Pangolins (*Manis crassicaudata*) in Captivity. *Ital. J. Zool.* **2014**, 1–7 (2014).

**18** Pietersen DW, Behavioural ecology and conservation biology of ground pangolins *Smutsia temminckii* in the Kalahari Deser. University of Pretoria (2013).

**19** Mohapatra, RK. et al. Behavioural Sampling Techniques And Activity Pattern Of Indian Pangolin *Manis Crassicaudata* (Mammalia: Manidae) In Captivity. *J. Threatened Taxa* **5**, 5247–5255 (2013).

**20** Heath ME. et al. Biology, husbandry, and veterinary care of captive Chinese pangolins (*Manis pentadactyla*) *Zoo Biol*. **7**, 293–312 (1988).

**21** Masui, M. Birth of a Chinese pangolin *Manis pentadactyle* at Ueno Zoo, Tokyo. *International Zoo Yearbook* **7**, 114–116 (1967).

**22** Jones C. Body Temperatures of Manis-Gigantea and Manis-Tricuspis. *J. Mammal.* **54**, 263–266 (1973).

**23** Mahmood, T. et al. Breeding habits of the Indian pangolin (*Manis crassicaudata*) in Potohar Plateau, Pakistan. *Mammalia* **80**, 231–234 (2016).

**24** Mohd-Azlan, J. et al. Camera trapping and conservation in Lanjak Entimau Wildlife Sanctuary, Sarawak, Borneo. *Raffles B. Zool*. **61**, 397–405 (2013).

**25** Suzuki A. et al. Camera trapping of large mammals in Chhep Wildlife Sanctuary, northern Cambodia. *Cambodian Journal of Natural History* **1**, 63–75 (2017).

**26** Marler, PN. Camera Trapping The Palawan Pangolin *Manis Culionensis* (Mammalia: Pholidota: Manidae) In The Wild. *Journal of Threatened Taxa* **8**, 9443–9448 (2016).

**27** Kaicheen, SS. et al. Camera trapping wildlife on mount penrissen area in western sarawak. *Malaysian Applied Biology* **47**, 7–14 (2018).

**28** Jenks, KE., Songsasen, N. & Leimgruber, P. Camera trap records of dholes in Khao Ang Rue Nai Wildlife Sanctuary, Thailand. *Canid News*. (2012).

**29** Bruce, T. et al. Camera-Trap Survey for Larger Terrestrial Wildlife in the Dja Biosphere Reserve, Cameroon Diversity (2017).

**30** Treves, A. et al. Camera-trapping forest-woodland wildlife of western Uganda reveals how gregariousness biases estimates of relative abundance and distribution. *Biol. Conserv.* **143**, 521–528, (2010).

**31** Bernard, H. et al. Camera-trapping survey of mammals in and around imbak canyon conservation area in sabah, malaysian borneo. *Raffles B. Zool*. **61**(2), 861–870 (2013).

**32** Lahkar, D. et al. Camera-trapping survey to assess diversity, distribution and photographic capture rate of terrestrial mammals in the aftermath of the ethnopolitical conflict in Manas National Park, Assam, India. *J. Threatened Taxa* **10**(8), 12008–12017 (2018).

**33** Zanvo, S. et al. Can DNA help trace the local trade of pangolins? Conservation genetics of white-bellied pangolins from the Dahomey Gap (West Africa). *BMC Ecol. Evol.* **22**(1), 16 (2022).

**34** Sist, B. et al. Case report: Intestinal perforation and secondary peritonitis due to Acanthocephala infection in a black-bellied pangolin (*Phataginus tetradactyla*). *Parasitol. Int.* **80**, 102182 (2021).

**35** Dan, C. et al. Catalysing conservation action and raising the profile of pangolins- the IUCN-SSC Pangolin Specialist Group (PangolinSG). *AJCB.* **1**, 139–140 (2012).

**36** Aswathanarayana, NV. Chromosomes and karyotype of the Indian Pangolin, Manis crassicaudata Gray (Pholidota-Mammalia). *Cytologia (Tokyo)* **65**(4), 379–382 (2000).

**37** Monroe. et al. Collection and utilization of animal carcasses associated with zoonotic disease in Tshuapa District, the Democratic Republic of the Congo, 2012. *J. Wildlife Dis.* **51**(3), 734–738 (2015).

**38** Khatiwada, AP. et al. Community conservation in Nepal-opportunities and challenges for pangolin

conservation (Academic Press, 2020)

**39** Adeniyi PAO., Musa, AA., Bolaji, AO., &Ghazal, OK. Comparative histomorphometric and biochemical analysis of cerebral cortex in hedgehogs (Atelerix albiventris) and Pangolins (*Manis tricuspis*). *Asian Journal of Animal Sciences* **5**, 358–364 (2011).

**40** Tan, KY. et al. Comprehensive genome analysis of a pangolin-associated Paraburkholderia fungorum provides new insights into its secretion systems and virulence. *PeerJ.* **8**, e9733 (2020).

**41** Sparrow, S. Conservation veterinary nursing in Vietnam–Wound management in the Sunda pangolin, *Manis javanica*. *Veterinary Nursing Journal* **34**, 126–130 (2019).

**42** Harvey-Carroll, J. et al. Continued availability and sale of pangolins in a major urban bushmeat market in Cameroon despite national bans and the COVID-19 outbreak. *Afr. J. Ecol.* **60**(2), 146–152 (2022).

**43** Sulaiman, MH., Azmi, WA., Hassani, M & Chong, JL. Current updates on the morphological measurements of the Malayan pangolin (*Manis javanica*). *Folia Zool.* **66**(4), 260–266 (2017).

**44** Sweeney, RCH. CXV.—Some notes on the feeding habits of the Ground Pangolin, *Smutsia Temminckii* (Smuts). *Annals and Magazine of Nat. His.* **9**(108), 893–6 (2009).

**45** Karawita, H. & Perera, P. Dataset of distribution, habitats and conservation status of the Indian pangolin (*Manis crassicaudata*) in Sri Lanka. *Data brief.* **28**, 10499 (2020).

**46** Parola, P. et al. Detection of Ehrlichia spp., Anaplasma spp., *Rickettsia* spp., and other eubacteria in ticks from the Thai-Myanmar border and Vietnam. *JCM.* **41**, 1600–1608 (2003).

**47** Aguillon, S. et al. Development and characterization of 20 polymorphic microsatellite markers for the white-bellied pangolin *Phataginus tricuspis* (Mammalia, Pholidota). *Mol. Biol. Rep.* 47(6), 4827–4833 (2020).

**48** Karawita, H., Perera, P., Dayawansa, N. & Dias, S. Dietary composition and foraging habitats of the Indian Pangolin (*Manis crassicaudata*) in a tropical lowland forest-associated landscape in southwest Sri Lanka. *Glob. Ecol. Conserv.* **21**, 13 (2020).

**49** Thapa, P., Khatiwada, AP., Nepali, SC. & Paudel, S. Distribution and conservation status of Chinese pangolin (*Manis pentadactyla*) in Nangkholyang VDC, Taplejung, Eastern Nepal. *Am. J. Zool. Res.* **2**(1), 16–21 (2014).

**50** Mishra. S & Panda. S. Distribution of Indian Pangolin *Manis crassicaudata* Gray (Pholidota, Manidae) in Orissa: A rescue prospective. *Small Mammal Mail.* 4, 51–53 (2011)

**51** Irshad, N., Mahmood, T., Hussain, R. & Nadeem, MS distribution, abundance and diet of the Indian pangolin (*Manis crassicaudata*). *Anim. Biol.* **65**(1), 57–71 (2015).

**52** Mahmood, T. et al. Distribution, Abundance and vegetation analysis of the scaly ant-eater (*Manis crassicaudata*) in margalla hills National Park Islamabad, Pakistan. J. *Anim. Plant Sci.-JAPS.* **25**(5), 1311–1321 (2015).

**53** Dattagupta, S., Gupta, A. & Ghose, M. Diversity of non-timber forest products in Cachar District, Assam, India. *J. For. Res.* **25**(2), 463–470 (2014).

**54** Luiselli, L., Amori, G. & Akani, GC. Ecological diversity, community structure and conservation of Niger Delta mammals. *Biodivers. Conserv.* **24**(11), 2809–2830 (2015).

**55** Swart, JM., Richardson, PRK. & Ferguson, JWH. Ecological factors affecting the feeding behaviour of pangolins (*Manis temminckii*). *J. Zool.* **247**, 281–292 (1999).

**56** Arimoro FO., Kaine. EA., Krumale. BO. & Obiegba.S. Ecological observations, preliminary checklist and conservation of mammals occurring within the eastern boundaries of Ethiope River, Niger Delta Area of Nigeria. *J. Biodiversity, Bioprospect. Dev.* **01**(01) (2014).

**57** Akpona, HA., Djagoun, CAMS & Sinsin, B. Ecology and ethnozoology of the three-cusped pangolin *Manis tricuspis* (Mammalia, Pholidota) in the Lama forest reserve, Benin. *Mammalia* **72**(3), 198–202 (2008).

**58** Else, JG. & Colley FC. Eimeria-Tenggilingi Sp-N from scaly anteater *Manis-javanica* desmarest in Malaysia. *J. Protozool.* **23**(4), 487–488 (1976).

**59** Jamnah O. et al. Eperythrozoonosis (*Mycoplasma sp.*) in Malaysian Pangolin. *Malaysian J. Vet. Res.* **5**, 65–69 (2014).

**60** Sharma, S. et al. Estimating occupancy of Chinese pangolin (*Manis pentadactyla*) in a protected and non-protected area of Nepal. *Ecol. Evol.* **10**(10), 4303–4313 (2020).

**61** Mishra, N., Rout, SD. & Panda, T. Ethno-zoological studies and medicinal values of Similipal Biosphere Reserve, Orissa, India. *Afr. J. Pharm. Pharmacol.* **5**(1), 6–11 (2011).

**62** Wacharapluesadee, S. et al. Evidence for SARS-CoV-2 related coronaviruses circulating in bats and pangolins in Southeast Asia. *Nat. Commun.* **12**(1), 972(2021).

**63** Karim, MR. et al. Evidence for zoonotic potential of *Enterocytozoon Bieneusi* in its first molecular characterization in captive mammals at bangladesh national zoo. *J. Eukaryot. Microbiol.* **67**(4), 427–435 (2020).

**64** Nga, NTT. et al. Evidence of SARS-CoV-2 related Coronaviruses circulating in Sunda pangolins (*Manis javanica*) confiscated from the illegal wildlife trade in Viet Nam. *Front. Public Health* **10**, 826116 (2022).

**65** Jirku, M., Kvicerova, J., Modry, D. & Hypsa, V. Evolutionary Plasticity in Coccidia - Striking Morphological Similarity of Unrelated Coccidia (Apicomplexa) from Related Hosts: Eimeria spp. from African and Asian Pangolins (Mammalia: Pholidota). *Protist* **164**(4), 470–481 (2013).

**66** Shrestha, A., Bhattarai, S., Shrestha, B. & Koju, NP. Factors influencing the habitat choice of pangolins (*Manis* spp.) in low land of Nepal. *Ecol. Evol.* **11**(21)**,** 14689–14696 (2021).

**67** MacMillan, DC. & Nguyen, QA. Factors influencing the illegal harvest of wildlife by trapping and snaring among the Katu ethnic group in Vietnam. *Oryx* **48**(2), 304–312 (2014).

**68** Ingram, DJ., Awol, PP., Ding, KA. & Schen, A. First records of pangolin trafficking in South Sudan. *Afr. J. Ecol.* **58**(1), 133–137, (2020).

**69** Yodsheewan, R. et al. First report on detection of Babesia spp. in confiscated Sunda pangolins (*Manis javanica*) in Thailand. *Vet. World* **14**(4), 2380–2385 (2021).

**70** Tamang, S. et al. Foraging Burrow Site Selection and Diet of Chinese Pangolins, Chandragiri Municipality, Nepal. *Animals* **12**(19), 12 (2022).

**71** Fa, JE. et al. Getting to grips with the magnitude of exploitation: Bushmeat in the cross-Sanaga Rivers region, Nigeria and Cameroon. *Biol. Conserv.* **129**(4), 497–510 (2006).

**72** Bhandari, N. & Chalise, MK. Habitat and distribution of Chinese Pangolin (*Manis Pentadactyla* Linnaeus, 1758) in Nagarjun Forest of Shivapuri Nagarjun National Park, Nepal. *Nepalese Journal of Zoology* **2**, 18 (2014).

**73** Sapkota R. & Dhamala, MK. Habitat preference and burrowing habits of Chinese Pangolin: A

case from Shivapuri and Nagarjun National of Nepal. *Central department of environmental science* **2**(1) (2016).

**74** Karawita, H., Perera, P., Gunawardane, P. & Dayawansa, N. Habitat preference and den characterization of Indian Pangolin (*Manis crassicaudata*) in a tropical lowland forested landscape of southwest Sri Lanka. *PloS one* **13**(11), e0206082 (2018).

**75** Mahmood, T., Irshad, N. & Hussain, R. Habitat preference and population estimates of Indian pangolin (*Manis crassicaudata*) in district Chakwal of Potohar Plateau, Pakistan. *Russ. J. Ecol.* **45**(1), 70–75, (2014).

**76** Connelly, E., Hywood, L., Donaldson, M. & Pietersen, DW._Haematology and biochemistry values for Temminck's pangolins (*Smutsia temminckii*) from Zimbabwe. *Afr. Zool.* **55**(2), 161–165 (2020).

**77** Ahmad, AA.,Sekar, S., Oh, PY & Samsuddin, S. Hematology and serum biochemistry of captive Sunda pangolin (*Manis javanica*) in Wildlife Reserves Singapore. *J. Vet. Med. Sci.* **83**(2), 309–314 (2021).

**78** Ziegler, S. High mammalian diversity in the newly established National Park of Upper Niger, Republic of Guinea. *Oryx* **36**(2), 73–80 (2002).

**79** Gary, TNE. et al. Holistic managment of live animals confiscated from illegal wildlife trade. *Journal of Applied Ecology* **54**, 726–730 (2017).

**80** Heath, ME. et al. Home range size and distribution in a wild population of Cape pangolins, *Manis temminckii*, in north-west Zimbabwe. *Afr. J. Ecol.* 35, 94–109 (1997).

**81** Lim, NTL. et al. Home range, activity cycle and natal den usage of a female Sunda pangolin Manis javanica (Mammalia : Pholidota) in Singapore. *Endangered Species Res.* **4**, 233–240 (2008).

**82** Pietersen, DW. et al. Home range, habitat selection and activity patterns of an arid-zone population of Temminck's ground pangolins, Smutsia temminckii. *Afr. Zool.* **49**(2), 265–276 (2014).

**83** Kvicerova, J. & Hypsa, V. Host-parasite incongruences in rodent eimeria suggest significant role of adaptation rather than cophylogeny in maintenance of host specificity. *PloS one* **8**(7), e63601 (2013).

**84** Ofori. BY. & Attuquayefio, DK. Hunting intensity in the suhuma forest reserve in the sefwi wiawso district of the western region of Ghana: a threat to biodiversity conservation. *West African Journal of Applied Ecology* **17**, 135–142 (2010).

**85** Wilson AE. Husbandry of pangolins *Manis* spp. *New Developments In The Zoo World* **33**, 248–251 (1994).

**86** Mohapatra, RK. et al. Husbandry, behaviour and conservation breeding of Indian pangolin. *Folia Zool.* **63**(2), 73–80 (2014).

**87** Sai, B., Nasution, Z. & Wahyuningsih, H. Identification of terrestrial fauna biodiversity as an effort to reduce the wildlife conflict in Batang Angkola protected forest. *IOP Conf. Ser., Earth Environ. Sci. (UK)* 782 (2021).

**88** Mahmood, T., Hussain, R., Irshad, N., Akrim, F. & Nadeem, MSN. llegal Mass Killing of Indian Pangolin (*Manis crassicaudata*) in Potohar Region, Pakistan. *Pak. J. Zool.* **44**(5), 1457–1461 (2012).

**89** Zhang, MX. et al. Illegal pangolin trade in northernmost Myanmar and its links to India and China. *Glob. Ecol. Conserv.* **10**, 23–31 (2017).

**90** Kumar VP., Ankita, R., Thakur, M. & Shukla, M. et al. Illegal trade of Indian Pangolin (*Manis crassicaudata*): Genetic study from scales based on mitochondrial genes. *Egypt. J. Forensic Sci.* **6**, 524–533 (2016).

**91** Fa, JE., Juste, J.,Val, JPD. & Castroviejo, J. Impact of Market Hunting on Mammal Species in Equatorial Guinea. *Conserv. Bio.* **9**, 1107–1115 (1995).

**92** Kumpel NF. Incentives for sustainable hunting of bushmeat in Rio Muni, Equatorial Guinea. . Imperial College London (2006).

**93** Shrestha B. et al. Indirect methods of identifying mammals: a case study from Shivapuri National Park, Nepal. *Ecoprint An Inter. J. Ecology* **12** (2010).

**94** Njiokou, F. et al. Infection rate of Trypanosoma brucei s.l., T-vivax, T-congolense "forest type", and T-simiae in small wild vertebrates in south Cameroon. *Acta Trop*. **92**, 139–146 (2004).

**95** Segniagbeto, GH. et al. Insights into the status and distribution of pangolins in Togo (West Africa). *Afr. J. Ecol.* **59**, 342–349 (2021).

**96** Matthews A. et al. Inventory of large and medium-sized mammals in south-western Cameroon / Inventaire des mammifères de moyenne et de grande taille dans le sud-ouest Cameroun. *Mammalia* **70** (2006).

**97** DiPaola, JD. et al. Investigating the use of sensory information to detect and track prey by the Sunda pangolin (*Manis javanica*) with conservation in mind. *Sci. Rep.* **10**, 10 (2020).

**98** Sharma, S. et al. Knowledge of the Critically Endangered Chinese pangolin (Manis pentadactyla) by local people in Sindhupalchok, Nepal. *Glob. Ecol. Conserv.* **23**, 9 (2020).

**99** Malimbo, DK. et al. Local Perception on the Exploitation, the Current State and Taboos Related to Pangolins (Pholidota, Mammalia) by the Communities Living in the Tayna Nature Reserve and Its Surroundings (RNT) North Kivu DRC. *J. Geosci. Env. Prot.* **08**, 18-35 (2020).

**100** Bruce, T. et al. Locating Giant Ground Pangolins (*Smutsia gigantea*) Using Camera Traps on Burrows in the Dja Biosphere Reserve, Cameroon. *Trop. Conserv. Sci.* **11**, 1–5 (2018).

**101** Ojuolape S. et al. Lungs of Bat (*Eidolon helvum*), RAT (*Rattus norvegicus*) and Pangolin (*Manis tricuspis*): A Comparative Histology. Journal of Applied Life *Sciences International* **6**: 1–4 (2016).

**102** Kitamura, S. et al. Mammal diversity and conservation in a small isolated forest of southern thailand. *Raffles Bull. Zool.* **58**, 145–156 (2010).

**103** Phan, T. Mammal Observations in Cat Tien National Park. (2002).

**104** Dorji, S., Rajaratnam, R. & Vernes, K. Mammal richness and diversity in a Himalayan hotspot: the role of protected areas in conserving Bhutan's mammals. *Biodivers. Conserv.* 28, 3277–3297, doi:10.1007/s10531-019-01821-9 (2019).

**105** Shrestha, S. et al. Mitochondrial DNA analysis of critically endangered Chinese Pangolins (Manis pentadactyla) from Nepal. *Mitochondrial DNA Part B-Resour.* **5**, 3275–3279 (2020).

**106** Koh, FX. et al. Molecular detection of Anaplasma spp. in pangolins (*Manis javanica*) and wild boars (*Sus scrofa*) in Peninsular Malaysia. *Vet. Parasitol.* 227, 73–76 (2016).

**107** Irshad, N. et al. Morpho-anatomical characteristics of Indian pangolin (*Manis crassicaudata*) from Potohar Plateau, Pakistan. *Mammalia* **80**, 103–110 (2016).

**108** Behangana, M. et al. Nationally Threatened Species for Uganda. (NGO Press. 2016).

**109** Kollars, TMJ., Sithiprasasna, R. New host and distribution record of Amblyomma javanense

(Acari : Ixodidae) in Thailand. *J. Med. Entomol.* **37**(4), 640 (2000).

**110** Richard, P. Niger's Threatened Park W. *Oryx* **12**(2), 216–222 (1973).

**111** Lee, J. et al. No Evidence of Coronaviruses or Other Potentially Zoonotic Viruses in Sunda pangolins (*Manis javanica*) Entering the Wildlife Trade via Malaysia. *EcoHealth* **17**(3), 406–418 (2020).

**112** Thapa, A. et al. Non-protected areas demanding equitable conservation strategies as of protected areas in the Central Himalayan region. *PloS one* **16**(8), 14 (2021).

**113** Ogilvie PW., Bridgwater, DD. Notes on the breeding of an Indian pangolin *Manis Crassicaudats* at Oklahoma Zoo. *International Zoo Yearbook* **7**(1), 116–118 (1967).

**114** Gomez, L. et al. Observations of the illegal pangolin trade in Lao PDR. *Traffic Report* (2016).

**115** Waseem, M. et al. Occupancy, habitat suitability and habitat preference of endangered indian pangolin (*Manis crassicaudata*) in Potohar Plateau and Azad Jammu and Kashmir, Pakistan. *Glob. Ecol. Conserv.* **23**, 10 (2020).

**116** Singh, SN. On a new nematode Leipernema leiperi n.g., n.sp. (Strongyloididae), parasitic in the pangolin *Manis pentadactyla* from Hyderabad, India. *J. of Helminthol.* **50**(4), 267–274 (1976).

**117** Bhattarai GP. et al. Pangolin Conservation Action Plan for Nepal (2018-2022). (*Department of National Parks and Wildlife Conservation and Department of Forests.* Press, 2018).

**118** Trageser, SJ. et al. Pangolin distribution and conservation status in Bangladesh. *PloS one* **12**(4) (2017).

**119** Sodeinde, OA. et al. Pangolins in south-west Nigeria–current status and prognosis. *Oryx* **28**(1), 43–50 (1994).

**120** Mohapatra, RK., Banik, A., Sahu, SK., Panda, S., Danger, TK. Parasites and bacteria associated with Indian pangolins *Manis crassicaudata* (Mammalia: Manidae). *Glob. Ecol. Conserv*. **23**, 9 (2020).

**121** Gaubert, P. et al. Phylogeography of the heavily poached African common pangolin (Pholidota, Manis tricuspis) reveals six cryptic lineages as traceable signatures of Pleistocene diversification. *Mol. Ecol.* **25**(23), 5975–5993 (2016).

**122** Prter N. Potential applications of hunters' knowledge for the conservation of pangolins in Vietnam. *University of East Anglia, Norwich* (2007).

**123** Sharma, HP. et al. Potential Distribution of the Critically Endangered Chinese Pangolin (Manis pentadactyla) in Different Land Covers of Nepal: Implications for Conservation. *Sustainability* **12**(3), 13 (2020).

**124** Heath, ME., Coulson, LM. et al. Preliminary studies on relocation of Cape pangolins Manis temminckii. *South Afr. J. Wildl. Res.* **27**(2), 51–56 (1997).

**125** Jambari, A. et al. Quantifying species richness and composition of elusive rainforest mammals in Taman Negara National Park, Peninsular Malaysia. *Glob. Ecol. Conserv.* **18**, 10 (2019).

**126** Mahmood, T., Kanwal, K., Iftikhar-Uz-Zaman. Records of the indian pangolin (mammalia: Pholidota: Manidae: Manis crassicaudata) from mansehra district, pakistan. *Journal of Threatened Taxa* **10(2)**, 11254–11261 (2018).

**127** Hooijberg, EH., Lourens, K., Meyer, LCR. Reference Intervals for Selected Hematology and Clinical Chemistry Measurands in Temminck's Pangolin (*Smutsia temminckii*). *Front. Vet. Sci.* **8**, 16 (2021).

**128** Yu, J. et al. Serum Biochemistry And Select Mineral Parameters Of Pre-Release Sunda Pangolins (Manis Javanica) Following Rehabilitation In Vietnam. *Journal of zoo and wildlife medicine : official publication of the American Association of Zoo Veterinarians* **52**(1), 241–252 (2021).

**129** Adekanmbi, AJ., Adekanmbi, AA., Akinola, OB. Short Wavelength Cone Opsin Is Not Expressed in the Retina of Arboreal African Pangolin (*Manis tricuspis*). *Scientifica* **2016**, 1535490 (2016).

**130** Wilson, DE., Helgen, KM., Yun, CS., Giman, B. Small mammal survey at two sites in Planted Forest Zone, Bintulu, Sarawak. *Malayan Nature Journal* **59**(2), 165–187 (2006).

**131** Angelici F., Egbide, B., Akani, G. Some new mammal records from the rainforests of south-eastern Nigeria. *Hystrix It J Mamm* **12**(1) (2001).

**132** Bobo KS. et al. Species richness, spatial distributions and densities of large- and medium-sized mammals in the northern periphery of Boumba-bek national park, southeastern Cameroon. *African Study Monographs* **49**, 91–114 (2014).

**133** Gray TN. et al. Status and conservation significance of ground-dwelling mammals in the Cardamom Rainforest Landscape, southwestern Cambodia. *Cambodian Journal of Natural History* 38–48 (2017).

**134** Dookia S. et al. Status of indian pangolin (Manis crassicaudata) in the arid part of thar desert of Rajasthan. *Tigerpaper* **XXXI**(3), 9–10 (2004).

**135** Laing, AB., Edeson, JF., Wharton, RH. Studies on filariasis in Malaya: the vertebrate hosts of Brugia malayi and B. pahangi. *Annals of tropical medicine and parasitology* **54**, 92–99 (1960).

**136** Nguyen VT., Leanne, C., Tran, QP. Sunda pangolin *manis javanica* husbandry guidelines. *Cuc Phuong National Park.* (2010).

**137** Chong, JL. et al. Sunda pangolin *Manis javanica* (Desmarest, 1822). *Pangolins*, 89-108 (2020).

**138** Shepherd, CR.., Connelly, E., Hywood, L., Cassey, P. Taking a stand against illegal wildlife trade: the Zimbabwean approach to pangolin conservation. *Oryx* **51**(2), 280–285 (2017).

**139** Cabana, F., & Tay, C. The addition of soil and chitin into Sunda pangolin (*Manis javanica*) diets affect digestibility, faecal scoring, mean retention time and body weight. *Zoo Biol.* **39,** 29–36 (2020).

**140** Imam, A. et al. The brain of the tree pangolin (*Manis tricuspis*). I. General appearance of the central nervous system. J. Comp. *Neurol.* **525**, 2571–2582 (2017).

**141** Imam, A., Ajao, MS., Bhagwandin, A., Ihunwo, AO. & Manger, PR. The brain of the tree pangolin (*Manis tricuspis*). IV. *The hippocampal formation. J. Comp. Neurol.* **527**, 2393–2412 (2019).

**142** Anadu, PA., Elamah, PO. & Oates, JF. The bushmeat trade in southwestern Nigeria: A case study. *Hum. Ecol*, **16**, 199–208 (1988).

**143** Akpan, A., Esenowo, IK., Egwali, EC. & James, SU. The checklist and abundances of Small Mammals in Idu, Akwa Ibom State, Nigeria. *Journal of Applied Sciences and Environmental Management* **19** (2015).

**144** Tarmizi, R., Keng Chee, Y., Sipangkui, S., Zainuddin, ZZ. & Fitri, W. The Comparison of Semen Collection in Electroejaculation, Rectal Massage and Combination of Both Methods in the Critically Endangered Malayan Pangolin, *Manis javanica*. *Animals : an open access journal from MDPI* **10** (2020).

**145** Weiskopf, SR. et al. The conservation value of forest fragments in the increasingly agrarian landscape of Sumatra. *Environ. Conserv.* **46**, 340–346 (2019).

**146** Sayer, JA. & Green, AA. The distribution and status of large mammals in Benin. *Mammal Rev*, **14**, 37-50 (1984).

**147** Li, YM. & Dianmo, L. The dynamics of trade in live wildlife across the Guangxi border between China and Vietnam during 1993-1996 and its control strategies. *Biodivers. Conserv.* **7**, 895-914 (1998).

**148** Kwak, ML., Hsu, C., Douay, G. & Ahmad, AA. The first authenticated record of the pangolin tick *Amblyomma javanense* (Acari: Ixodidae) in Singapore, with notes on its biology and conservation. *Exp. Appl. Acarol.* **76**, 551–557 (2018).

**149** Beja P. et al. The Mammals of Angola. *Biodiversity of Angola*. 357-443 (2019).

**150** Esselstyn, JA., Widmann, P. & Heaney, LR. The mammals of Palawan Island, Philippines. *Proc. Biol. Soc. Wash.* **117**, 271–302 (2004).

**151** Coulson I. The pangolin {*Manis temmincki Smuts*, 1835) in Zimbabwe. *Afr. J. Ecol.* (1989).

**152** Hassan, M., Sulaiman, MH. & Lian, CJ. The prevalence and intensity of Amblyomma javanense infestation on Malayan pangolins (*Manis javanica Desmarest*) from Peninsular Malaysia. *Acta Trop* **126**, 142–145 (2013).

**153** Subedi N., Lee S. & Acharya K.The Status of Nepal's Mammals: The National Red List Series. *Department of National Parks and Wildlife Conservation* (2011).

**154** Nash, HC. et al. The Sunda pangolin in Singapore: a multi-stakeholder approach to research and conservation. *Pangolins.* 411–425 (2020).

**155** Pillai KM. Tick infestation in an Indian pangolin (*Manis crassicaudata*). *Indian Veterinary Journal* **74**(1), 71-72 (1997).

**156** Challender, DWS., Challender, DW., Thai, NV., Jones, M., & May, L. Time-Budgets and Activity Patterns of Captive Sunda Pangolins (*Manis javanica*). *Zoo Biol.* **31**, 206–218 (2012).

**157** Ishige, T. et al. Tropical-forest mammals as detected by environmental DNA at natural saltlicks in Borneo. *Biol. Conserv.* **210,** 281–285 (2017).

**158** McEvoy, JF. et al. Two sides of the same coin - Wildmeat consumption and illegal wildlife trade at the crossroads of Asia. Biol. *Conserv*. **238**, 9 (2019).

**159** Fopa, GD. et al. Understanding Local Ecological Knowledge, Ethnozoology, and Public Opinion to Improve Pangolin Conservation in the Center and East Regions of Cameroon. *J. Ethnobiol.* **40**, 234–251 (2020).

**160** Boakye, MK., Boakye, MK., Kotzé, A., Dalton, DL. & Jansen, R. Unravelling the Pangolin Bushmeat Commodity Chain and the Extent of Trade in Ghana. *Hum. Ecol.* **44**, 257–264 (2016).

**161** Algewatta, HR. et al. Updates on the Morphometric Characterization of Indian Pangolin (*Manis crassicaudata*) in Sri Lanka. *Animals* **11,** 17 (2021).

**162** Tee, SL. et al. Urban forest fragmentation impoverishes native mammalian biodiversity in the tropics. *Ecol. Evol.* **8**, 12506–12521 (2018).

**163** Soewu, DA. & Ayodele, IA. Utilisation of Pangolin (*Manis sps*) in traditional Yorubic medicine in Ijebu province, Ogun State, Nigeria. *J. Ethnobiol. Ethnomed.* **5**, 11 (2009).

**164** Jansen, R. et al. White-bellied pangolin *Phataginus tricuspis* (Rafinesque, 1820). *Academic Press Ltd-Elsevier Science Ltd,* **125**, (2020).

**165** Gubbi S. & Linkie, M. Wildlife hunting patterns, techniques, and profile of hunters in and around Periyar Tiger Reserve. *J. Bombay Nat. Hist. Soc* **109**, 165–172 (2012).

**166** Duckworth, WD., Salter, RE., & Khounboline, K. Wildlife in Lao PDR: 1999 status report. *IUCN, Vientiane, Lao PDR* (1999).

**167** Cruz, RM., Beukel, DV., Lacerna-Widmann, I., Schoppe, S. & Widmann, P. Wildlife Trade in Southern Palawan, Philippines. *Banwa* **4**, 12–26 (2007).

**168** Pantel, S., Chin, SY. Proceedings of the Workshop on Trade and Conservation of Pangolins Native to South and Southeast Asia. *TRAFFIC Southeast Asia* (2009).

**169** Baylis, HA. XLIII. —On some parasitic worms from Java, with remarks on the Acanthocephalan genus Pallisentis. *Ann.Mag.nat.Hist* **12**, 443–449 (2009).

**170** 杨雪婷. 食蚁类哺乳动物肠道微生物宏基因组学研究 [硕士]: 云南大学,(2017).

**171** 高天柱,等. 长尾穿山甲仿生态圈养行为保育研究. *现代农业科技* **16,** 227–229 (2017).

**Supplementary Table 8. The gene or protein used to construct the phylogenetic tree of viruses**

| Viruses | Gene or protein used for phylogenetic analysis | The trimmed length after alignment used for phylogenetic analysis |
|---|---|---|
| *Pneumoviridae* | RdRp protein | 2164 aa |
| *Mammalian orthoreovirus* | L1 gene | 3799 aa |
| *Japanese encephalitis virus* | E gene | 1500 bp |
| *Respirovirus* | RdRp protein | 2220 aa |
| *Orthorubulavirus* | RdRp protein | 2255 aa |
| *Rotavirus A* | VP1 gene | 3258 bp |
| *Chikungunya virus* | CHIKVgp2 gene | 1317 bp |
| *Betacoronavirus* | RdRp domain | 2790 bp |
| *Parvoviridae* | Capsid protein | 644 aa |
| *Astroviridae* | Capsid protein | 431 aa |
| *Circovirus* | Capsid protein | 177 aa |
| *Cyclovirus* | Capsid protein | 134 aa |
| unclassified *Flaviviridae* | RdRp protein | 866 aa |
| *Orthomyxoviridae* | Nucleoprotein | 291 aa |
| *Sapovirus* | RdRp protein | 2175 aa |
| *Nairoviridae* | RdRp protein | 3284 aa |
| *Hunnivirus* | RdRp protein | 1706 aa |
| *Phasmaviridae* | Glycoprotein | 543 aa |
| *Papillomaviridae* | L1 protein | 405 aa |
| *Pestivirus* | RdRp domain | 11958 bp |
| *Coltivirus* | RdRp protein | 1250 aa |
| *Genomoviridae* | Replication-associated protein | 311 aa |
| *Rhabdoviridae* | Nucleoprotein | 402 aa |
| *Senecavirus* | RdRp protein | 1902 aa |
| *Picobirnaviridae* | Capsid protein | 364 aa |
| *Shanbavirus* | RdRp protein | 1874 aa |
| *Tettorquevirus* | ORF1 protein | 561 aa |
| *Phenuiviridae* | RdRp protein | 1689 aa |
| *Etatorquevirus* | ORF1 protein | 470 aa |
