# Supplementary material for: Systematic review and integrated data analysis reveal diverse pangolin-associated microbes with infection potential
Source: Nat Commun. 2023 Oct 25;14:6786. doi: 10.1038/s41467-023-42592-w (PMC10600157; doi:10.1038/s41467-023-42592-w)
Supplement: Supplementary file 4 — Reporting Summary [file 41467_2023_42592_MOESM4_ESM.pdf]

nature portfolio

Corresponding author(s): Xiao-Ming Cui, Lin Zhao, Wu-Chun Cao

Last updated by author(s): Sep 19, 2023

Reporting Summary

Nature Portfolio wishes to improve the reproducibility of the work that we publish. This form provides structure for consistency and transparency in reporting. For further information on Nature Portfolio policies, see our Editorial Policies and the Editorial Policy Checklist.

Statistics

For all statistical analyses, confirm that the following items are present in the figure legend, table legend, main text, or Methods section.

n/a	Confirmed	
☐	☒	The exact sample size (*n*) for each experimental group/condition, given as a discrete number and unit of measurement
☒	☐	A statement on whether measurements were taken from distinct samples or whether the same sample was measured repeatedly
☐	☒	The statistical test(s) used AND whether they are one- or two-sided<br>*Only common tests should be described solely by name; describe more complex techniques in the Methods section.*
☒	☐	A description of all covariates tested
☒	☐	A description of any assumptions or corrections, such as tests of normality and adjustment for multiple comparisons
☐	☒	A full description of the statistical parameters including central tendency (e.g. means) or other basic estimates (e.g. regression coefficient) AND variation (e.g. standard deviation) or associated estimates of uncertainty (e.g. confidence intervals)
☒	☐	For null hypothesis testing, the test statistic (e.g. *F*, *t*, *r*) with confidence intervals, effect sizes, degrees of freedom and *P* value noted<br>*Give P values as exact values whenever suitable.*
☒	☐	For Bayesian analysis, information on the choice of priors and Markov chain Monte Carlo settings
☒	☐	For hierarchical and complex designs, identification of the appropriate level for tests and full reporting of outcomes
☒	☐	Estimates of effect sizes (e.g. Cohen's *d*, Pearson's *r*), indicating how they were calculated

Our web collection on statistics for biologists contains articles on many of the points above.

Software and code

Policy information about availability of computer code

Data collection	No commercial, open source or custom code was used for data collection.
Data analysis	ArcGIS (v10.6): thematic map production.<br>R (v4.2.1): statistical test and data visualization.<br>meta package (v6.5-0): meta-analysis.<br>ggtree (v3.4.4), phangorn (v2.9.0), and ggplot2 (v3.4.2) packages: visualizing the trees and determining the midpoint as the root of the phylogenetic tree.<br>MAFFT (v7.505): sequences alignment.<br>trimAL (v1.4.rev15): ambiguously aligned regions trimming.<br>IQ-TREE (v2.2.0.3): phylogenetic tree estimation.

For manuscripts utilizing custom algorithms or software that are central to the research but not yet described in published literature, software must be made available to editors and reviewers. We strongly encourage code deposition in a community repository (e.g. GitHub). See the Nature Portfolio guidelines for submitting code & software for further information.

Data

Policy information about availability of data

All manuscripts must include a data availability statement. This statement should provide the following information, where applicable:

- Accession codes, unique identifiers, or web links for publicly available datasets
- A description of any restrictions on data availability
- For clinical datasets or third party data, please ensure that the statement adheres to our policy

Data were collected from multiple sources, including literature review, related websites, and GenBank, GISAID, and NGDC. The sequences of pangolin-associated microbes used in this study are available in the GenBank (https://www.ncbi.nlm.nih.gov/nuccore/), GISAID (https://www.gisaid.org/), and NGDC (https://ngdc.cncb.ac.cn/) database under accession numbers shown in Supplementary Table 3.Source data are provided with this paper.

Research involving human participants, their data, or biological material

Policy information about studies with human participants or human data. See also policy information about sex, gender (identity/presentation), and sexual orientation and race, ethnicity and racism.

Reporting on sex and gender	N/A
Reporting on race, ethnicity, or other socially relevant groupings	N/A
Population characteristics	N/A
Recruitment	N/A
Ethics oversight	N/A

Note that full information on the approval of the study protocol must also be provided in the manuscript.

Field-specific reporting

Please select the one below that is the best fit for your research. If you are not sure, read the appropriate sections before making your selection.

☐ Life sciences ☐ Behavioural & social sciences ☒ Ecological, evolutionary & environmental sciences

For a reference copy of the document with all sections, see nature.com/documents/nr-reporting-summary-flat.pdf

Ecological, evolutionary & environmental sciences study design

All studies must disclose on these points even when the disclosure is negative.

Study description	This study integrated data from multiple sources to describe the distribution and spectrum of microbes harbored by pangolins.
Research sample	We followed the PRISMA (Preferred Reporting Items for Systematic Reviews and Meta-Analyses) protocol for study selection and inclusion in the literature review and meta-analysis. Data were collected from multiple sources, including literature review, related websites, and GenBank, GISAID, and NGDC. A total of 2337 records with locations of different pangolin species reported in 60 countries around the world and 142 microbial records were included in this study.
Sampling strategy	The data collocation and analysis were reported according to the PRISMA guidelines. Sample size was not predetermined as data in analysis were extracted from all studies that met the inclusion criteria. Sample size (number of study) was checked for all groups, and meta-analyses were not conducted if number of study was only one.
Data collection	Data from publications were extracted by two independent reviewers (Xiao-Yang Wang and Bao-Yu Wang) through searching electronic databases (PubMed, China National Knowledge Infrastructure, and the WanFang database). The sequences with related information of pangolin-associated microbes were obtained from GenBank, Global Initiative on Sharing All Influenza Data, and National Genomics Data Center by two independent reviewers (Run-Ze Ye and Yu-Yu Li).
Timing and spatial scale	The literature search were conducted in February 16, 2023. The included studies were published between 1888 and 2023. The locations of pangolin are displayed in Figure 2.
Data exclusions	Articles were excluded if sufficient information about the distribution or associated microbes of pangolins was unavailable, or they were duplicated.
Reproducibility	The data collocation and analysis were reported according to the PRISMA guidelines. The methods of data collection and analysis are

Reproducibility	described in the Methods in detail to enhance reproducibility the data. To ensure the reliability of the research results, two individuals extracted the literature information and checked the information with each other.
Randomization	Not applicable. This study is a analysis on integrated data from multiple sources. Randomization is not applicable to meta-analysis.
Blinding	Not applicable. This study is a analysis on integrated data from multiple sources. Blinding is not applicable to meta-analysis.

Did the study involve field work? ☐ Yes ☒ No

Reporting for specific materials, systems and methods

We require information from authors about some types of materials, experimental systems and methods used in many studies. Here, indicate whether each material, system or method listed is relevant to your study. If you are not sure if a list item applies to your research, read the appropriate section before selecting a response.

Materials & experimental systems

n/a	Involved in the study
☒	☐ Antibodies
☒	☐ Eukaryotic cell lines
☒	☐ Palaeontology and archaeology
☒	☐ Animals and other organisms
☒	☐ Clinical data
☒	☐ Dual use research of concern
☒	☐ Plants

Methods

n/a	Involved in the study
☒	☐ ChIP-seq
☒	☐ Flow cytometry
☒	☐ MRI-based neuroimaging
